# Corticohippocampal Dysfunction In The *OBiden* Mouse Model Of Primary Oligodendrogliopathy

**DOI:** 10.1038/s41598-018-34414-7

**Published:** 2018-10-31

**Authors:** Daniel Z. Radecki, Elizabeth L. Johnson, Ashley K. Brown, Nicholas T. Meshkin, Shane A. Perrine, Alexander Gow

**Affiliations:** 1 Center for Molecular Medicine and Genetics, School of Medicine, Wayne State University, Detroit, MI 48201 USA; 2 Institute of Gerontology, Wayne State University, Detroit, MI 48202 USA; 3 Department of Psychiatry and Behavioral Neurosciences, School of Medicine, Wayne State University, Detroit, MI 48201 USA; 4 Carman and Ann Adams Department of Pediatrics, School of Medicine, Wayne State University, Detroit, MI 48201 USA; 5 Department of Neurology, School of Medicine, Wayne State University, Detroit, MI 48201 USA; 6 Helen Wills Neuroscience Institute, University of California, Berkeley, CA 94720 USA; 7 Present Address: Department of Comparative Biosciences, University of Wisconsin-Madison School of Veterinary Medicine, Madison, WI 53706 USA

## Abstract

Despite concerted efforts over decades, the etiology of multiple sclerosis (MS) remains unclear. Autoimmunity, environmental-challenges, molecular mimicry and viral hypotheses have proven equivocal because early-stage disease is typically presymptomatic. Indeed, most animal models of MS also lack defined etiologies. We have developed a novel adult-onset oligodendrogliopathy using a delineated metabolic stress etiology in myelinating cells, and our central question is, “how much of the pathobiology of MS can be recapitulated in this model?” The analyses described herein demonstrate that innate immune activation, glial scarring, cortical and hippocampal damage with accompanying electrophysiological, behavioral and memory deficits naturally emerge from disease progression. Molecular analyses reveal neurofilament changes in normal-appearing gray matter that parallel those in cortical samples from MS patients with progressive disease. Finally, axon initial segments of deep layer pyramidal neurons are perturbed in entorhinal/frontal cortex and hippocampus from *OBiden* mice, and computational modeling provides insight into vulnerabilities of action potential generation during demyelination and early remyelination. We integrate these findings into a working model of corticohippocampal circuit dysfunction to predict how myelin damage might eventually lead to cognitive decline.

## Introduction

Multiple Sclerosis (MS) is a multi-faceted disease for which the relevance of pathology to etiology continues to evolve. Early work in the 19^th^ century identified white matter (WM) and gray matter (GM) lesions and axon loss as prominent disease characteristics, and 20^th^ century studies largely focused on autoimmune- and virally-mediated etiologies. Extensive clinical trials data for recent disease-modifying therapies report strong suppression or modulation of peripheral immune system infiltration into the CNS, but success in halting disease progression has been unexpectedly variable or modest^1–3^. Thus, expectations that the conventional view of MS etiology (defined as the “outside-in” model) is too narrowly focused or incomplete^4–7^ have led to an alternative “inside-out” model for pathogenesis^8^.

Features of MS include oligodendrocyte metabolic stress or cell loss, microglial and astroglial activation, demyelination and remyelination, ventricular enlargement and cortical atrophy, axonal spheroids, abnormal neurofilament biology and the large scale infiltration and activation of peripheral leukocyte populations^5,9–13^. Functional deficits, including limb paresis, bladder dysfunction, diminished coordination, altered circadian rhythms, cognition and capricious emotional state are some of the common clinical signs in MS patients. Cognitive and behavioral changes often emerge in progressive disease and include impaired learning, memory and information processing speed, abnormal electroencephalography and neuropsychiatric changes^14–19^.

Unexpected disease progression in MS patients undergoing contemporary immunosuppressive therapies, which all but eliminate nascent WM lesion activity, has rekindled interest in apparent nonimmune-mediated GM damage and neurodegeneration as salient features of pathophysiology^20–22^. Several animal models have been developed or optimized to investigate the relationships between myelin-producing oligodendrocytes, the axons they ensheath and altered neuron function following oligodendrocyte damage or death^23–27^. And while these models facilitate exploration of MS pathobiology, a major limitation is that the associated pathogenic mechanisms are poorly characterized or unknown; thus, they shed little light on etiology.

Multiple sclerosis is typically regarded as a chronic-progressive disease, with the effects of episodic pathology accumulating over years to decades. We have developed a chronic-progressive model with a defined etiology – that of adult-onset primary oligodendrocyte damage leading to cell dysfunction or death, demyelination/ remyelination and eventual neuronal dysfunction – to characterize secondary GM pathology for comparison with disease in patients. A major goal is to resolve the question: how much of the pathobiology of MS can be recapitulated by a primary metabolic defect in oligodendrocytes? Answering this question may yield insights into whether adaptive immune activation and CNS invasion are etiologic for MS or, rather, reflect inside-out processes with heightened susceptibility to inflammation that is dependent on immune haplotype or other genetic/environmental modifiers^28,29^.

Herein, we characterize the pathobiology of the *OBiden* (*OBi*) mouse in which missense mutant *Plp1* gene expression is used to drive oligodendrocyte metabolic stress and pathogenesis beginning in adults after myelinogenesis is complete^30^. Widely distributed subsets of mature, myelinating perivascular oligodendrocytes succumb to this insult, and repetitive induction exacerbates chronic pathology, with eventual disease progression. Coincidentally, diagnostic criteria consistent with primary progressive MS (PPMS) have been reported for an obligate carrier female with a missense mutation in the *PLP1* gene^31^. Moreover, a recent epitranscriptomics pilot study at the Universities of Connecticut and Stanford suggests that aberrant somatic RNA editing may give rise to expression of missense mutant PLP1 isoforms in active lesions from two MS patients^7^. In this light, it is tempting to speculate that MS may arise clinically, not from immune invasion per se, but rather as a consequence of the interplay between chronic CNS metabolic disease and immune haplotype. In general terms, this concept may be sufficiently broad to account for well-known correlations between disease activity and various environmental or genetic factors – climate, gender, pregnancy or switching from one anti-immune treatment to another.

## Materials and Methods

The Supplementary Information include detailed experimental procedures and analyses.

### Animal care and housing

All procedures with mice were pre-approved by the Wayne State University IACUC committee. All experiments herein comply with IACUC regulations and the Guide for the Care and Use of Laboratory Animals (8^th^ edn, 2011). Mice were maintained under standard housing conditions in a 12 h light/dark cycle with ad libitum standard rodent chow and tap water. Any mice reaching a moribund state or losing more than 20% of their body weight were euthanized, as mandated by our approved IACUC protocols. Mice were raised in the DLAR vivarium at Wayne State University. In general, *OBi* mice and littermate controls were reared to adulthood (2 mo) for baseline data (Supplementary Fig. S1) just prior to weekly orogastric gavage with 150 mg/kg of tamoxifen (freshly dissolved in corn oil) until 12 mo. Mice underwent multiple tests before euthanasia and tissue harvest at 12 mo.

### *OBi* genetics and genotyping

A (493 bp product) *Plp1*^*i*.*msd*^ inducible allele was generated from the endogenous *X*-linked *Plp1* gene using homologous recombination to insert two changes^30^, 1) a *floxed PGKneo* cassette on the antisense strand at the 5′ end of intron 6, and 2) a naturally-occurring point mutation in codon 243 of exon 6 (C → T), which encodes the A243V missense mutation identified in short-lived myelin-synthesis-deficient (*msd*) mice and connatal Pelizaeus-Merzbacher disease (Supplementary Fig. S1)^32,33^. Mice were sibling mated to eliminate wild type endogenous *Plp1* alleles from males and females in the colony.

To remove the *PGKneo* antisense suppression cassette from the *Plp1*^*i*.*msd*^ allele in myelinating oligodendrocytes and induce expression of the missense PLP1^*msd*^ protein, we generated a transgene encoding tamoxifen-inducible creER^T2^ fusion protein under control of the *Mbp* promoter (*McreG*)^34^. Importantly, *McreG* lacks the promoter-enhancer for Schwann cell expression and is not expressed in the PNS^35^. This allele is maintained as hemizygous in one parent from each breeding pair so that 50% of pups generated are hemizygous.

Finally, we dosage-compensate for a lack of wild type *Plp1* gene expression using the *Plp1* overexpressor (*Plp1*^*OeX*^) transgene #66 allele^36^. Expression of this allele in hemizygous mice approximates that of the endogenous *Plp1* gene in wild type mice (Supplementary Fig. S1). This allele is maintained as homozygous in one parent from each breeding pair so that all progeny are hemizygous.

All mice used in the current study are males, which ensures genetic homogeneity of the *X*-chromosome at the endogenous *Plp1* locus. *OBiden* (*OBi*) mice harbor all three engineered alleles, *Plp1*^*i*.*msd*^*::McreG::Plp1*^*OeX*^, while littermate controls lack the *McreG* allele, *Plp1*^*i*.*msd*^*::Plp1*^*OeX*^. In Supplementary Fig. S1, we also included an additional cohort of control mice for some experiments; genetically, these male mice are *Plp1*^*i*.*msd*^, so they lack a functional *Plp1* gene and do not express the encoded gene products. See Supplemental Methods for primers used in genotyping.

### Patient autopsy tissue samples

Deidentified brain autopsy samples from frontal cortex of PPMS or secondary progressive MS (SPMS) and non-neurological patients were provided by the Human Brain and Spinal Fluid Research Center (University of California, LA). The curators of the bank performed a detailed neuropathological examination of each patient, including lesion subtypes^37^, final clinical status and summary reports (see Supplementary Information). All experiments with these samples complies with the regulations of the Wayne State University Medical Institutional Review Board (M1, expedited IRB# 042414M1X, Protocol# 1404012948).

### Interhemispheric electroencephalograms (EEGs)

Control and *OBi* mice were anesthetized at 2, 6, 8, 10 and 12 mo with 2,2,2-tribromoethanol (350 mg/kg) in sterile saline for two channel EEG recording using subdermal electrodes (Grass, Fisher) in an A1-Cz-A2 montage. Body temperature was maintained using an isothermal pad at 39 °C (Braintree). Signals were pre-amplified (100,000×) and sampled at 250 Hz using an Intelligent Hearing Systems Universal box with SmartEP CAM software (ver. 2.0, Intelligent Hearing Systems, Miami, FL), and processed offline using custom-built MATLAB (MathWorks, Inc., Natick, MA) scripts with the FieldTrip toolbox^38^. Unprocessed EEGs were filtered with 0.5 Hz high-pass and 50 Hz low-pass finite impulse response (FIR) filters, and the 6 Hz electrocardiogram was minimized using discrete Fourier transform. Continuous data were epoched into non-overlapping 2 sec segments for automated artifact detection and spectral analysis. Epochs were discarded if they contained channel-wise mean analytic amplitudes exceeding 5 S.D. (>10 ms) of the total recording.

Data segments were zero-padded to 10 sec and spectral analysis was performed using 30 logarithmically-spaced bandpass FIR filters from 1–30 Hz, with 1/3 fractional bandwidth (rounded up to the nearest 1 Hz, 5 Hz maximum). The analytic amplitude envelope was first extracted using the Hilbert transform and squared to produce power values, and per-epoch outputs were averaged and z-scored against all epochs to identify those in which power exceeded 2 S.D. of the total recording (i.e., signal-to-noise α < 0.05). These epochs were removed from further analysis, and all remaining power outputs were averaged per frequency and channel. The Hilbert transform was then used to extract the complex values from all clean epochs, from which A1-A2 coherence was quantified in the time domain^39^ and averaged per frequency bin. Thus, 80.4 ± 9.1% (mean ± S.D.) of the continuous data were included in the final power and coherence analyses. Finally for each mouse, the median coherence of the bins within each frequency band (rather than the mean coherence) was used for ANOVA to minimize outlier effects associated with the platykurtic distributions of the data (i.e. excess kurtosis <−3 for most groups).

### Immunocytochemistry, histology and morphometric analyses

Mice were perfused intracardially for 15 min with freshly prepared 4% paraformaldehyde in 0.1 M phosphate buffer, pH7.4. Brains were dissected, sliced into 5 × 2 mm coronal blocks using a brain mold (Braintree, Chicago, IL) beginning at the caudal end of the olfactory bulbs (+3.6 mm to −6.6 mm Bregma) and embedded in O.C.T. for cryostat sectioning at 10 μm and thaw-mounting on Superfrost slides (Fisher). Sections were thawed in PBS, permeabilized with methanol or Triton X-100 for 20–30 min, blocked for 30 min and incubated with primary antibodies (Supplementary Table 1) overnight. After washing in PBS, sections were incubated at 25 °C in fluorescently-labeled secondary antibodies (Southern Biotech, Birmingham, AL) for 3 h and counterstained with DAPI according to published methods^40^.

### Behavioral testing

Several behavioral tests were used to assess *OBi* mice, generally at 2 (baseline), 6 and 12 mo – tail suspension, forced swim, win-shift T-maze, novel object, fear extinction and Barnes maze – according to published procedures with minor modifications^41–47^. See Supplementary Information. Inverted screen tests were performed weekly between 2–12 mo^48^.

### Western blotting

Freshly dissected mouse brains and partially-thawed human brain tissue blocks (Human Brain and Spinal Fluid Research Center) were cut into 2 mm slices using a brain mold (Braintree) and frozen on a dry ice block. Tissue samples for analysis were harvested with a 1.5 mm disposable biopsy punch (Integra Miltex, Integra, York, PA) for storage at −80 °C. Post punch brain slices were fixed with 4% paraformaldehyde, 0.1 M phosphate buffer, pH 7.2 for subsequent sectioning and examination. Tissue punches were thawed in phosphorylation-preservation buffer with protease inhibitors, sonicated for 45 sec on ice. Protein concentrations were measured (BCA, Fisher) for loading and the samples electrophoresed on SDS-polyacrylamide gels^49^ for transfer to nitrocellulose membranes as previously published^50^. Individual blots were cut horizontally into several strips so that proteins with different *M*_r_ could be probed with primary antibodies from the same blot to conserve samples (see full length blot strips in the Supplementary Information).

Blots were developed with ECL and CL-Xposure film (Fisher) and unprocessed image scans were quantified by area under the curve (AUC) using the Gel Plotter densitometry macro in ImageJ 64 (v 1.49) with selection marquees adjusted to the width of each lane. Blot AUCs were normalized using α-tubulin as an electrotransfer control. In all cases, flattening at pixel intensity peaks was not observed, indicating that film exposures were not saturating. In cases of protein doublets (e.g. NeuN), we combined the associated AUCs. The AUCs from *OBi* mice were normalized to controls as fold-changes. For all figures, western blot bands cropped from different parts of the same gel or different gels were assembled with intervening gaps (see full length blots in the Supplementary Information). Western blot figures were processed in Adobe Photoshop CS6 (Adobe Systems, San Jose, CA). When necessary, the contrast of entire blot images was adjusted prior to cropping to provide approximately uniform background intensities with other blots in the figure.

### Neuron simulations and code availability

Computational models of myelinated and demyelinated deep layer cortical neurons were developed using published parameters and data derived from the current study (see Supplementary Information). Simulations were performed using the NEURON (version 7.4) environment^51^. The code used in this study will be deposited in ModelDB (https://senselab.med.yale.edu/modeldb/) upon publication.

### Statistical tests

In this exploratory study, all statistical methods used were performed using Prism (version 7; Graphpad Software Inc., La Jolla, CA). We used: ordinary/repeated-measures 1-way/2-way ANOVA, multiple t-tests, Fisher’s exact test and regression analyses. The major findings for each dataset are specified in the associated figure legends. In all cases, values of *n* reported in the figure legends reflect the number of mice used per group (e.g. age, genotype, protein of interest), and we used balanced study designs for experiments. For simultaneous inferences from post hoc tests, we report the Holm-Sidak adjusted *p* values. When appropriate, we performed false discovery rate-based ROUT analyses^52^ and/or Box-Cox transformations^53^ of the dependent variables to maximize homoscedasticity. For hypothesis testing with the EEG dataset, we adjusted for simultaneous inferences by controlling the false discovery rate (FDR) using the Benjamini, Krieger, Yekutieli procedure^54,55^ (Q = 0.05) implemented in Prism, and we report FDR-adjusted *q* values.

## Results

To understand pathogenesis stemming from repeated episodic metabolic stress and induction of the unfolded protein response (UPR) in mature oligodendrocytes we developed the *OBi* mouse, which harbors three engineered genes (*Plp1*^*i*.*msd*^*::McreG::Plp1*^*Oex*^; Supplementary Fig. S1). In preliminary experiments, we characterized expression of the *Plp1*^*i*.*msd*^ and *McreG* genes independently, while the *Plp1*^*Oex*^ gene (Nave #66 line) has been characterized elsewhere^36^. First, we showed that developmental expression of *Plp1*^*i*.*msd*^ from mid-gestation generates a severe early lethal phenotype indistinguishable from naturally-occurring *msd* mice (Supplementary Fig. S1) and comparable to connatal Pelizaeus-Merzbacher disease, both of which involve the same missense mutation^32,33^. This anticipated result demonstrates that developmental *Plp1*^*i*.*msd*^ expression causes profound hypomyelination and shortened life span^5,24^.

We bred *McreG* (the encoded creER^T2^ fusion protein is expressed in myelinating oligodendrocytes) into *tdTomato* mice, which express EGFP in oligodendrocytes following two consecutive daily tamoxifen gavages. The frequency of EGFP^+^ cells is greatest in perivascular regions (ca. 20% of oligodendrocytes; Supplementary Fig. S1), where extravasated tamoxifen is at its highest concentration. Overall, roughly 5% of oligodendrocytes undergo recombination and express EGFP. Attempts to determine recombination frequency at the *Plp1*^*i*.*msd*^ allele using a PCR-based genomic DNA analysis (see Supplementary Fig. S1) were partially successful. We demonstrate in optic nerve from 18 month (mo) mice gavaged weekly from 2 mo that approximately 25% of oligodendrocytes have undergone recombination. Presumably, this reflects a steady state level of surviving oligodendrocytes arising from successive rounds of recombination. In contrast, we are not able to detect recombination after the two day gavage protocol used for EGFP experiments, likely because recombination occurs in such a small proportion of cells so that the PCR signal is outpaced by the *Plp1*^*OeX*^ allele present in all cells.

Western blots of 12 mo spinal cord from *OBi* mice shows the overall levels of several major myelin proteins are normal, indicating that WM damage is mild, or focal and relatively sparse (Supplementary Fig. S1). In mice harboring only the *Plp1*^*i*.*msd*^ allele, PLP1/DM-20 is not expressed (equivalent to a *Plp1*-null mouse)^56^. Levels of PLP1/DM-20 in 12 mo control and *OBi* spinal cords are similar to age-matched wild type mice that have not been treated with tamoxifen. Thus, dosage compensation from the autosomal *Plp1*^*OeX*^ transgene, (2 copies/haploid genome^36^), is equivalent to wild type endogenous *Plp1* gene expression. Further, weekly tamoxifen gavage has minimal effects on *Mbp* and *Cnp* gene expression, and presumably other myelin genes, at least to 12 mo.

### No major changes in WM function at 6 mo

To determine if *OBi* mice exhibit major deficits in myelin function, we measured sensory signal transmission times in the auditory brainstem of 2 and 6 mo old mice using evoked potentials (the auditory brainstem response, ABR)^57^. Overall interpeak latencies of evoked waves I and V (i.e. waves V – I) is often used as a proxy for conduction velocity^58^. The auditory pathway can be partitioned into a PNS component between waves I and II (II–I) and a CNS component between waves II to V (V–II). Two month baseline interpeak latencies (Supplementary Fig. S2) are indistinguishable between *OBi* and controls, but are significantly increased in *Plp1*^*i*.*msd*^ mice. Parsing these data into CNS and PNS components of the auditory pathway reveals slowed CNS transmission time in myelinated fibers from *Plp1*^*i*.*msd*^ mice (reduced conduction velocity in *Plp1*-null mice has been reported^59^), while Schwann cell sheaths are functionally normal. The ABR data obtained from *OBi* mice at 6 mo are similar to baseline.

### Interhemispheric theta coherence deficit in resting electroencephalogram recordings

**Figure 1:**
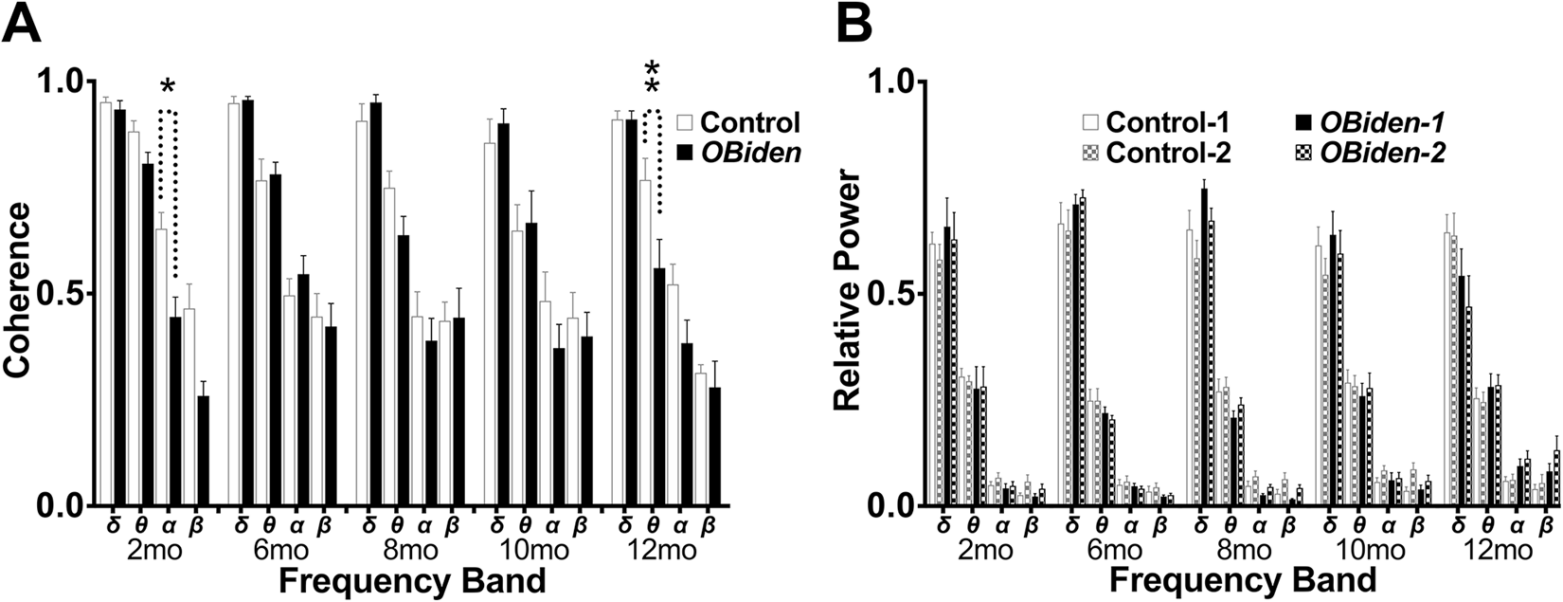
Interhemispheric electroencephalogram abnormalities in *OBi* mice. (**A**) Two channel continuous EEG data recorded from anesthetized mice using an A1-Cz-A2 montage (average of 12.3 ± 6.0 min per mouse, range: 1.90–32.17 min) were epoched at 2 sec for automated artifact rejection and spectral analysis. Real interhemispheric coherence (a) and relative power (b) were averaged across the delta (1–4 Hz), theta (4–10 Hz), alpha (10–15 Hz) and beta (15–30 Hz) frequency bands at 2 (baseline), 6, 8, 10 and 12 mo of age from control and *OBi* mice. Coherence data were analyzed by pairwise comparisons for each frequency band using two-way RM-ANOVA (frequency band × genotype) and controlled for type I errors using false discovery rate (Q = 0.05)^54^. Interhemispheric theta band coherence is reduced in *OBi* mice at 12 mo compared to littermate controls (*q* < 0.01; unadjusted *p* < 0.002) which is consistent with emerging damage to the corpus callosum and/or other myelinated commissures. In addition, we find a statistically significant decrease in alpha coherence for *OBi* mice at 2 mo (*q* < 0.03; unadjusted *p* < 0.003); however, this is likely spurious because of low relative power in alpha (<7%). Further, genotype differences in alpha are absent for the 6–12 mo time points. Overall relative power in each frequency band is stable with age (Controls: delta = 61.9 ± 1.2%, theta = 27.2 ± 0.7%, alpha = 6.1 ± 0.4%, beta = 4.7 ± 0.6%; *OBi*: delta = 64.0 ± 2.7%, theta = 25.4 ± 1.0%, alpha = 5.8 ± 0.8%, beta = 4.9 ± 1.1%) and there are no differences between EEG channels for either genotype at any age (*q* > 0.1; unadjusted *p* > 0.06).

Hearing threshold levels in mice increase rapidly beyond 6 mo; thus, to non-invasively examine WM pathology at older ages, we recorded two channel electroencephalograms (EEGs) in anesthetized *OBi* mice to measure interhemispheric coherence in signals from four frequency bands (Fig. 1A). Low frequency delta (1–4 Hz) and theta (4–10 Hz) bands dominant these EEGs and contribute approximately 90% of the spectral power from 1–30 Hz, with the remainder from the alpha (10–15 Hz) and beta (15–30 Hz) bands (Fig. 1B). With the A1-Cz-A2 montage used here, coherence measurements between the EEG channels provide a readout of myelin integrity in the major forebrain commissures connecting each hemisphere. The data indicate that although delta band coherence is normal (FDR, *q* > 0.99), theta band coherence in 12 mo *OBi* mice is reduced to 73% of control levels (*q* < 0.01).

Frequency band-specific dysfunction rules out potential non-disease related explanations such as simple changes in volume conduction. At other ages, coherence of the delta (*q* > 0.9), alpha (*q* > 0.1) and beta (*q* > 0.07) bands are comparable in both genotypes, with the exception of alpha band activity at 2 mo (*q* < 0.03). The effect potentially identifies differences between *OBi* and control mice; however, we discount this possibility because differences are not observed at other ages and the spectral power in the alpha band (i.e. reliability) is very low in anesthetized mice (<5%). Thus, together these data suggest that functional WM damage emerges between 10–12 mo in *OBi* mice.

### WM lesions, hypomyelination and activated glia

Late onset theta band EEG abnormalities in *OBi* mice suggest progressive WM pathology. Histology of *OBi* brain at 12 mo (Supplementary Fig. S3) demonstrates WM pathology, which may be focal or dispersed in multiple regions, as observed in other demyelinating mouse models^60^. For example, luxol fast blue-hematoxylin & eosin (LFB-H&E) staining of left periventricular stria medullaris WM from an *OBi* mouse shows at least two focal demyelinated lesions. Remyelination of surviving axons would likely involve differentiating migratory oligodendrocytes expressing wild type *Plp1* gene products, rather than resident metabolically-stressed cells which presumably degenerated to cause the lesions. In contrast, the contralateral tract is evenly stained and similar to controls. Internal capsule at higher magnification shows bilateral reduction in LFB staining compared to controls, indicating a region of generalized hypomyelination. To explore WM pathology further, we examined glial cell activation at the single cell level in several brain regions.

**Figure 2:**
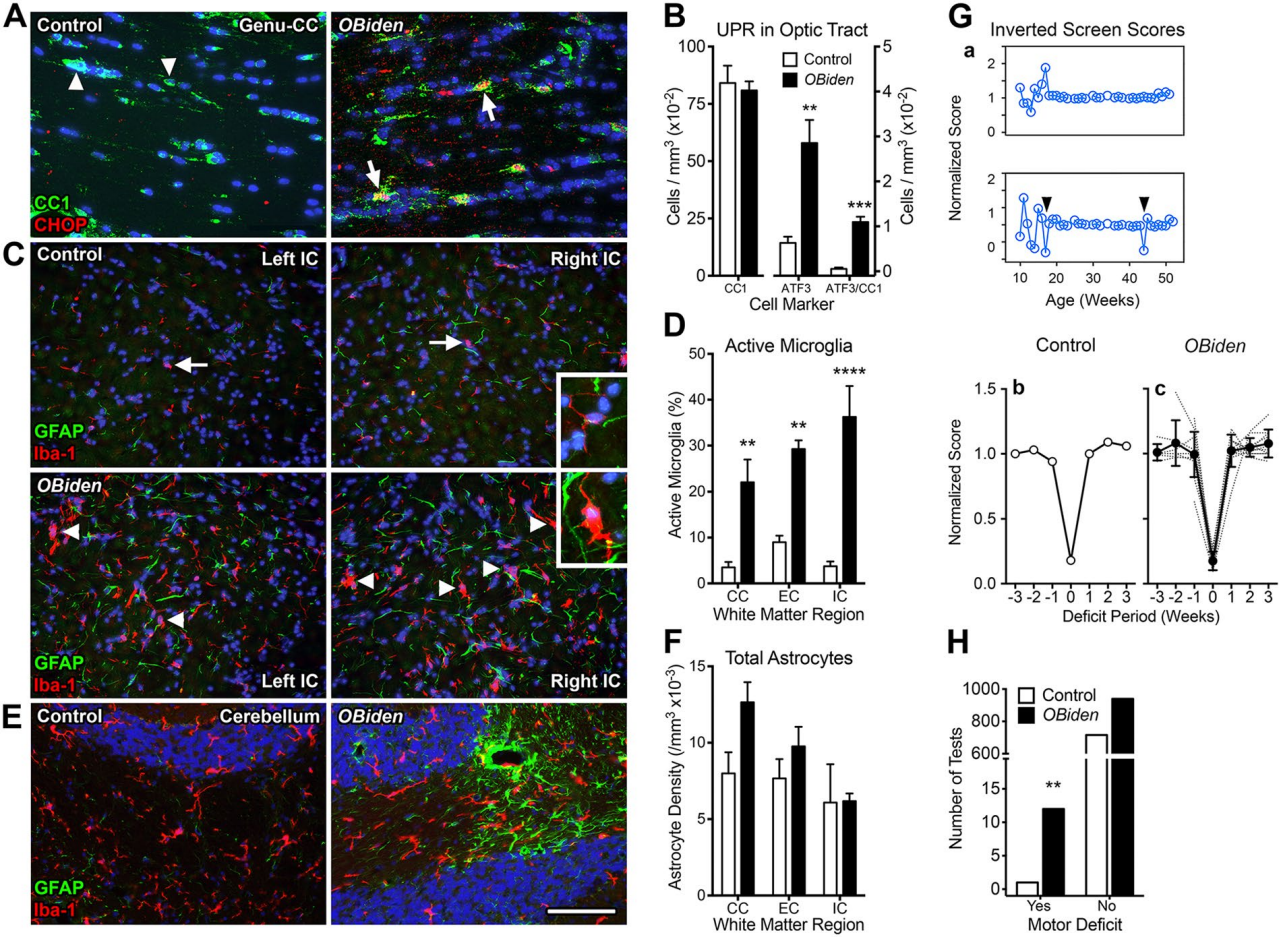
Metabolic stress in *OBi* oligodendrocytes, associated secondary gliosis and transient physical motor deficits. (**A**) Immunofluorescence antibody labeling in the genu of corpus callosum from 6 mo *OBi* mice demonstrates metabolic stress in CC1^+^ mature oligodendrocytes (green) expressing the canonical UPR transcription factor, CHOP (red). CHOP is not expressed in control oligodendrocytes. (**B**) Morphometric analysis of cells expressing CC1 and ATF3 proteins in optic tract of 12 mo *OBi* mice. The density of CC1^+^ oligodendrocytes in *OBi* mice is similar to controls (left graph; t-test, *p* > 0.7, *n* = 4). Overall expression of ATF3 is increased in *OBi* mice compared to controls (middle graph; t-test, *p* < 0.006, *n* = 4), as is ATF3 expression by CC1^+^ oligodendrocytes (right graph, *p* = 0.0001, *n* = 4). (**C**) Immunofluorescence staining for GFAP (green) and Iba-1 (red) in internal capsule from 12 mo *OBi* and control mice. Quiescent microglia dominate in control mice (arrows and inset), while activated microglia are common in *OBi* (arrowheads and inset). (**D**) Proportions of morphologically activated microglia are significantly increased in corpus callosum, external capsule and internal capsule from *OBi* mice (two-way RM-ANOVA, genotype × WM region, *p* < 0.006, *n* = 4). (**E**) Astrogliosis occasionally surrounding blood vessels is apparent in cerebellar WM of *OBi* mice. (**F**) Astrocyte densities are normal in WM tracts of *OBi* mice (two-way RM-ANOVA, genotype × WM region, *p* > 0.1, *n* = 3). (**G**) (a) Longitudinal inverted screen results for control (top) and *OBi* (bottom) mice showing instances of 3–fold changes in median performance scores (arrowheads). (b,c) Median weekly inverted screen performance scores from control (b) and *OBi* (c) mice immediately prior to and following 3–fold score changes. (**H**) Number of inverted screen motor deficits significantly exceeds expectations in *OBi* mice (Fisher’s exact test, *p* < 0.01). Scale bar in E: 40 μm (**A**); 100 μm, insets 40 μm (**C**); 100 μm (**E**). See also Supplementary Figs S1–S4.

Expression of the CHOP transcription factor and localization to the nucleus in CC1^+^ myelinating oligodendrocytes (Fig. 2A) indicates UPR induction in cells expressing mutant *Plp1* gene products^5,40^. Control mice lack the *McreG* gene and oligodendrocytes do not express CHOP; thus, weekly tamoxifen gavage per se does not activate the *Plp1*^*i*.*msd*^ allele and induce metabolic stress. Overall expression of a second UPR-related transcription factor, ATF3, is induced several-fold in optic tract of *OBi* mice; however, only CC1^+^ oligodendrocytes in *OBi* mice express ATF3 (Fig. 2B). These data are consistent with metabolic stress induction in oligodendrocytes from previous studies^5^, although expression of ATF3 per se is unlikely to be involved in pathogenesis^61^. Finally, the density of CC1^+^ oligodendrocytes in *OBi* optic tract is normal.

Microglia are activated in *OBi* mice in corpus callosum, external and internal capsule, as assessed by amoeboid morphology (Fig. 2C,D, insets), and we occasionally observe activated microglia in brainstem corticospinal tract apparently ingesting myelin debris from axons and possibly engulfing or attacking other cells (Supplementary Fig. S4). In these areas, astrocytes often appear relatively quiescent. Conversely, microglia may be relatively inactive in regions where astrocytes are focally reactive around blood vessels (Fig. 2E). Finally, overall numbers of astrocytes are constant in *OBi* mice compared to controls (Fig. 2F). Together, these data indicate that 12 mo old *OBi* mice exhibit a variety of broadly distributed and focal WM changes including activation of the innate immune system and perivascular gliotic scarring. We do not observe perivascular cuffing or accumulating small nucleated cells indicating that adaptive immune responses are minimal through 12 mo.

### Transient physical disabilities

The presence of WM pathology, in particular the apparent shadow-like plaques and perivascular gliosis, suggests *OBi* mice may develop transient motor deficits so we used weekly inverted screen tests to evaluate muscle strength in mice between 2–12 mo. We defined transient motor deficits as episodic decreases in test performance followed by recovery within 7 days (Fig. 2Ga, arrowheads in lower panel), which is rarely observed in controls (upper panel). The features of such events are similar for both controls and *OBi* mice (Fig. 2Gb,c), and are characterized by an abrupt >3–fold decrease in median test score that rapidly resolves in subsequent weeks. Collation of the test scores over 10 mo identified a single event in 800 tests from control mice, while 12 such events were detected in 1,000 tests from littermate *OBi* mice. Analysis using Fisher’s exact test indicates an increased likelihood of transient motor weakness in *OBi* mice (Fig. 2H).

### No MRI or DTI changes in GM or WM tracts

In clinical settings, MRI and DTI are commonly used to identify WM lesions that may account for transient symptoms in MS patients. In addition, longitudinal studies are used to track tissue atrophy that may be relevant to GM and WM pathology. To detect tissue atrophy or ventricular enlargement that might portend disease progression, we performed longitudinal T1/T2 weighted imaging on *OBi* mice at 2 (baseline), 6 and 12 mo and measured total brain and ventricular volumes (Supplementary Fig. S4).

Normalized total brain volume is consistent between genotypes and we did not observe signs of generalized atrophy with age. Further, segmented volume measurements of the lateral and third ventricles did not reveal consistent changes in *OBi* mice compared to controls, which suggests either that the demyelination/remyelination pathology is insufficient to cause brain atrophy or that disease progression in these mice is at an early stage by 12 mo. We also used DTI to assess WM tracts in 12 mo *OBi* mice (Supplementary Fig. S5) and found no abnormalities in fractional anisotropy (FA) of corpus callosum, external capsule or internal capsule. This suggests the DTI and FA measures are not sufficiently sensitive to detect the myelin changes observed using other techniques. The FA measurements in GM regions immediately adjacent to external capsule (thalamus and cortex) are substantially lower than WM, as expected, but not different between genotypes.

### MS-like cortical axon pathology

Most contemporary clinical studies of MS patients and animal models focus on WM pathology, although symptoms associated with disease progression – GM lesions, dystrophic axons, axonal swellings/spheroids or other degenerative changes – are the most distressing for patients^20,22,62^. To determine if GM pathology is also present in *OBi* mice, we examined Bielschowsky silver stained cortical sections from 12 mo *OBi* mice.

**Figure 3:**
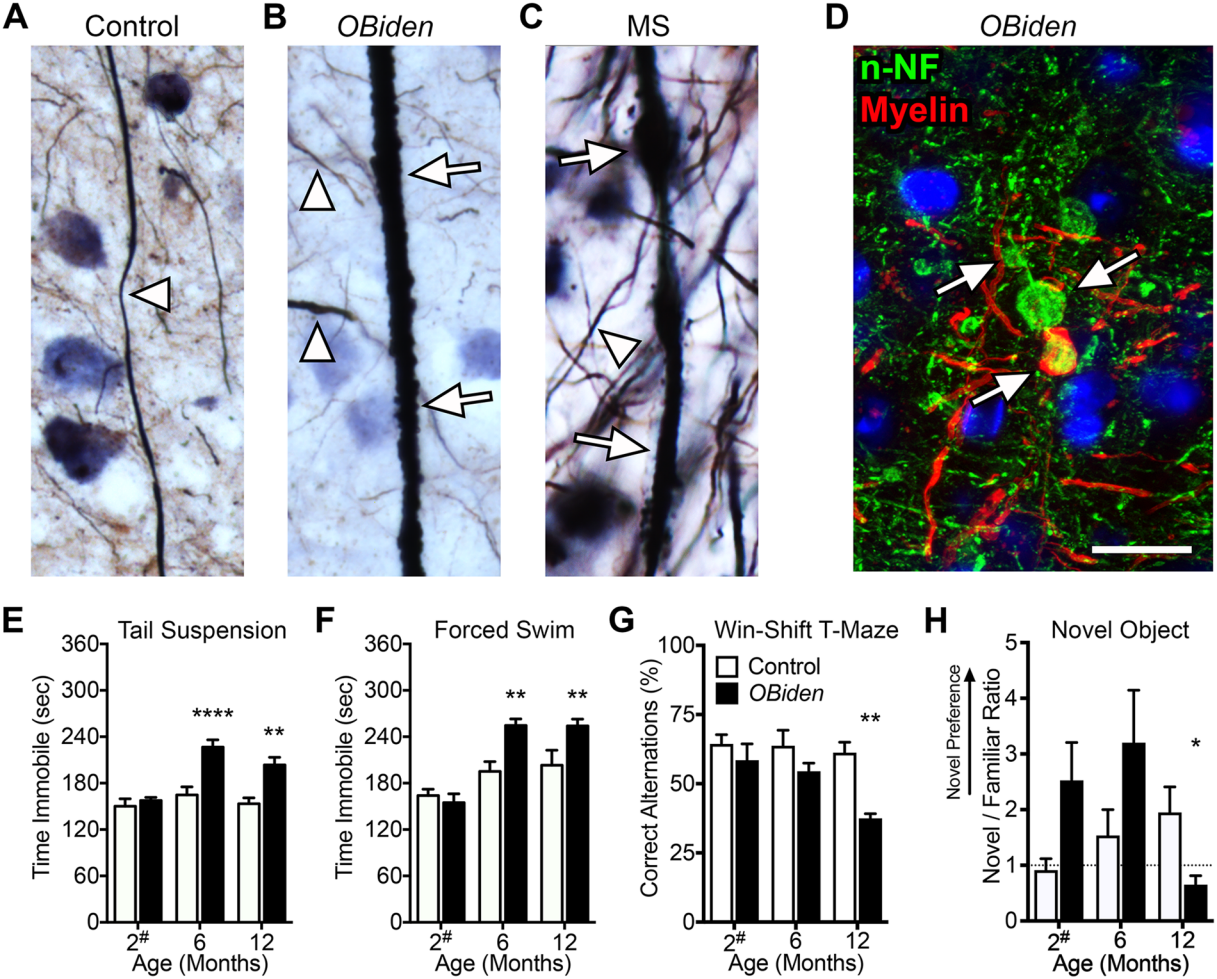
Cortical pathology in *OBi* mice and associated cognitive deficits. (**A**) Bielschowsky silver stain for 12 mo control mouse GM axons (arrowhead). (**B**) 12 mo *OBi* mouse showing normal-appearing axons (arrowheads) and a dystrophic axon with surface membrane blebbing (arrows). (**C**) Normal (arrowhead) and dystrophic axons (arrows) in normal-appearing GM (NAGM) from an MS patient (MS-4663; Supplementary Fig. S7). (**D**) Cortical immunofluorescence labeling of 12 mo *OBi* mouse cortex for non-phosphorylated neurofilament (n-NF, green) and myelin (red) shows three adjacent spheroids, two unmyelinated (upper) and one myelinated (lower). The absence of an axon exiting the lower spheroid suggests axon transection. (**E**,**F**) Depression-like endophenotype in *OBi* mice. Tail suspension (**E**) and forced swim (**F**) tests show that *OBi* mice are comparable to controls during 2 mo baseline tests (ordinary two-way ANOVA, genotype × age, *p* > 0.56: E,F *n* = 10). *OBi* mice exhibit increased immobility at 6 and 12 mo compared to controls for tail suspension (E: *p* < 0.003, 7 ≤ *n* ≤ 12) and forced swim (F: *p* < 0.01, 8 ≤ *n* ≤ 10). (**G**) Win-Shift T-Maze foraging test shows no genotype differences at 2 and 6 mo (ordinary two-way ANOVA, genotype × age, *p* > 0.2, 8 ≤ *n* ≤ 10), but reveals reduced recognition memory and alternating exploration of the goal arms for *OBi* mice at 12 mo (*p* < 0.002, *n* = 8). (**H**) The novel object test shows a recognition memory deficit in 12 mo *OBi* mice (ordinary two-way ANOVA, genotype × age, 2 and 6 mo, *p* > 0.08 7 ≤ *n* ≤ 8; 12 mo, *p* < 0.05, 9 ≤ *n* ≤ 11). Scale bar in D, 20 μm (for A–D). See also Supplementary Figs S3–S6.

In control cortex, thin relatively linear axon profiles with smooth surfaces are observed throughout the neuropil (Fig. 3A; arrowhead). Similar normal-appearing axons are observed in *OBi* mice (Fig. 3B); however, we also find abnormally thick and dystrophic axons with rough surfaces, suggesting membrane blebbing (arrows), that are reminiscent of dystrophic axons in MS cortex (Fig. 3C). In addition, we observe axonal spheroids and transections in *OBi* mice (Fig. 3D), which are similar to spheroids in MS and other *Plp1* mutant mice, and likely reflect the convergent pathology of demyelination from multiple causes^22,24,62^. Together, these data indicate *OBi* mice develop extensive pathology in GM with striking similarities to MS patients.

### Depression-like endophenotype and memory deficits

In view of cortical degenerative changes in MS, it may not be surprising that patients exhibit cognitive decline, impaired learning, memory loss and psychopathology including depression and anxiety^14,63^. These comorbidities are prevalent in MS and are important to understand. To determine if *OBi* mice might also exhibit analogous endophenotypes, we performed several behavioral and memory tests.

We first examined the emergence of depression-like endophenotypes in *OBi* mice using tail suspension and forced swim tests at 2, 6 and 12 mo. Baseline testing at 2 mo show *OBi* and littermate controls perform indistinguishably (Fig. 3E,F), as expected because mice receive their first tamoxifen dose following these tests. However, after 4 mo of weekly gavage, *OBi* mice are much more likely to exhibit learned helplessness behavior in both tests. This behavior persists until at least 12 mo of age. Fear extinction testing at 12 mo does not reveal differences between *OBi* mice and littermate controls (Supplementary Fig. S6), suggesting that prefrontal cortex and amygdala circuits are not significantly affected by demyelinating/remyelinating pathology at this age.

We also tested *OBi* mice at several ages for memory deficits using the Win-Shift T-maze and novel object tests (Fig. 3G,H, Supplementary Fig. S6). The *OBi* mice are indistinguishable from controls at 2 and 6 mo; however by 12 mo, these mutants exhibit T-maze memory deficits. The novel object replicates this difference in 12 mo *OBi* mice. Finally, Barnes maze testing at 6 mo does not reveal genotype differences indicative of normal spatial memory and learning at early time points (Supplementary Fig. S6). Together, these data demonstrate that distinct behavioral changes and cognitive deficits are emergent phenotypes with disease progression in *OBi* mice. The depression-like endophenotype precedes hippocampal pathology and memory loss by as much as 6 mo, and likely involves secondary pathology in *OBi* entorhinal cortex in the absence of changes in prefrontal cortex or amygdala.

### Structural protein changes in GM regions of *OBi* mice

**Figure 4:**
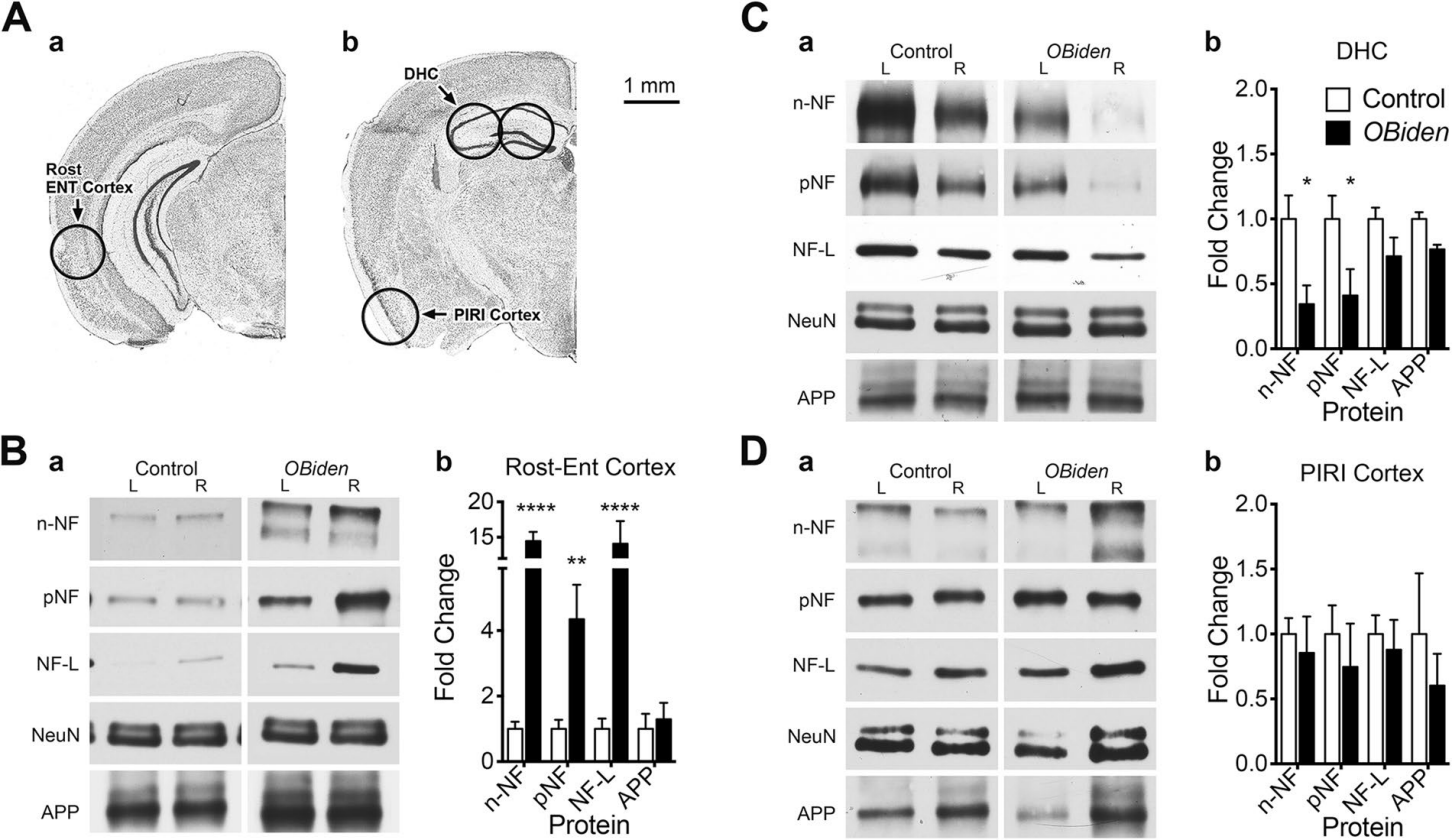
Altered neuron specific axonal transport markers. Western blots for neuron specific axonal transport proteins in left and right GM of 12 mo *OBi* mice. (**A**) Locations of bilateral 1.0 mm diameter brain punches from (a) rostral entorhinal cortex (Rost-ENT Cortex) (b) rostral piriform cortex (PIRI Cortex) and dorsal hippocampus (DHC) in tissue slices (caudal surfaces of slices shown; Nissl Image Credit: Allen Institute). All punches were harvested caudorostrally. (**B**) Representative ENT cortex blots (a) for non-phosphorylated neurofilament (n-NF), phosphorylated NF (pNF), light chain NF (NF-L), amyloid precursor protein (APP) and NeuN as loading control, and (b) quantification showing increases in n-NF, pNF and NF-L in *OBi* mice (ordinary two-way ANOVA, genotype × protein, *p* < 0.002, 4 ≤ *n* ≤ 8). (**C**) Representative DHC blots (a) and quantification (b) showing reduced n-NF and pNF from *OBi* mice (ordinary two-way ANOVA, genotype × protein, *p* < 0.03, 3 ≤ *n* ≤ 4). (**D**) Representative PIRI cortex blots (a) and quantification (b) showing normal protein levels from *OBi* mice (ordinary two-way ANOVA, genotype × protein, *p* > 0.8, 4 ≤ *n* ≤ 6). See also unprocessed western blots in Supplementary Information.

Apparently independent timelines for the emergence of behavior and memory deficits in *OBi* mice suggests some GM regions are more vulnerable to myelin pathology than others. Coupled with normal performance in Barnes maze and spatial learning tests, these data constrain the list of likely brain regions affected, and we focused on pathogenesis in entorhinal and piriform cortices, and dorsal hippocampus. Bilateral tissue punches from normal-appearing GM (NAGM) were harvested (Fig. 4A) from 12 mo *OBi* mice and littermate controls for western blotting of axonally transported structural proteins. Levels of the neuron marker, NeuN, are comparable between left and right hemispheres for controls and *OBi* mice in all three brain regions, indicating there are no major degenerative changes in neurons (Fig. 4B–D). In addition, levels of the synaptic marker, amyloid precursor protein (APP) are unchanged, suggesting circuit connectivity and neuronal cell number are substantially intact.

Although we sampled NAGM in rostral entorhinal cortex from *OBi* mice (Fig. 4B), neurofilament levels are significantly increased. We did not observe large clusters of axonal spheroids in this cortical region (Fig. 3D), suggesting diffusely altered neurofilament isoforms. Such diffuse changes reflect neuron pathology that is common in a number of human diseases and rodent models^64,65^. Levels of n-NF and pNF are decreased in dorsal hippocampus (Fig. 4C), perhaps reflecting axonal loss, and is consistent with memory deficits in these mutants (Fig. 3G,H). Indeed, similar reductions have been observed following acute kainic acid treatment in rats^66^. There are no changes in neurofilament proteins from piriform cortex (Fig. 4D), which discounts the region as a major contributor to behavioral and memory phenotypes in 12 mo *OBi* mice. However, we cannot exclude the possibility of subsequent damage with disease progression.

### Shorter axon initial segments in deep layer neurons from rostral entorhinal cortex

Early onset of the depression-like endophenotype in 6 mo *OBi* mice, coupled with robust neurofilament changes, suggest significant sensitivity of entorhinal cortex to presumptive cycles of demyelination/remyelination compared to proximate regions like piriform cortex. The function and output of entorhinal cortex is determined to a large extent by the balance of excitatory and inhibitory signals on pyramidal neurons and previous studies implicate axon initial segment (AIS) changes as a source of cortical dysfunction^67,68^. We observed no obvious abnormalities in a pilot experiment with *OBi* layer II/III pyramidal cells; thus, we focused on the AIS of layer 5/6 neurons.

**Figure 5:**
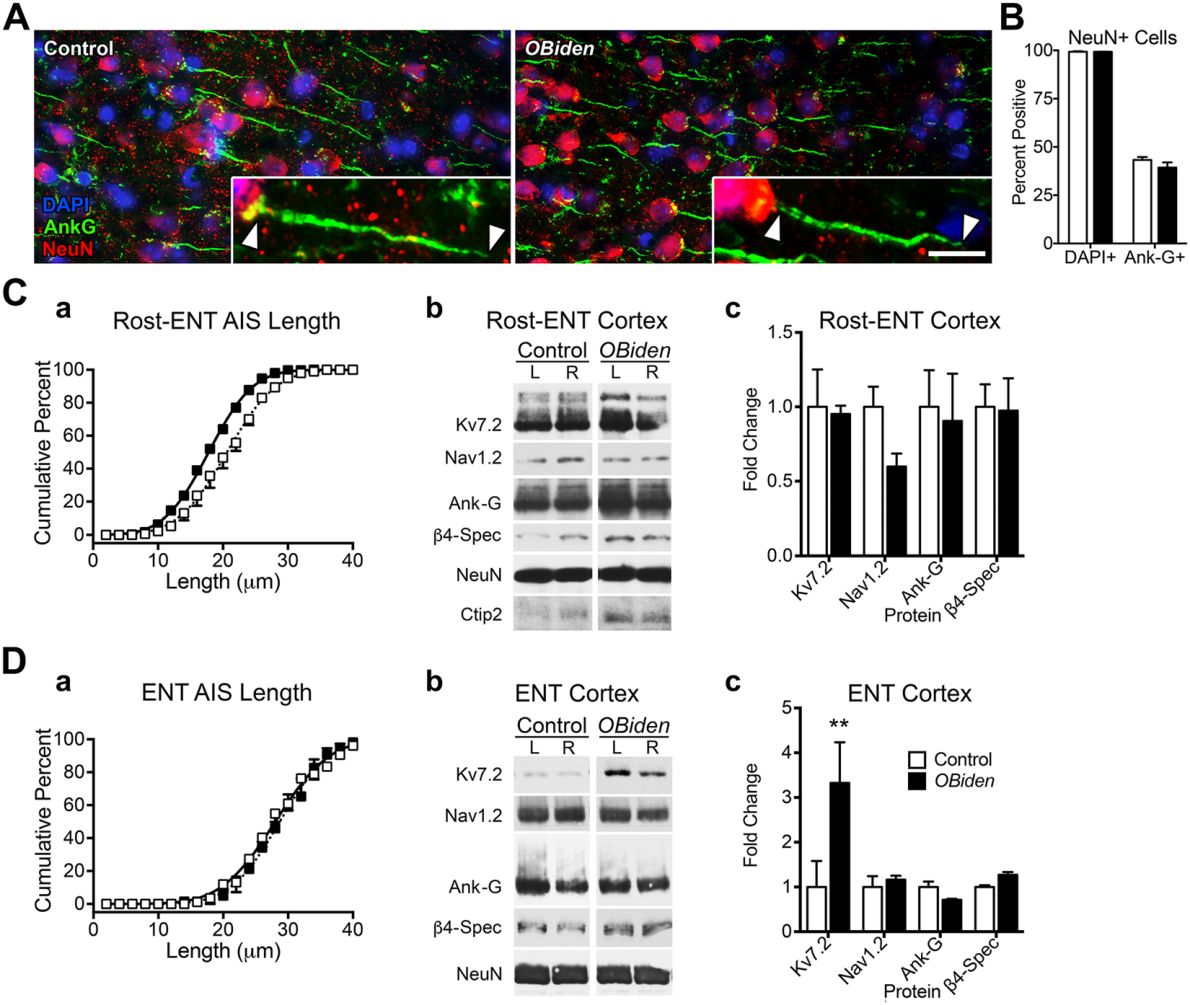
Secondary consequences of demyelination/remyelination pathology on axon initial segment (AIS) length in 12 mo entorhinal cortex. (**A**) Immunofluorescence labeling of control and *OBi* cortical layer 5 neurons for Ank-G (green) and NeuN (red). (**B**) Proportions of NeuN^+^ cell bodies, both DAPI^+^ and Ank-G^+^, are similar between genotypes (ordinary two-way ANOVA, genotype × cell marker, *p* > 0.2, *n* = 3). (**C**) Average AIS length in *OBi* rostral entorhinal (Rost-ENT) cortex (a) is significantly shorter than controls (extra sum-of-squares F test for comparison of Gaussian fits, 17.8 ± 0.1 μm versus 20.6 ± 0.2 μm; *p* < 0.0001, *n* = 3). (b) Representative western blots from rostral entorhinal cortex (Rost-ENT) for AIS functional and structural proteins and neuronal cell body markers, NeuN and Ctip2, a specific marker for post-mitotic layer 5 neurons. (c) Quantification shows no differences between genotypes (two-way RM-ANOVA, genotype × AIS protein, *p* > 0.3, *n* = 4). (**D**) AIS length in layer 5 entorhinal (ENT) cortex (a) is similar between genotypes (extra sum-of-squares F test for comparison of Gaussian fits, 28.7 ± 0.2 μm versus 28.0 ± 0.2 μm; *p* = 0.011, *n* = 3). (b) Representative western blots from left and right ENT cortex for AIS proteins and (c) quantification shows significantly increased Kv7.2 levels (two-way RM-ANOVA, genotype × AIS protein, *p* < 0.002, *n* = 4). Abbreviations: Ank-G, ankyrin-G; β4-Spec, β4-spectrin; NeuN as loading control. Scale bar in A, 25 μm; inset, 8.3 μm. See unprocessed western blots in Supplementary Information.

Ankyrin-G (Ank-G) is a major scaffolding protein of the AIS and defines this domain^69^. NeuN^+^ pyramidal neurons in rostral entorhinal cortex are strongly labeled at the juxtaperikaryon by anti-Ank-G antibodies and essentially all DAPI^+^ nuclei are NeuN^+^ neurons (Fig. 5A,B). Full-length AIS profiles are associated with 40% of these cells in both genotypes, which is a function of coronal plane and section thickness (10 μm) and indicates the overall preservation of cortical architecture in *OBi* mice.

Lengths of the AIS from 12 mo wild type mice are normally distributed about a mean of 20.6 ± 0.2 μm (Fig. 5Ca). This distribution is left-shifted bilaterally in *OBi* mice to 17.8 ± 0.1 μm, suggesting pathology. Despite this shortening, levels of scaffolding proteins and the Nav1.2 and Kv7.2 ion channels are comparable to controls (*p* > 0.37), suggesting at most modest increases in ion channel density (Fig. 5Cb,c). Further, levels of NeuN and the Ctip2 specific marker for post-mitotic layer 5 neurons are normal and indicate that cell loss is not significant. Studies in other model systems indicate that AIS distance from the soma also can be modulated by pathology e.g.^70^, but we observe only shortening in *OBi* mice. Together, our data suggest that AIS pathology in deep layer principal neurons from 12 mo *OBi* mice is limited to structural changes.

### Altered Kv7.2 channel levels in entorhinal cortex and dorsal hippocampus

We also performed a morphometric analysis more caudally in entorhinal cortex because of the known functional heterogeneity within this structure^71^. Average AIS lengths are greater than in rostral entorhinal cortex (Fig. 5Da) and there is little difference between *OBi* and controls. Western blots of AIS scaffolding proteins are also normal, but Kv7.2 channels are increased 3–fold (Fig. 5Db,c), suggesting functional changes to principal neurons. The kinetics of Kv7.2 channels indicate they modulate overall AIS excitability rather than shaping the repolarizing phase of action potentials (APs)^72–75^; thus, increasing AIS channel density likely suppresses output signaling to hippocampus, particularly from medial entorhinal cortex^76^.

**Figure 6:**
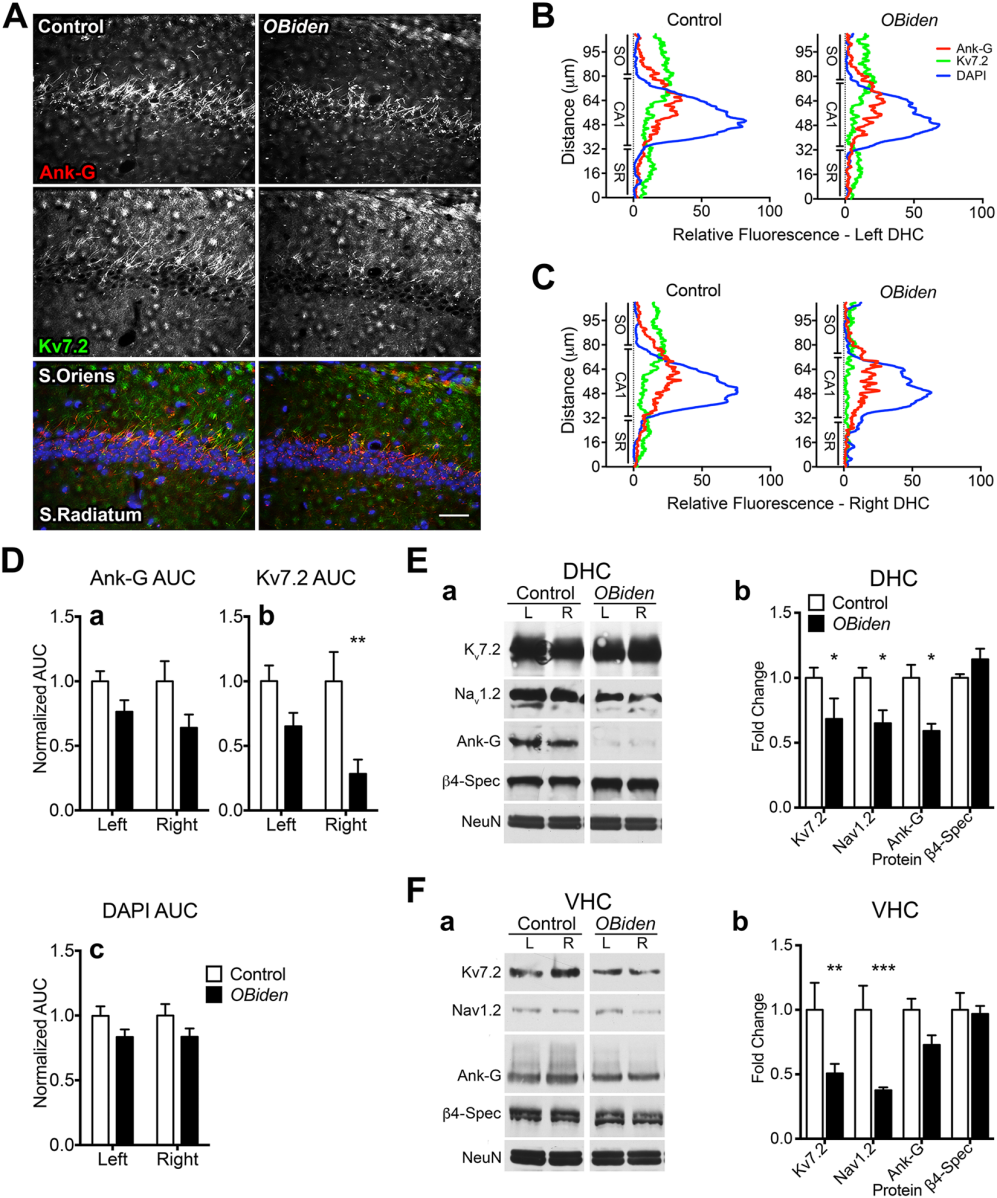
Ion channel disruptions in AIS of hippocampal CA1 neurons. (**A**) Immunofluorescence labeling for Ank-G (red) and Kv7.2 (green) in the CA1 from control and *OBi* dorsal hippocampus (DHC). DAPI (blue) shows nuclei. (**B**,**C**) Fluorescence intensity plots for each marker in (**A**) across the left (**B**) and right (**C**) DHC CA1. Measurements are orthogonal to CA1 along a 107 μm trace from stratum radiatum (SR) to stratum oriens (SO) (*n* = 5 mice, 3 slides/mouse). (**D**) Areas under the curves (AUC) in (**B**) and (**C**) for (a) Ank-G, (b) Kv7.2 and (c) DAPI. Kv7.2 intensity in the right DHC CA1 region from *OBi* mice is reduced compared to controls (two-way RM-ANOVA, genotype × hemisphere, *p* < 0.008, *n* = 5). (**E**) Representative western blots of DHC punches from control and *OBi* mice for AIS proteins (a) and quantification (b) shows significant reductions in Ank-G, Kv7.2 and Nav1.2 in *OBi* mice (two-way RM-ANOVA, genotype × AIS protein, *p* < 0.05, *n* = 4). (**F**) Representative western blots of left and right ventral hippocampus (VHC) from control and *OBi* mice for AIS proteins (a) and quantification (b) shows reduced Kv7.2 and Nav1.2 in *OBi* mice (two-way RM-ANOVA, genotype × AIS protein, *p* < 0.01, *n* = 4). Scale bar in A, 50 μm. Abbreviations: β4-Spec, β4-spectrin; Ank-G, ankyrin-G; NeuN as loading control. See unprocessed western blots in Supplementary Information.

Ankyrin-G staining in hippocampal CA1 suggests the AIS architecture of axons entering the stratum oriens is disorganized in *OBi* mice (Fig. 6A). Nevertheless, bilateral fluorescence intensity-distance plots quantified from digitized traces between stratum radiatum and stratum oriens (Fig. 6B–D) indicate normal Ank-G levels in *OBi* mice. While Kv7.2 staining in left CA1 is normal, contralateral Kv7.2 channel staining is significantly reduced. This raises the possibility of asymmetric hippocampal vulnerability to disease, which has been previously observed but is typically associated with behavioral or synaptic asymmetry^77–79^. The DAPI labeling in CA1 is comparable between genotypes and rules out major neuron losses in this layer.

Western blots of bilateral DHC tissue punches (Fig. 6E) show reduced Kv7.2, Nav1.2 and Ank-G levels. Importantly, the punches include CA2, CA3 and dentate gyrus; thus, changes may not be limited to the output neurons but may arise from AIS pathology throughout DHC. Normal NeuN^+^ granular layer density suggests neuron loss does not account for decreases in the other proteins. Western blots of VHC tissue punches also show reductions in Kv7.2 and Nav1.2 compared to controls (Fig. 6F), while Ank-G, β4-spectrin are normal. We are unable to examine AIS lengths in the VHC in a manner analogous to Fig. 6A–D because neurons around the granular layer are oriented obliquely in sections.

Together, entorhinal and hippocampal changes in 12 mo *OBi* mice suggest progressive pathophysiological processes involving at least the output activity of incumbent principal neurons. Presumptive demyelination/remyelination cycles induced in *OBi* mice may initially impact entorhinal cortex and subsequently spread to hippocampus. However, we cannot rule out the possibility that hippocampal principal neurons are also affected early by demyelination/remyelination but are more resistant to damage than in cortex.

### Comparable cortical changes in *OBi* mice and MS autopsy tissue

**Figure 7:**
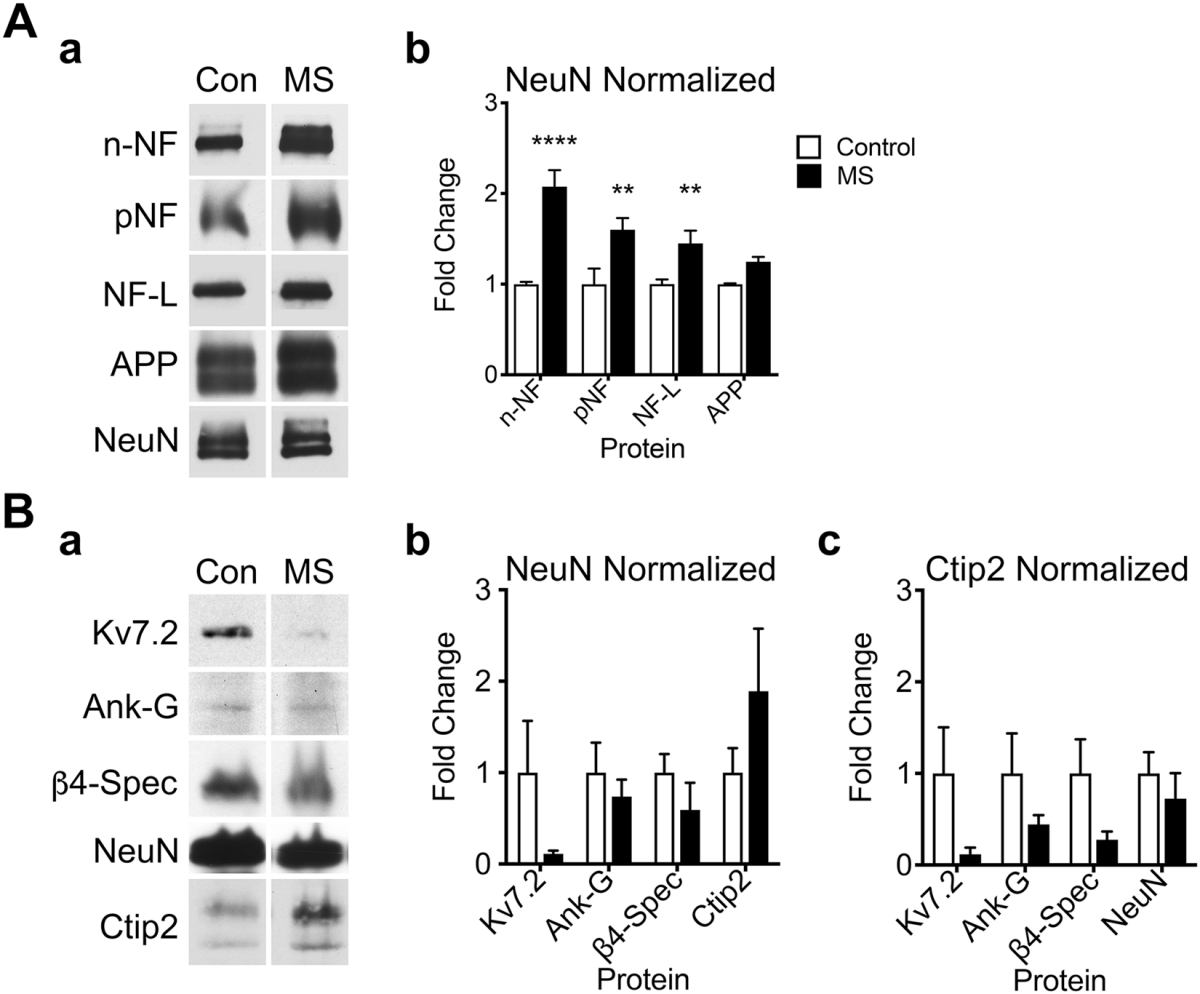
Neurofilament and AIS pathology in frontal cortex of MS patients. Western blot changes in NAGM for structural and AIS proteins in human frontal cortex. (**A**) Representative blots (a) of NAGM from non-neurological control (Con) and multiple sclerosis (MS) patients for non-phosphorylated neurofilament (n-NF), phosphorylated NF (pNF), light chain NF (NF-L), amyloid precursor protein (APP) and NeuN as loading control. (b) Quantification of blots and two-way ANOVA (disease x protein) indicates statistical significance between the control and MS groups (*F*_(1,23)_ = 64.3, *p* < 0.0001). Holm-Sidak posthoc tests show increases in n-NF (*p* < 0.0001, *n* = 4), pNF (*p* < 0.002, *n* = 3 or 4) and NF-L (*p* < 0.008, *n* = 4) in MS NAGM compared to controls. (**B**) Representative blots (a) of AIS proteins for Kv7.2, Ank-G, β4-spectrin (β4-Spec), NeuN and Ctip2 as loading controls. (b) Quantification of western blots normalized to NeuN shows a large variance in the Ctip2 marker of layer 5/6 pyramidal cells. These data suggest variability in tissue sampling that is not adequately controlled after normalizing to the NeuN pan-neuronal marker. (c) Quantification of blots and normalizing to the Ctip2 marker for layer 5/6 pyramidal neurons controls sampling variability. Overall two-way ANOVA (disease x protein) indicates statistical significance between the control and MS groups (*F*_(1,24)_ = 8.05, *p* = 0.009); however, Holm-Sidak posthoc tests do not reveal changes in MS for any individual AIS protein (*p* > 0.18). See also Supplementary Fig. S7, and unprocessed western blots in Supplementary Information.

In light of the pathology in 12 mo *OBi* mice, we investigated molecular parallels of disease with MS, including axonal transport defects and disruption of the AIS in deep layer cortical principal neurons. We obtained frontal cortex autopsy samples from PPMS and SPMS patients and non-neurodegenerative controls (Supplementary Fig. S7). This brain region is roughly analogous to mouse entorhinal cortex in structure and function^80^. Western blots of punches from normal-appearing cortical layers 5/6 show 2–fold increases in neurofilament proteins from the MS samples (Fig. 7A). Comparable levels of APP between patients and controls indicate we did not harvest tissue punches with significant GM lesions.

Quantification of western blots for AIS proteins normalized to NeuN shows no statistical differences between MS patients and controls (Fig. 7Bb), although the high variability of Ctip2 levels in the MS punches suggests variable sampling of deep cortical layers (e.g. unseen cortical folds near sulci causing inclusion of Ctip2-negative superficial cortical layers). To compensate for such artifacts, we normalized blots to Ctip2 (Fig. 7Bc). This transcription factor is a marker for mature pyramidal cells, which comprise the bulk of myelinated neurons in layers 5/6, and are the cells most likely damaged by demyelination/remyelination and AIS changes. Indeed, this alternative normalization reduces the variance compared to NeuN normalization. A two-way ANOVA indicates an overall statistical difference between normal and disease states, and average levels of the AIS proteins appear lower in the MS samples. Nevertheless, posthoc tests show no statistical differences between the groups, which likely reflects the variability between control samples.

### AIS length modulates back AP amplitudes in simulations

To develop a mechanistic understanding of AIS changes in *OBi* and MS cortices, we simulated deep layer myelinated principal neurons in silico^51^. In rodents, these cells project both locally to layers II/III, presumably through unmyelinated collaterals, and subcortically to hippocampus, basal ganglia and insular cortex^76^. The simulated myelinated axon is sufficiently long to project through the underlying WM to distant targets, and includes asymmetric AIS sodium channel densities to generate APs and back-propagating APs (bAPs)^81^. Simulated current clamps at the soma were used to generate single bAPs and APs approximately 0.1 pA above the depolarization threshold. The AIS length was varied from 3–60 μm (wild type AIS length = 20.3 μm and *OBi* AIS length = 16.8 μm) and myelin thickness was changed from 0 (demyelinated, *g*-ratio = 1) to 10 (wild type myelin *g*-ratio = 0.8) wraps.

**Figure 8:**
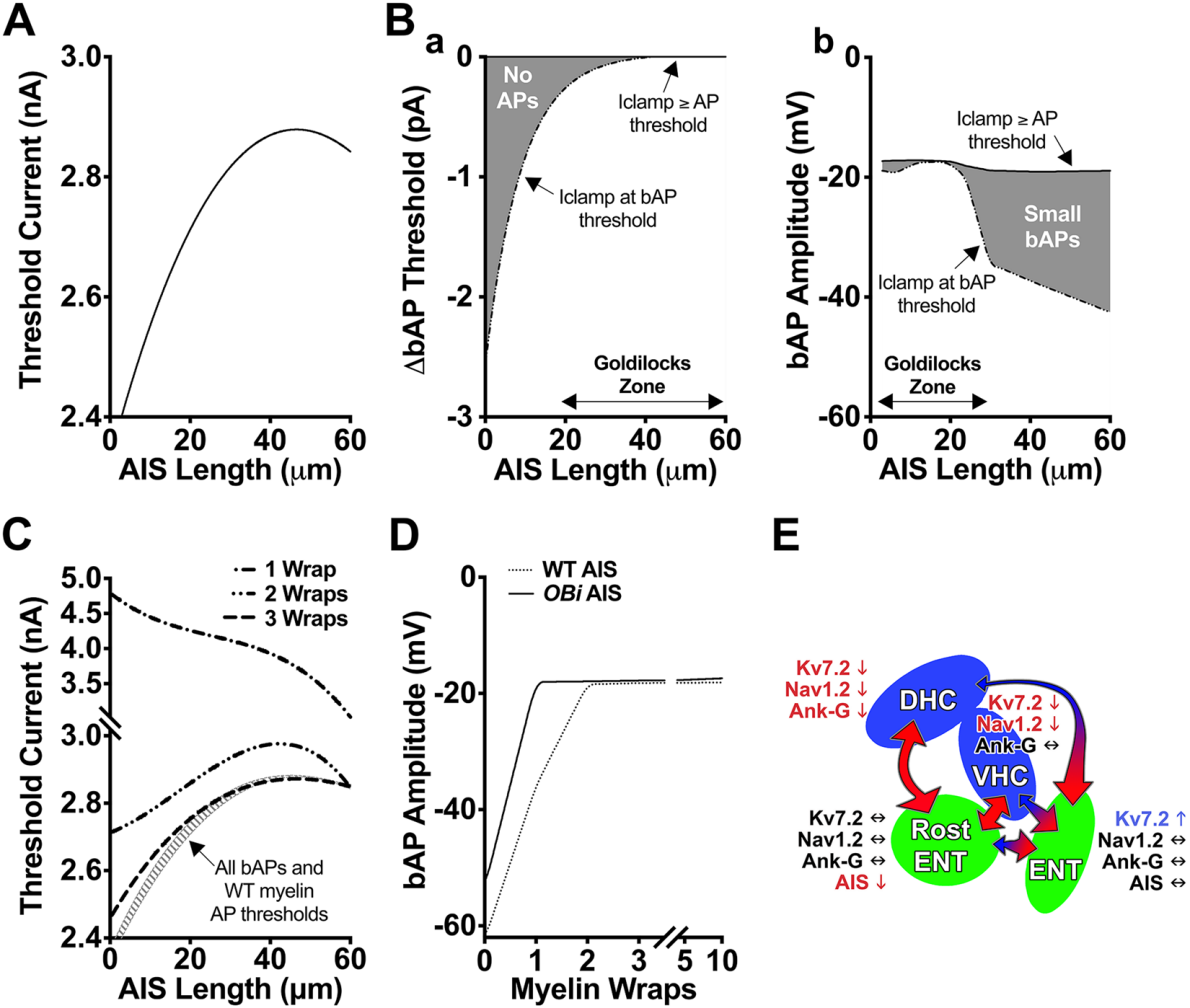
Neuron simulations suggest AIS length changes compensate for proximal demyelination. Current clamp simulations of Rost-ENT cortex neurons. (**A**) Threshold current is proportional to AIS length in a wild type cell. (**B**) Analytical detection (gray zone) of (a) uncoupling between bAP and AP threshold currents during extreme AIS shortening and (b) diminished bAP amplitudes during extreme AIS lengthening. Mid-range AIS lengths appear stable (Goldilocks zones). (**C**) Changes to bAP and AP threshold currents caused by proximal demyelinating and early remyelinating lesions as functions of AIS length. The bAP threshold currents (gray striped region) are refractory to myelin pathology, while AP threshold currents (dashed lines) are markedly sensitive to demyelination and early remyelination. With four or more lamellae around axons, bAP and AP threshold currents are similar. (**D**) Comparisons of bAP amplitudes generated by wild type (WT) and *OBi* AIS lengths (Fig. 5C) as a function of the number of myelin wraps. The bAP thresholds are invariant with myelin thickness, while AP thresholds are elevated for thin myelin. AIS shortening in *OBi* mice restores bAP amplitudes to normalcy with the first myelin wrap. (**E**) Schematic summarizing our findings and the major conserved neural circuits between three brain regions involved in behavior, memory and learning: entorhinal cortex (Rost-ENT and ENT), dorsal hippocampus (DHC) and ventral hippocampus (VHC). Primary pathology in oligodendrocytes leads to demyelination/remyelination in 12 mo *OBi* mice and may cause degenerative changes in the corticohippocampal loop circuit. Large arrows (red) signify likely increases in synaptic tone, while small arrows reflect likely decreases associated with hypomyelination/demyelination or altered components in the AIS (blue). Overall, the data suggest increased input (higher synaptic tone) into entorhinal cortex and decreased input (lower tone) into hippocampus. We hypothesize that episodic induction of pathology in *OBi* mice increasingly damages WM, disrupts cognitive networks and affects higher-order behavior, memory and learning. These changes are reminiscent of behavioral symptoms in MS patients. See also Supplementary Fig. S8.

Current thresholds for AP generation as a function of AIS length approximate a polynomial function (Fig. 8A) that for the most part is directly proportional to AIS length and indirectly proportional to neuron activity^82,83^. This dependence of neuron activity on AIS length regulates sensitivity to changes in dendritic input and homeostasis, and the 400 pA current threshold range of the curve is sufficiently large to be of physiologic relevance. Such plasticity may come at a price (Fig. 8B), because theoretical vulnerabilities in AP or bAP generation emerge at the extrema of AIS length.

At wild type AIS lengths (Fig. 8B, Goldilocks zone), bAP and AP threshold currents are virtually identical and recapitulate the tight coupling of these events in physiologic neurons. However, an analytical analysis suggests they are separable during AIS shortening, whereby the threshold magnitudes for generating bAPs are several pA lower than for APs (Fig. 8Ba). At the opposite extreme, AIS lengthening maintains bAP/AP coupling but diminishes bAP amplitudes (Fig. 8Bb). Whether or not physiologic neurons manifest such vulnerabilities is currently unclear, but simulated bAPs at all AIS lengths are coincident with full amplitude somatic depolarization and are distinct from spikelets^84^.

Eliminating the first 20 myelin internodes in our model simulates a proximal demyelinating lesion (Type I^85^) at the GM-WM border of external capsule, which causes conduction block. In contrast bAPs are generated, albeit with diminished amplitude, and the threshold current for generation is largely unchanged (Supplementary Fig. S8). Conduction is restored in simulations with at least one myelin wrap (hypomyelinated wild type axon); however, the AP threshold current is dramatically increased compared to wild type, and is uncoupled from bAP thresholds, particularly at short AIS lengths (Fig. 8C). Additional myelin wraps reduce AP threshold currents at all AIS lengths, and by three lamellae the these currents approximate wild type. In the case of distal demyelination beyond the proximal 20 internodes, which simulates WM pathology in external capsule or corpus callosum, threshold currents for bAPs and APs are normal, but conduction block occurs across the demyelinated segment as expected.

Under presumed demyelination/remyelination conditions in *OBi* mice (Supplementary Fig. S3), the AIS of layer 5 neurons in rostral entorhinal cortex is shortened approximately 16% compared to controls (Fig. 5C). Simulations demonstrate that the bAP amplitude growth curve for demyelinated neurons with wild type AIS length increases linearly with each myelin wrap and plateaus from 2–10 wraps (Fig. 8D). However, shortening the AIS to that observed in *OBi* rostral entorhinal cortex left-shifts the growth curve and the threshold current plateau is achieved at one myelin wrap. Thus, a rapid reduction in AIS length may be functionally important for demyelinated neurons to maximize bAP amplitudes and minimize the threshold current for AP generation at the earliest stages of remyelination.

Simulations to examine altered Ank-G, Nav1.2 and Kv7.2 densities in the AIS of hippocampal CA1 neurons (Fig. 6) are not currently feasible because ion channel distributions for these cells are poorly characterized, and tissue architecture precludes a clear understanding of the structural and functional changes in *OBi* mice. Observed increases in Kv7.2 from deep layer neurons of entorhinal cortex (Fig. 5D) do not substantially alter model properties in our simulations and we gain no insight into mechanisms of action. We presume the changes reflect adaptations to altered somatodendritic activity stemming from the indirect consequences of demyelination/ remyelination or neuron loss in the corticohippocampal loop. Such changes are implicated in *OBi* mice by the behavioral changes we observe (Fig. 3).

## Discussion

We and others previously demonstrated that metabolic stress and induction of the UPR are associated with the pathophysiology of MS and may provoke secondary innate and adaptive immune responses in mouse CNS^5,9,23,86^. Further, clinical studies link MS to the expression of metabolic stress-inducing mutant isoforms of PLP1, either through misdiagnosis of Pelizaeus-Merzbacher disease as MS, or by demonstrating somatic RNA editing of *PLP1* mRNA (and the encoded mutant protein) in demyelinating lesions from MS patients^7,31,87^. Thus, our long term goals for *OBi* mice are to determine the extent to which a primary disseminated metabolic stress etiology in oligodendrocytes induces progressive WM damage (including secondary GM and innate/adaptive immune involvement) and phenocopies the pathophysiology of MS.

Primary oligodendrocyte impairment or death as a model of MS-like pathology may provide significant insight into downstream consequences of WM damage. While some animal models induce global oligodendrocyte pathology during development (e.g. the leukodystrophies^88^), or promote cell death using exotic toxins^25^, we employ circumscribed endogenous metabolic stress in *OBi* mice beginning in adulthood after the CNS is fully myelinated. Tamoxifen gavage beginning at 2 mo triggers a primary oligodendrogliopathy characterized by metabolic stress and UPR activation in mature *OBi* oligodendrocytes. We use missense mutant *Plp1* gene products to drive disease; however, accumulation in the secretory pathway of any abundant endogenous or exogenous protein will similarly trigger an oligodendrogliopathy^89–91^. Thus, mutant PLP1 expression in *OBi* mice is arguably a non-specific adult-onset metabolic stress paradigm, not a mouse model of a specific disease associated with the *PLP1* gene.

Coincidentally, a recent multifactorial analysis in MS patients by Qendro and colleagues^7^ has identified apparent post-transcriptional mutations in PLP1 from active lesions. These mutations apparently involve aberrant RNA editing of *PLP1* transcripts (but not other major myelin transcripts) leading to missense mutations in the protein revealed by proteomics. Expression of mutant PLP1 isoforms can certainly induce metabolic stress and the UPR in oligodendrocytes, which has been demonstrated in MS autopsy samples^5,9,92^. The Qendro^7^ study leaves unanswered several questions – what is the mechanism of RNA editing? what pathological processes might dysregulate the homeostatic constraints on this editing? – but if corroborated, implicates the *OBi* mouse as an explicit model of MS pathobiology.

Progressive disease in *OBi* mice includes disseminated hypomyelination, unilateral focal WM lesions, innate immune activation, apparent glial scarring, cortical axon thickening, spheroids, transections, neurofilament changes and abnormal interhemispheric EEGs. These findings are emblematic of MS^13,20^, several aspects of which we confirm in MS autopsy tissue. Behavioral changes in *OBi* mice include rare intermittent motor deficits, a depression-like endophenotype manifested early and eventually memory deficits. Such abnormalities suggest pyramidal cell pathology in the corticohippocampal pathway, which we confirm directly using immunofluorescence microscopy and western blotting.

Steady-state levels of AIS structural proteins/ion channels and neurofilaments are perturbed in entorhinal cortex and hippocampal CA1 from *OBi* mice, and some of these features are shared with frontal cortex from MS patients. Although the AIS length changes are unlikely to be associated with demyelination per se, or other previously reported causes such as physical injury or ischemia, currently we cannot exclude effects associated with microglial activation^93^. Irrespective of the cause, these perisomatodendritic molecular changes are of particular interest because they suggest mechanistic connections between oligodendrocyte damage and cognitive deficits. On the other hand, the neuronal cytoskeletal changes that we observe reflect more generalized pathology, and have been observed in many mouse models of neurodegeneration or disrupted anterograde/retrograde axonal transport^56,94–96^.

Our functional data also indirectly link primary oligodendrocyte damage to secondary cognitive decline. Histological changes to major WM tracts in *OBi* mice are apparent within a few months after disease induction, but functional data from auditory evoked potentials and interhemispheric EEGs at rest indicate that pathway integrity is largely unperturbed until almost 12 mo. Even at this stage, pathology is subtle and not detected by techniques such as diffusion tensor imaging. Although reduced theta band coherence can be used to interrogate memory disturbances^97^, the common-vertex electrode montage used herein only permits evaluation of major myelinated commissures. This limitation notwithstanding, our data strongly support WM dysfunction from several view points, and it is unlikely that major tracts – corpus callosum, internal and external capsule, stria medullaris – are compromised in isolation. Comparable damage to other regional tracts such as the cingulum bundle and fornix are expected to impact myelinated connections between cortex and hippocampus and contribute to learning and memory deficits.

Physiologically-normal myelin turnover continues throughout life in mammals, and nascent synthesis is important for new learning and memory^98,99^. Thus, neurons are likely capable of adapting to, if not promoting, myelin remodeling beyond development (i.e. myelinogenesis). If so, it is unclear why repetitive demyelination used in the current study should exact the significant neuronal damage that we observe, particularly in the absence of adaptive immune responses, which appears to be the case in *OBi* cortex and hippocampus. Nonetheless, the structural and functional consequences of primary and secondary pathology in *OBi* mice are apparent and may be useful for developing a working model with which the link between oligodendrogliopathy and cognitive deficits can be explored and possibly established.

At the cellular level, our simulations indicate that proximal myelin damage/demyelination diminishes or eliminates AP generation. In contrast, bAP generation is uncoupled from APs and persists in these neurons, albeit with amplitudes that are reduced by several factors. This bAP/AP uncoupling may contribute to pathology. Thus, bAPs are known to arise from antidromic depolarization of Nav1.2 channels the proximal AIS/soma, and once refractory, these channels remain inactive until restoration of the resting membrane potential. Generation of bAPs at their normal current thresholds (hatched region, Fig. 8C) can significantly impede summation at the AIS, so that the elevated threshold currents necessary for AP generation (dashed curves, Fig. 8C) may not be readily achieved. The resulting reduction in AP generation would reduce downstream signaling, and drive compensatory mechanisms (excitatory or disinhibitory) that increase feedback activity, potentially to the point of excitotoxicity^100^.

Simulations also suggest a potential link between small bAP amplitudes and pathophysiology. The functions of bAPs are incompletely understood, but may be important for synaptic plasticity or synaptic feedback, and several other roles have been hypothesized^101^. In this vein, we suggest a function in cell survival. Thus, persistent bAP generation in the somatodendritic compartment of demyelinated neurons may be instrumental for conveying proximal axon integrity and inducing cell survival pathways from presynaptic neurons. Maintenance of such pathways would promote homeostasis, rather than the demyelinated cell yielding to pathways that trigger degeneration. In addition, voltage-sensitive receptors localized to distal dendrites may respond to changes in bAP amplitudes and modulate expression of AIS component genes, thereby controlling AIS length. Indeed, metabolic feedback by neuromodulators and cyclic AMP signaling is known to induce transcription of target genes in response to long term potentiation at post synaptic membranes^102^.

More broadly at the level of the corticohippocampal loop circuit, AIS changes that we and others observe^103,104^ suggest neurons can compensate for focal demyelination or generalized hypomyelination in different ways (Fig. 8E). For example, demyelination of cortical pyramidal cells likely decreases downstream signaling from entorhinal cortex to hippocampus. The CA1 neurons may respond by reducing several AIS markers and AIS length, which should increase sensitivity and output (tone). Enhanced somatodendritic activity from hippocampus and rostral entorhinal cortex to entorhinal cortex presumably becomes supranormal, causing an increase in Kv7.2 levels and a reduction in sensitivity and output (tone). In turn, this response would further diminish cortical output and exacerbate CA1 neuron compensation, leading to a positive feedback loop and circuit dysfunction with intermittent or long term consequences for behavior, memory and learning.

The intriguing parallels between *OBi* mice and MS, from innate immune activation, glial scarring and focal/disseminated hypomyelination (Fig. 2, Supplementary Fig. S3), to axonal swellings/transections (Fig. 3) and neurofilament abnormalities in NAGM (Fig. 4) suggest that primary oligodendrocyte metabolic stress is sufficient to account for multiple aspects of pathology observed in patients. Future studies in aging *OBi* mice will undoubtedly reveal whether-or-not adaptive immune responses ensue and whether these mutants are abnormally susceptible to generalized immune stimulatory signals or specific molecules that trigger autoimmune activation. In contrast, if these responses do not arise, the *OBi* phenotype may be more analogous to primary progressive MS.

Perhaps the most interesting findings of the current work include the demonstration of mechanistic links between primary oligodendrocyte damage and neuron dysfunction in *OBi* mice, which parallel degenerative changes in MS tissue. Technical difficulties have thwarted our efforts to label MS cortex with current anti-Ank-G antibodies so we cannot extend our analyses to similar levels as in Figs 5 and 6. Although western blots from MS tissue suggest important insight into cortical pathology, they lack the single cell specificity of morphometric analyses for a more detailed understanding. Nevertheless, the spatiotemporal coincidence of progressive pathology and behavioral deficits in *OBi* mice underscores our expectations for similar changes in patients. Indeed, such broadly analogous pathophysiology in both species suggests that our novel oligodendrogliopathy model is relevant not only to MS, but also to the pursuit of disease modifying therapies and, optimistically, defining etiology in at least some cases.

## Electronic supplementary material

Supplementary Information

## Data Availability

All relevant data will be made available by the corresponding author upon reasonable request. The MATLAB scripts generated for EEG analyses will be made available upon reasonable request.
