# Supplementary material for: Corticohippocampal Dysfunction In The OBiden Mouse Model Of Primary Oligodendrogliopathy
Source: Sci Rep. 2018 Oct 31;8:16116. doi: 10.1038/s41598-018-34414-7 (PMC6208344; doi:10.1038/s41598-018-34414-7)

Supplementary Information

Corticohippocampal Dysfunction In The *OBiden* Model Of Primary Oligodendrogliopathy

Daniel Z. Radecki, Elizabeth L. Johnson, Ashley K. Brown, Nicholas T. Meshkin, Shane A. Perrine and Alexander Gow

Genotyping and primers

Mice were genotyped at 7 days if age from tail biopsy DNA. Tail tips were digested overnight at 55°C in 100µl DirectPCR (Tail) buffer (Viagen Biotech Inc., Los Angeles, CA) containing 0.5mg/ml of proteinase K (Promega Corp., Madison, WI) according to the manufacturer protocol. The following primers were used for genotyping from tail DNA. For the $Plp1^{i.msd}$ allele; 3 x PCR primers were used to distinguish wild type (493bp product) from $Plp1^{i.msd}$ (428bp) alleles: sense – GAG GTT GAC ACT TAG GGA AGT G; antisense 5' – TTT CCC ACC AGA GAC TAC CGA G; antisense – GCA GCC TCT GTT CCA CAT ACA C. In addition, the first 2 primers above were used in PCRs to determine the cre-mediated recombination efficiency of DNA purified from cryostat tissue sections of *OBi* and littermate controls (see below). Thus, the wild type $Plp1^{OeX}$ allele yields a 481bp product, while the product from the cre-recombined $Plp1^{i.msd}$ allele is 545bp. For the *McreG* allele; 2 x PCR primers (290bp product): sense 5' – GAG TAC GTG CTC GCT CGA TGC; antisense 5' – CTC CCA CCG TCA GTA CGT GAG AT. For the Plp^{OeX} allele; 2 x PCR primers (400bp product): sense 5' – CAG GTG TTG GAG TCT GAT CTA CAC AAG; antisense 5' – GCA TAA TAC GAC TCA CTA TAG GGA TC [1].

Estimating Cre-mediated recombination efficiency in CNS tissue

Because of the stochastic nature of inducing cre-mediated recombination of the $Plp1^{i.msd}$ allele in *OBi* mice, the efficiency of recombination from mouse-to-mouse may be quite variable because of potentially myriad variables, including genetic diversity in the breeding stocks (the mice are not inbred), subtle variations in CNS vasculature and the efficiency of absorbing the tamoxifen gavage from the gut. To estimate this variability, we raised *OBi* and littermate control mice to 8mo of age in the absence of tamoxifen, and then gavaged them (150mg/kg tamoxifen) on 2 consecutive days. The mice were injected i.p. with 50mg/kg BrdU in sterile saline, twice 3 hr apart, then 4% paraformaldehyde perfused for 10µm cryostat parasagittal sectioning of cerebellum/brainstem.

For PCR detection of recombination efficiency, 2 cryostat sections/mouse were scraped from glass slides with a clean razor blade into Ependorf tubes. The tissue samples were digested overnight in 50µl DirectPCR (Tail) buffer (Viagen) containing 0.5mg/ml of proteinase K (Promega) as for genotyping. Samples were electrophoresed on 2% agarose gels and the ethidium bromide stained gel was digitized and the bands quantified using Image J. Scans of the bands corresponding to the wild type and recombined *Plp1* alleles were examined to ensure peak flattening was absent (i.e. intensity saturation). Areas under the peaks were adjusted for DNA band size (wild type=481bp, recombined=545bp), allele copy number ($Plp1^{OeX}$=2 copies/haploid genome; we disagree with the Southern blot interpretation by Readhead and colleagues [1]; $Plp1^{i.msd}$=1 copy/haploid genome) and the ratio of total cells:mature oligodendrocytes in optic nerve (1:1) to compute the Recombination Frequency/oligodendrocyte (RF_{OL}):

$$RF_{OL} = AUC^{i.msd} / AUC^{OeX} * 481/545 * 2/1 * 2$$

Behavioral testing

Inverted screen: modified Kondziela's test [2] using a 40cm x 40cm x 10cm deep frame constructed from 5cm x 2.5cm wood with a 19 gauge wire mesh (1.2cm x 1.2cm holes). Mice are placed in the center of the mesh inside the frame so they cannot escape and the frame is then slowly rotated (2–5s) 180° so the mice hang upside down approximately 40cm above a padded gray plastic tub. The upper surface of the mesh is painted with a 6cm x 6cm grid for quantifying lateral movements of the mice. Mice that immediately fall once inverted are retested but two immediate falls yields a zero score. The test runs for 2min or until the mice fall. The time based scoring scale is (1–10): 1, 1–10s; 2, 11–20s; 3, 21–30s; up to 9, 81–90s and finally 10, 91–120s. A movement based assessment, defined as the number of times both front paws cross a painted line is divided by 3, is added to the time score to yield an overall score of 0~20.

Mice are tested weekly (8–52wks) just prior to gavage, and the median score for each mouse is used to normalize their weekly scores to approximate unity (0.7–1.3). Motor deficits are detected as a 3-fold drop in score (e.g. 1 to ≤0.33), with the prior and subsequent 2 scores (i.e. a 5wk assessment window) approximating unity. This yields a characteristic "V" shape signature on score-time plots. Signatures arising from continuously fluctuating weekly scores are discounted.

Novel object recognition: open field arena (80cm x 80cm) with digital video recording for analysis using Ethovision tracking software (version 8.5, Noldus, Leesburg, VA). The test comprises 3 sessions in one day and cleaning the arena with 70% ethanol between sessions [3]. First, mice are habituated to the empty arena for 10min (Supplementary Fig. S6C) and returned to the home cage for 20min. In session 2, mice explore the arena for 10min, after placement of two identical objects (e.g. colorful plastic or glass toys; cleanable) in opposite corners, and are returned to the home cage for 20min. In session 3, mice explore the arena for 5min, after replacement of one of the objects with a different novel object (different color and shape) and are returned to the home cage. General activity near each object is tracked on the videos. Testing is repeated three times with different novel objects for comparison to baseline recognition. To quantify novel object preference, we use the exploratory preference index (EPI):

$$EPI = \left(\frac{Time\ at\ Novel\ Object}{Total\ time\ at\ Objects} * 100\right)$$

Object 1 or 2 times are substituted for Novel Object time in the numerator to determine if there is a preference between similar objects.

Win-shift T-maze foraging: companion to novel object recognition [4]. Spontaneous alteration behavior of mice is used to identify deficits in working memory and hippocampal dysfunction. T-shaped arena 30cm long x 10cm wide x 20cm high, with a 7cm central partition between the top arms, and guillotine doors that can be lowered to isolate these arms (Supplementary Fig. S6B). Mice are placed at the base of the T facing away from the top arms. The mice turn and traverse the stem, enter one of the top arms, and the associated door is lowered to allow 30s exploration of the arm. The mice are returned to their home cage for 2min, and the trial is repeated. Subsequent trials are repeated 10 times/day for 3 days. The maze is cleaned with 70% ethanol between trials.

Tail suspension: mice are suspended vertically by taping their tail to a standard retort stand clamp elevated 30cm above a table and video recorded [5]. Plastic tubing is placed over the tail to stop mice crawling up their tail during testing. Mice hang suspended for 6min. Mice are returned to their home cage. Total time spent moving versus time immobile (not moving other than that needed for adequate respiration) is subsequently measured from the video using a stopwatch and is interpreted as “giving up” which is a proxy for depression-like behavior.

Forced swim: two day (acclimation and test days) companion to tail suspension [6]. Each day, mice are placed in a 12cm diameter cylinder containing a 30cm column of water at 25°C for 10min on the first day and 6min on the second day. Mice are video recorded to determine time spent immobile versus swimming/struggling.

Barnes Maze: opaque white circular platform, 91cm diameter, with 30cm x 5cm holes around the circumference (7.6cm from the edge) [7]. An escape/goal box measuring 10cm x 10cm x 5cm is placed under one of the holes (Supplementary Fig. S6D). Mice associate the goal box with escape by placement inside the goal hole. Testing lasts 11 days, with 3 x 5min trials/day/mouse as follows: days 1–5, training to find and enter the goal hole. Day 6, a 90° clockwise maze rotation probe day. Mice should return to the training day location. Day 7, retraining day with the maze in the original orientation. Day 8, curtain probe day to block visual cues that the mice use to find the goal box, which determines if intrinsic property cues on the maze allow the mice to locate the goal hole (these cues must be eliminated). Day 9, mice are retrained as for Days 1–5 and 7. Day 10, the goal hole is rotated 180° from the original location. Mice are trained to the new location to determine how easily they relearn. Day 11, probe day to determine if the mice remember the day 10 goal hole location. The latency to reach the goal hole and the number of errors (defined as attempting to enter an incorrect hole) are recorded. Trials are video recorded for analysis using Ethovision tracking software (Noldus).

Contextual/cued fear conditioning: cued and contextual fear conditioning, fear extinction and reinstatement/renewal testing [8]. Conditioning chambers include contextually unique environment, removable shock grid floor, house light, speaker, and cue light (Coulbourn Instruments, Whitehall, PA). Prior to experimentation foot shock is optimized for each mouse. The intensity (0.3–1mA) and duration (0.5–2s) are varied to find the minimum shock needed to elicit an avoidance response. Contextual based conditioning is based on shock presentation after mice have habituated to the chamber and cue based conditioning pairs auditory or visual stimuli with foot shock.

Contextual fear conditioning is a 2 day procedure where animals are placed in a contextually unique chamber (normal lighting conditions without food or water during testing). After 3min acclimation, mice receive 3–5 foot shocks (0.5–2s) with varying interstimulus intervals (30–120s). Mice are returned to their home cages. After 24h, mice are returned to the chamber and video recorded for 10min to capture freezing behavior (without additional foot shocks).

Cued fear conditioning is a 2 day procedure of acclimation and exposure to 5–15 trials of cue-shock presentation (on day 1). Cues are short duration lights or tones (85dB, 16kHz, 20s) followed by foot shock (0.3–1mA) in the final 0.5–2s of cue presentation. After 24h, mice are tested for 3–10min for freezing behavior (no foot shock) to either the context of the chamber or presentation of a cue for 3–10min in a contextually distinct chamber.

Fear extinction testing is the same procedure for cued and contextual conditioning, and involves daily extinction sessions (no foot shock) for 12 days that are identical to the day 2 session for each test.

Reinstatement/renewal testing involves day 1 of cued fear conditioning in a distinct context ‘A’ in the chamber. Day 2, mice are exposed to two conditions, 3h apart: 1) context ‘B’ (distinct from context ‘A’) where mice are presented with 15 trials of light/tone (no foot shocks); 2) context ‘A’ for 30min (no foot shocks). Two days after conditioning (day 4), mice are separated into 2 groups and exposed to either context ‘A’ or ‘B’ (no foot shocks) or 15 trials of light/tone (no foot

shocks). Freezing behavior is measured to determine cued extinction retention and fear renewal. Mice are otherwise housed in their home cage.

Histology

To stain intact myelin, we used luxol fast blue (LFB) staining with stock solutions of: LFB Stain-1g Solvent Blue 38 (S-3382, Sigma, St. Louis, MO), 995ml 95% ethanol, 5ml 10% acetic acid that was mixed and filtered, and 0.1%(w/v) lithium carbonate (554-13-2, Arcos Organics, Geel, Belgium), and 1% Eosin-Y-1g Eosin-Y (E511–25, Sigma, St. Louis, MO) in 100ml of 95% ethanol. Frozen cryostat slides were thawed in 1X PBS, dehydrated through ethanol series to xylene for 2 x 2min washes and washed in 95% ethanol 2 x 2min. LFB stain was applied for 6–8h at 70°C followed by a 95% ethanol wash for 1min before moving to distilled water. Slides were differentiated in $LiCO_3$ followed by rinsing in 80% ethanol and washing in distilled water. Gill's No.1 hematoxylin (GHS-132, Sigma, St. Louis, MO) counterstain was applied for 5min, followed by washing, then working Eosin-Y stain (0.25% Eosin-Y in 100% ethanol and 0.5% acetic acid) for 30s followed by a tap water rinse.

To stain axons throughout the CNS we used the following reagents for Bielschowsky modified silver stain: 10% silver nitrate [9], 10% silver nitrate with ammonium hydroxide (added drop wise with stirring until the silver oxidizes then turns clear), developer stock solution – 80ml distilled H_2O, 20ml formaldehyde, 0.5g citric acid, 2drops 14N nitric acid, working developer solution – 16drops developer stock, 16drops ammonium hydroxide and 100ml distilled water, 5% (w/v) sodium thiosulfate, and ammonium water made of 16 drops ammonium hydroxide in 100ml water. All glassware was acid washed before staining. For staining, the 10% silver and 10% silver + ammonium were heated, covered, to 40°C and the slides thawed in 1X PBS followed by dehydration through ethanol series to xylene and back to distilled water. Slides were then moved to 10% silver for 1min at 40°C, washed in distilled water and placed in 10% silver + ammonium for 10min at 40°C. Next, slides were moved to the working developer solution where fiber tracks turn black during differentiation. To stop differentiation, slides were washed 3 x 2min in ammonium water, then washed in 5% sodium thiosulfate for 5min to chelate residual heavy metals. Nuclei were counterstained with Hematoxylin.

Immunocytochemistry

Frozen slides were thawed in 1X PBS and washed 3 x 5min. Slides were then permeabilized at either 25°C in methanol for 20min or 0.1% Triton X-100 (BP151-100, Fisher, Hampton, NH) for 1h. Slides were washed in 1X PBS, and the sections encircled with Pap Pen to confine antibody reagents in subsequent steps. Slides were blocked for either 30min in 1X Tris Buffered Saline with 0.5% glycine, 1% bovine serum albumin (Sigma) and 2% goat serum (Gts, 16210-064, Gibco, Waltham, MA) (TBSGBA), or in 0.1M Phosphate Buffer with 0.3% Triton X-100 and 10% goat serum (PBSTG) for 1h. Primary antibodies used were (Supplementary Table 1): mouse anti-NeuN, 1:1,000 (MAB377, EMD Millipore, Ellerica, MA), mouse anti-ankyrin-G, 1:300 (75-146, Antibodies Inc., Davis, CA), mouse anti-CC-1, 1:100 (OP80, EMD Millipore), mouse anti-GFAP, 1:1,000 (MO22136, Neuromics, Minneapolis, MN), rabbit anti-Iba-1, 1:1,000 (019-19741, Wako USA, Richmond, VA), rabbit anti-CHOP, 1:300 [10], and mouse anti-Kv7.2, 1:10 (73-079, Antibodies, Inc.) and were diluted in TBSGBA or PBSTG and incubated overnight at 25°C with gentle rocking (see Supplementary Table S1 for further details). Slides were washed in 1X PBS, followed by fluorescent secondary antibody incubation for 3h (1:1,000) and DAPI, 1:1,000 (D9542, Sigma) in TBSGBA or PBSTG with rocking. Slides were washed in 1X PBS, the Pap Pen was removed with chloroform on a Q-tip and Vectashield (H-1000, Vector Labs, Burlingame, CA) used to coverslip.

A pilot experiment in the brain from a single double heterozygote male mouse, *McreG::tdTomato* (B6;129S6-*Gt(ROSA)26Sor*$^{tm9(CAG\text{-}tdTomato)Hze}$/J), was used to determine the proportion of mature perivascular oligodendrocytes that could absorb sufficient tamoxifen from 2 successive daily gavages to activate cre-mediated recombination of the *tdTomato* transgene. Vibratome sections (100μm) were permeabilized with 1% Triton X-100 and labeled with mouse anti-CC-1 antibodies in TBSGBA (1:100 ,EMD Millipore), and through-focus images were generated at 40X magnification from confocal image stacks for analysis in imageJ. The number of $EGFP^+/CC1^+$ oligodendrocytes expressed as a proportion of total $CC1^+$ cells was expressed as a function of the distance from blood vessels (center-to-center measurements).

Entorhinal cortex AIS image analysis

Ten micrometer image stacks of 25–30 images (0.3μm spacing) were captured using a Leica DM5500 microscope (Leica Microsystems Inc, Buffalo Grove, IL) equipped with a Plan Apo 40x/0.85 coverslip correction lens, Yokagawa spinning disk confocal with Melles-Girot argon ion laser (McBain Systems, Westlake Village, CA), Orca R2 digital camera (Hamamatsu Corp, Bridgewater, NJ) and Metamorph software (version 7.6, Lake Zurich, IL). Maximum intensity projection for extended focus RGB images of ankyrin-G / NeuN / DAPI triple-positive cortex were analyzed in Fiji/ImageJ [11]. Each ankyrin-G^+ AIS in the analysis conformed to two criteria: the proximal end was juxtaposed to a NeuN/DAPI double-positive cell body; the distal end had a tapering railroad track appearance and did not terminate with a truncated square end. The freehand line tool was used to measure AIS length, which accommodates in-plane nonlinearity.

At least five AIS profiles were measured/image (213μm x 163μm fields), with six images of left and right cortices from three matched 10μm cryostat sections/mouse. The AIS lengths were plotted for each mouse as a cumulative frequency distribution (0–40μm, 2μm bins) and curves were averaged by genotype and fit with a Gaussian curve for statistical analysis.

Supplementary Information
Corticohippocampal Dysfunction In The *OBiden* Model Of Primary Oligodendrogliopathy
Daniel Z. Radecki, Elizabeth L. Johnson, Ashley K. Brown, Nicholas T. Meshkin, Shane A. Perrine and Alexander Gow

Hippocampus AIS analysis

Image stacks of the CA1 layer in dorsal (DHC) and ventral (VHC) hippocampus were captured as for cortex, except they comprised the central 8μm of image stacks from 10μm cryostat sections. The CA1 layer is defined as the cell dense layer directly dorsal (DHC) or lateral (VHC) to mid-dentate gyrus and representative of overall CA1 appearance to avoid confusion with the CA2 region. Individual AIS profiles are difficult to identify and measure in the granular CA1 layer; thus, ≥10 analysis rectangles 700 (110μm) x 50 (one cell body width) pixels centered over CA1 were measured and averaged to generate fluorescence intensity plots for each color. These RGB plots were generated separately for left and right hippocampal slices from three cryostat sections and averaged/mouse for integration under the curves and the final intensities plotted as functions of distance normal to the CA1 layer. Cognate plots were averaged from three mice/genotype.

Western Blotting

Mice are decapitated and the brain rapidly removed, cut into 2mm coronal slices using a brain matrix and frozen on dry ice. For tissue punches, a 1.5mm disposable biopsy punch (Integra Miltex, Integra, York, PA) at room temperature is attached to tubing with filter paper and air pressure is used to expel tissue from the punch. Brain slices were placed on an inverted glass petri dish covered with filter paper wetted with 0.9% saline (sodium chloride, S640, Fisher). Brain regions were quickly punched and expelled into -80°C Eppendorf tubes kept closed on dry ice.

Before removing samples from the freezer, phosphorylation preservation buffer was made, consisting of: 50mM HEPES (BP310, Fisher), 140mM potassium acetate (BP364, Fisher), 4mM sodium acetate (BP333, Fisher), 20mM sodium pyrophosphate (S6422, Sigma), 20mM β–glycerol phosphate (G6376, Sigma), 1mM dithiothreitol (DTT), 0.1mM PMSF, 0.1% protease inhibitor cocktail (P8340, Sigma), and 0.1% of phosphatase inhibitor cocktails 2 and 3 (P5726 and P0044, Sigma) in distilled water (Phos-pres). Phos-pres (50μl) was added to each protein punch on ice, and the samples were sonicated with 3 sonication pulse trains 5min apart, and each train consisted of 5 x 1s ON/2s OFF pulse pairs. Samples were kept on ice and only removed for the 15s sonication step. Protein concentration was determined using the Micro Bicinchoninic Acid (BCA, ThermoFisher Scientific) assay kit per the manufacturer instructions.

Samples were diluted in Phos-pres to 2X final concentration, then mixed 1:1 in general sample buffer [1g SDS (BP166, Fisher), 0.5g sucrose (BP220, Fisher), 0.98g Tris-HCL (BP153, Fisher), 25ml distilled H_2O, pH6.8 with bromophenol blue to equal concentrations for each sample. Samples were vortexed 3 x 5s each, then heated at 60°C for 5min and then spun down for 30s. Eight, 10 or 12%, 1.5mm thick, SDS-PAGE gels were cast with 10 or 15 well combs and samples electrophoresed at 25mA/gel with water circulated cooling. PVDF membranes were equilibrated in transfer buffer containing 20% methanol for at least 1h prior to transfer. Gels were stacked onto membranes and transferred, with cooling, at 500mA for 1h.

After transfer, membranes were blocked in 5% milk, 0.1% Tween-20 (BP337, Fisher) in 1X PBS (1X PBST) for 1h followed by addition of primary antibodies. Primary antibodies were diluted in 3% milk in 1X PBST with the following primary antibodies used: mouse anti-NeuN 1:5,000, mouse anti-APP 1:1,000, rabbit anti-NF-L, 1:2,000 (2837, Cell Signaling, Danvers, MA), mouse anti-non-phosphorylated-NF, 1:2,000 (SMI32P, BioLegend, San Diego, CA), mouse anti-ankyrin-G, 1:1,000, mouse anti-β4-spectrin, 1:500 (73-377, Antibodies Inc.), mouse anti-Nav1.2, 1:1,000 (75-042, Antibodies Inc.), mouse anti-Kv7.2, 1:100, rat anti-Ctip2, 1:1,000 (ab18465, Abcam), mouse anti-MBP, 1:5,000 (808401, Covance), rat anti-PLP1, 1:1,000 (AA3 hybridoma), mouse anti-CNP, 1:5,000 (SMI91R, Covance), and rabbit anti-α-tubulin, 1:5,000 (sc12462, Santa Cruz Biotech, Santa Cruz, CA). All primary antibodies were incubated on orbital shakers overnight at 4°C or 25°C. Membranes were washed in 1X PBST for 3 x 10min, before addition of secondary HRP-conjugated antibodies diluted 1:5,000 in 3% milk solution in 1X PBST. Secondary antibodies incubated for 1h followed by 1X PBST washes before detection through enhanced chemiluminescence (ECL) per the manufacturer instructions.

Auditory brainstem testing

Auditory brainstem recordings were measured in 2mo and 6mo mice under avertin (2,2,2-tribromoethanol) anesthesia using a 2 channel Intelligent Hearing Systems Universal box according to previously published methods [12]. The EEG signals were preamplified (x100,000), band pass filtered (0.3–3kHz) and recorded for 12.5ms, with 40kHz sampling, using 32kHz pure tone pips (102μs) between 80–50dB sound pressure level (SPL) as previously described (Sepwin software version 5.1, Intelligent Hearing Systems, Miami, FL) [13].

Magnetic resonance and diffusion tensor imaging

For T2 structural scans, all MR images were acquired on a Bruker 7T Clinscan horizontal bore magnet with Siemens Syngo MR B15 software and a dual coil mouse brain surface coil. Mice were anesthetized using isoflurane vapor at 1.5–3.0% in medical air to maintain 70–90breaths/min, and the MRI holder was maintained at 32–34°C using a heated water circulation system. The T2 scan parameters were as follows: TE=61ms, TR=1,200ms, TA~42min (depending on gating), 16mm x 16mm FOV, 180° flip angle, Turbo factor=22, 2 averages and 3D acquisition for in-plane resolution of 125μm x 125μm. Images were exported to OsiriX software (version 8, Pixmeo SARL, Bernex, Switzerland) for analysis and reconstruction.

The DTI images were acquired as follows: TE=30ms, TR=2,500ms, TA~30min (depending on gating), across 6 diffusion directions, 5 x 500μm thick slices with 1.2mm spacing and 2D acquisition. Two sets of scans were obtained, offset by 500μm to give greater coverage of the brain. Scans were imported into OsiriX and from the unprocessed 4D output, Eigen vector maps were calculated for each vector 1(λ1), 2(λ2) and 3(λ3) and the FA was calculated as:

$$FA = \sqrt{1/2} * \frac{\sqrt{(\lambda 1-\lambda 2)^2+(\lambda 2-\lambda 3)^2+(\lambda 3-\lambda 1)^2}}{\sqrt{\lambda 1^2+\lambda 2^2+\lambda 3^2}}$$

For each region analyzed, 8–10 pixels were used to calculate an average FA across the pixels and then regions were averaged across slices to get an overall FA for each region and each animal.

Statistical Analysis

All statistical analysis were performed using Prism (version 7; Graphpad Software Inc., La Jolla, CA). For longitudinal behavioral testing, 2-way ANOVAs were used to compare genotypes across ages to identify changes within ages and within groups across ages if necessary. Western blot analysis used 2-way ANOVA again to identify changes between genotypes, but also to identify any changes between proteins within groups, especially with AIS proteins as changes in opposing directions could signify degenerative changes. Kaplan-Meier plots with X^2 analyses were used to compare median lifespan of mutant mouse strains. Data are reported as mean ± SEM unless otherwise noted.

NEURON simulations

Computational modeling was performed using the NEURON simulation environment, version 7.4 [14]. The model is constructed with six identical dendrites connected individually to the soma. Each dendrite is comprised of proximal medial and distal segments. The soma is connected via a hillock segment to an axon with six AIS segments (three proximal and three distal) followed by an unmyelinated axon segment, the myelinated axon with 60 internodes and finally a small terminal segment. The internodes are taken directly from a previous model (ModelDB accession 122442) used to determine the function of myelin tight junctions [15,16]. Passive properties and active mechanisms are adapted from published sources, and the current study, and are listed in Supplementary Tables S2–4. Potassium channel densities in distal dendrites were adjusted to ensure approximately 50% reduction in amplitude 500μm from the soma [17]. Membrane resistance at the soma, R_m=21MΩ, was determined from the maximum slope of an I–V plot.

Simulations were performed at 37°C with extracellular Na^+=160mM, K^+=5mM and intracellular Na^+=30mM, K^+=140mM. We used d_lambda=0.01 for all compartments and ran simulations with fixed time steps=0.001ms and absolute tolerance=0.0001. All simulations began with 1ms initialization, followed by 7ms IClamp current injections applied at the midpoint of the soma. Threshold currents were determined iteratively to define the lowest current (±1pA) to generate a bAP and/or AP. The bAP amplitudes and voltage-time functions were measured in dendrite 6,389μm from the soma, and their magnitudes were not affected by lengthening the distal dendrite by 100μm (i.e. no segment boundary artifacts). The AIS voltage-time functions were measured at the midpoint of segments 1 (proximal) and 6 (distal) and the APs were measured at the midpoint of node 17. We also applied linear voltage ramps using VClamp (10–200V/s), with lengths defined by the bAP/AP threshold potentials. This method generated stimulus artifacts at ramp termination, but yielded qualitatively similar results to IClamp.

Neuropathology summaries of patients in the current study by Dr Roscoe Atkinson, MD

MS-4659: Neuropathological diagnosis:

1. Multiple sclerosis
2. Chronic/active multiple sclerosis plaque formation

Date Completed: 12-15-07

Gross Description: High quality, high resolution colored digital images of both hemispheres and 7mm full coronal sections at all levels are examined in a frontal to occipital direction. Four x 1cm sections of the brainstem and cerebellum are also examined. The coronal sections show irregular demyelinating periventricular plaque formation throughout the extent of the body of both lateral ventricles. There are also smaller focal areas of demyelination in the left temporal white matter, left thalamus, basilar pons, and pontine tegmentum. There is no other apparent softening, discoloration, hemorrhage, mass, or other lesion. There is no atrophy of the cerebellar folia. Significant atherosclerosis is not present
Microscopic description: A section of periventricular white matter is examined with the H&E stain and the luxol fast blue (LFB) myelin stain (8). A section of hippocampus and temporal lobe is also examined with the H&E stain (10). The sections show plaque formation with up to 80% axonal loss and 100% demyelination (LFB). There is near complete loss of oligodendrocytes. Associated gliosis is prominent in the bordering white matter. There are scattered macrophages with intracytoplasmic myelin seen along the edge of the plaque in the LFB-stained section. There is no evidence of perivascular lymphocytic cuffing. Comment: These findings are those of chronic/active multiple sclerosis (MS) plaque formation and are consistent with the diagnosis of multiple sclerosis.

Daniel Z. Radecki, Elizabeth L. Johnson, Ashley K. Brown, Nicholas T. Meshkin, Shane A. Perrine and Alexander Gow

MS-4663: Neuropathological diagnosis:

1. Multiple sclerosis
2. Chronic multiple sclerosis plaque formation

Date Completed: 12-14-07

Gross Description: High quality, high resolution colored digital images of both hemispheres and 7mm full coronal sections at all levels are examined in a frontal to occipital direction. Four x 1cm sections of the brainstem and cerebellum are also examined. The coronal sections show extensive irregular demyelinating periventricular plaque formation throughout the extent of the bodies of both lateral ventricles, the basal ganglia, brainstem, and cerebellar white matter. There is no other apparent softening, discoloration, hemorrhage, mass, or other lesion. The striatum, lentiform nuclei, and thalami are normal. There is no atrophy of the cerebellar folia. Significant atherosclerosis is not present. The spinal cord is also submitted and is grossly normal.

Microscopic description: A section of periventricular white matter is examined with the H&E stain and the luxol fast blue (LFB) myelin stain (8). The hippocampus and temporal lobe are also examined with the H&E stain (13). The sections show demyelinating plaque formation with up to 80% axonal loss and 100% demyelination (LFB). There is a marked decrease in oligodendrocyte density. Associated gliosis is minimal. There is no evidence of macrophage activity or perivascular lymphocytic cuffing. Comment: These findings are those of chronic multiple sclerosis (MS) plaque formation and are consistent with the diagnosis of multiple sclerosis:

MS-4725: Neuropathological diagnosis:

1. Multiple sclerosis
2. Chronic/active multiple sclerosis plaque formation

Date Completed: 5-4-08

Gross Description: High quality, high resolution colored digital images of both hemispheres and 7mm full coronal sections at all levels are examined in a frontal to occipital direction. Four x 1cm sections of the brainstem and cerebellum are also examined. The coronal sections show diffuse patchy irregular demyelinating periventricular plaque formation throughout the extent of both lateral ventricles. There is also involvement of both lentiform nuclei. There is also a vertically oriented centralized area of demyelination in the basilar pons. There is no other apparent softening, discoloration, hemorrhage, mass, or lesion. The striatum, lentiform nuclei, and thalami are normal. There is no atrophy of the cerebellar folia. Significant atherosclerosis is not present. The dura and spinal cord are also present and are grossly normal.

Microscopic description: A section of periventricular white matter is examined with the H&E stain and the luxol fast blue (LFB) myelin stain (10) The hippocampus (10) and temporal lobe (10) are examined with the H&E stain. The sections show plaque formation with up to 90% axonal loss and I 00% demyelination (LFB). There is a prominent decrease in oligodendrocyte density and extensive associated gliosis. There are scattered examples of associated perivascular lymphocytic cuffing. Macrophage activity is present but sparse. The neocortex contains normal neuronal cellularity and normal subcortical white matter. The hippocampus is also normal without evidence of neurodegeneration or hypoxia.

MS-4951: Neuropathological diagnosis:

1. Multiple sclerosis
2. Active multiple sclerosis plaque formation

Date Completed: 1-7-12

Gross Description: High quality, high resolution colored digital images of the entire brain and 7mm full coronal sections at all levels are examined in a frontal to occipital direction. Four x 1cm sections of the brainstem and cerebellum are also examined. There is no cerebral atrophy, cerebral edema, or herniation of the unci or cerebellar tonsils. The coronal sections show variable amount of irregular demyelinating periventricular plaque formations throughout the anterior body of the left lateral ventricle. There is similar plaque formation (0.6 x 0.4cm) involving right parietal lobe. There is no other apparent softening, discoloration, hemorrhage, mass, or lesion. The striatum, lentiform nuclei, and thalami are normal. There is no atrophy of the cerebellar folia. The basilar cerebral vasculature shows no significant atherosclerosis.

Microscopic description: Sections of left periventricular white matter (5) are examined with the H&E stain and the luxol fast blue (LFB) myelin stain. Sections of hippocampus (7A) and temporal lobe (78) are examined with the H&E stain. Sections of periventricular white matter shows plaque formation with up to 80% axonal loss and 70% demyelination (LFB). There is near complete oligodendrocyte loss, prominent associated gliosis and macrophage activity. Many of the macrophages contain LFB positive material. The adjacent white matter shows mild rarefaction.

MS-4961: Neuropathological diagnosis:

1. Multiple sclerosis
2. Chronic/active multiple sclerosis plaque formation

Date Completed: 1-2-12

Gross Description: High quality, high resolution colored digital images of the entire brain and 7mm full coronal sections at all levels are examined in a frontal to occipital direction. Four x 1cm sections of the brainstem and cerebellum are also examined. The coronal sections show extensive irregular demyelinating periventricular plaque formation throughout most of the bodies of both lateral ventricles with more anterior extension in the right hemisphere. There is similar subcortical plaque formation (0.6 x 0.4cm) present in the left parietal lobe. There is no other apparent softening, discoloration, hemorrhage, mass, or lesion. The striatum, lentiform nuclei, and thalami are normal. There is no atrophy of the cerebellar folia. Significant atherosclerosis is not present.

Microscopic description: Sections of right periventricular white matter (8) are examined with the H&E stain and the luxol fast blue (LFB) myelin stain. Sections of hippocampus (6A) and temporal lobe (68) are examined with the H&E stain. Sections of periventricular white matter show plaque formation with 80% axonal loss, 80% oligodendrocyte loss, 80% demyelination (LFB) and prominent gliosis. There is evidence of macrophage activity and moderate perivascular lymphocytic cuffing. The hippocampus is normal without evidence of neurodegeneration or hypoxia. Comment: These findings are those of chronic/active multiple sclerosis (MS) plaque formation and chronic multiple sclerosis plaque formation.

HC-3529: Neuropathological diagnosis:

1. Essentially normal brain.
2. No evidence of metastatic carcinoma.

Date Completed: 6-12-03

Gross Description: High quality, high resolution colored digital images of the entire brain and 7mm full coronal sections at all levels are examined m a frontal to occipital direction. Four x 1cm sections of the brainstem and cerebellum are also examined. The frontal, parietal, and temporal lobes show no atrophy. There is mild cerebral edema but no associated herniation of the hippocampal unci or cerebellar tonsils. There is no evidence of mass, lesion, hemorrhage, infarction, or metastatic disease. The substantia nigra shows normal pigmentation. The basilar cerebral vasculature shows no atherosclerosis.

Microscopic description: The hippocampus with adjacent temporal lobe (11) is examined with the H&E stain. There is normal neuronal cellularity with no evidence of neuropathology, hypoxia, or metastatic disease. Comment: These findings are unremarkable and show no evidence of pathology.

HC-3540: Neuropathological diagnosis:

1. Essentially normal brain.

Date Completed: 6-12-03

Gross Description: High quality, high resolution colored digital images of the entire brain and 7mm full coronal sections at all levels are examined in a frontal to occipital direction. Four x 1cm sections of the brainstem and cerebellum are also examined. There is minimal atrophy involving the frontal and temporal lobes. There is no evidence of mass, lesion, hemorrhage, infarction, or metastatic disease. The substantia nigra shows normal pigmentation. The basilar cerebral vasculature shows minimal atherosclerosis.

Microscopic description: The hippocampus with adjacent temporal lobe (11C), frontal lobe (2B), and pons (CBL1) are examined with the H&E stain. There is normal neuronal cellularity with no evidence of parenchymal neuropathology, hypoxia, or metastatic d1sease. The section of frontal lobe shows a small amount of subarachnoid hemosiderin. The section of pons shows a small m1ld focus of perivascular lymphocytes but is otherwise unremarkable.

Comment: These findings are unremarkable and show no evidence of significant pathology. There is also no evidence of metastatic disease. The small amount of subarachnoid hemosiderin likely reflects a remote (old) resolved mild focal subarachnoid hemorrhage.

HC-3504: Neuropathological diagnosis:

1. Essentially normal brain.
2. Incidental age related changes.
3. No evidence of cerebral metastatic disease.

Date Completed: 2-20-03

Gross Description: High quality, high resolution colored digital images of the entire brain and 7mm full coronal sections at all levels are examined m a frontal to occipital direction. Four x 1cm sections of the brainstem and cerebellum are also examined. There is no apparent softening, discoloration, hemorrhage, mass, or other lesion. The junction between the cortex and white matter is well demarcated. There is no grossly identifiable evidence of metastatic disease. The striatum, lentiform nucleus, hippocampus, and thalamus are normal. There is no atrophy of the cerebellar folia. Significant atherosclerosis of the basilar cerebral vasculature is not present.

Microscopic Description: A section of hippocampus (10) and adjacent temporal cortex (10) are examined with the H&E stain. There is normal neocortical and hippocampal neuronal cellularity. There is no extracellular spongiosis or gliosis. The hippocampus shows a few examples of granulovacuolar degeneration and scattered Hirano bodies. Significant formation of neurofibrillary tangles is not present. There is also no evidence of hemorrhage, necrosis, infection, or metastatic disease.
Comment: These findings show incidental age-related changes.

HC-3543: Neuropathological diagnosis:

1. Essentially normal brain.

Date Completed: 6-12-03

Gross Description: High quality, high resolution colored digital images of the entire brain and 7mm full coronal sections at levels are examined in a frontal to occipital direction. Four x 1cm sections of the brainstem and cerebellum are also examined. The frontal, parietal, and temporal lobes show no atrophy. There is no evidence of mass, lesion, hemorrhage, infarction or metastatic disease. The substantia nigra shows normal pigmentation. The basilar cerebral vasculature shows no atherosclerosis.
Microscopic description: The hippocampus with adjacent temporal lobe (10) is examined with the H&E stain. There is normal neuronal cellularity with no evidence of neuropathology, hypoxia, or metastatic disease.
Comment: These findings are unremarkable and show no evidence of pathology.

HC-3545: Neuropathological diagnosis:

1. Essentially normal brain.
2. No evidence of metastatic carcinoma.

Date Completed: 6-12-03

Gross Description: High quality, high resolution colored digital images of the entire brain and 7mm full coronal sections at all levels are examined in a frontal to occipital direction. Four I cm sections of the brainstem and cerebellum are also examined. The frontal, parietal, and temporal lobes show no atrophy. There is mild cerebral edema but no associated herniation. There is no evidence of mass, lesion, hemorrhage, infarction, or metastatic disease. The substantia nigra shows normal pigmentation. The basilar cerebral vasculature shows no atherosclerosis.
Microscopic description: The hippocampus with adjacent temporal lobe (12) is examined with the H&E stain. There is normal neuronal cellularity with no evidence of neuropathology, hypoxia, or metastatic disease.
Comment: These findings are unremarkable and show no evidence of pathology.

References

1 Readhead, C., Schneider, A., Griffiths, I. & Nave, K. A. Premature arrest of myelin formation in transgenic mice with increased proteolipid protein gene dosage. *Neuron* **12**, 583-595 (1994).
2 Deacon, R. M. Measuring the strength of mice. *J Vis Exp*, e2610 (2013).
3 Leger, M. *et al.* Object recognition test in mice. *Nat Protoc* **8**, 2531-2537 (2013).
4 Deacon, R. M. & Rawlins, J. N. T-maze alternation in the rodent. *Nat Protoc* **1**, 7-12 (2006).
5 Steru, L., Chermat, R., Thierry, B. & Simon, P. The tail suspension test: a new method for screening antidepressants in mice. *Psychopharmacology (Berl)* **85**, 367-370 (1985).
6 Porsolt, R. D., Bertin, A., Blavet, N., Deniel, M. & Jalfre, M. Immobility induced by forced swimming in rats: effects of agents which modify central catecholamine and serotonin activity. *Eur J Pharmacol* **57**, 201-210 (1979).
7 Barnes, C. A. Memory deficits associated with senescence: a neurophysiological and behavioral study in the rat. *J Comp Physiol Psychol* **93**, 74-104 (1979).
8 Chang, C. H. *et al.* Fear extinction in rodents. *Curr Protoc Neurosci* **Chapter 8**, Unit8 23 (2009).
9 Garven, H. S. & Gairns, F. W. The silver diammine ion staining of peripheral nerve elements and the interpretation of the results: with a modification of the Bielschowsky-Gros method for frozen sections. *Q J Exp Physiol Cogn Med Sci* **37**, 131-142 (1952).
10 Southwood, C. M., Garbern, J., Jiang, W. & Gow, A. The unfolded protein response modulates disease severity in Pelizaeus-Merzbacher disease. *Neuron* **36**, 585-596 (2002).
11 Schindelin, J. *et al.* Fiji: an open-source platform for biological-image analysis. *Nat Methods* **9**, 676-682 (2012).
12 Maheras, K. J., Pindolia, K., Wolf, B. & Gow, A. Developmental window of sensorineural deafness in biotinidase-deficient mice. *J Inherit Metab Dis*, doi: 10.1007/s10545-10017-10049-z (2017).
13 Maheras, K. J. & Gow, A. Increased anesthesia time using 2,2,2-tribromoethanol-chloral hydrate with low impact on mouse psychoacoustics. *Journal of neuroscience methods* **219**, 61-69 (2013).
14 Hines, M. L. & Carnevale, N. T. The NEURON simulation environment. *Neural Comput* **9**, 1179-1209 (1997).
15 Devaux, J. & Gow, A. Tight junctions potentiate the insulative properties of small CNS myelinated axons. *J Cell Biol* **183**, 909-921 (2008).
16 Gow, A. & Devaux, J. A model of tight junction function in central nervous system myelinated axons. *Neuron Glia Biol* **4**, 307-317 (2008).
17 Waters, J., Schaefer, A. & Sakmann, B. Backpropagating action potentials in neurones: measurement, mechanisms and potential functions. *Prog Biophys Mol Biol* **87**, 145-170 (2005).
18 Gencic, S. & Hudson, L. D. Conservative amino acid substitution in the myelin proteolipid protein of jimpymsd mice. *J Neurosci* **10**, 117-124 (1990).
19 Gow, A. in *Meth Enzymol* Vol. 491 (ed P. Michael Conn) 143-161; PMC3070952 (Academic Press, 2011).
20 Gow, A., Friedrich, V. L., Jr. & Lazzarini, R. A. Myelin basic protein gene contains separate enhancers for oligodendrocyte and Schwann cell expression. *J Cell Biol* **119**, 605-616 (1992).
21 Stecca, B. *et al.* The evolution of lipophilin genes from invertebrates to tetrapods: DM-20 cannot replace proteolipid protein in CNS myelin. *J Neurosci* **20**, 4002-4010 (2000).
22 Gow, A., Southwood, C. M. & Lazzarini, R. A. Disrupted proteolipid protein trafficking results in oligodendrocyte apoptosis in an animal model of Pelizaeus-Merzbacher disease. *J Cell Biol* **140**, 925-934 (1998).
23 Billings-Gagliardi, S., Adcock, L. H. & Wolf, M. K. Hypomyelinated mutant mice: description of jp^{msd} and comparison with *jp* and *qk* on their present genetic backgrounds. *Brain Res.* **194**, 325-338 (1980).
24 Lappe-Siefke, C. *et al.* Disruption of Cnp1 uncouples oligodendroglial functions in axonal support and myelination. *Nat Genet* **33**, 366-374 (2003).
25 Southwood, C. M. *et al.* Dimethyl fumarate ameliorates myoclonus stemming from protein misfolding in oligodendrocytes. *J Neurochem* **142**, 103-117 (2017).
26 Gow, A. *et al.* Deafness in Claudin 11-null mice reveals the critical contribution of basal cell tight junctions to stria vascularis function. *J Neurosci* **24**, 7051-7062 (2004).
27 Wenngren, B. I. & Anniko, M. A frequency-specific auditory brainstem response technique exemplified in the determination of age-related auditory thresholds. *Acta Otolaryngol* **106**, 238-243 (1988).
28 Shah, S. N. & Salamy, A. Auditory-evoked far-field potentials in myelin deficient mutant quaking mice. *Neuroscience* **5**, 2321-2323 (1980).
29 Gardinier, M. V. & Macklin, W. B. Myelin proteolipid protein gene expression in jimpy and jimpy(msd) mice. *J Neurochem* **51**, 360-369. (1988).
30 Hu, W. *et al.* Distinct contributions of Na(v)1.6 and Na(v)1.2 in action potential initiation and backpropagation. *Nat Neurosci* **12**, 996-1002 (2009).
31 Halter, J. A. & Clark, J. W., Jr. A distributed-parameter model of the myelinated nerve fiber. *J Theor Biol* **148**, 345-382 (1991).

32 Peters, A. The node of Ranvier in the central nervous system. *Q J Exp Physiol Cogn Med Sci* **51**, 229-236 (1966).
33 Berthold, C.-H. & Rydmark, M. in *The Axon* (eds S.G. Waxman, J.D. Kocsis, & P.K Stys) 13-48 (Oxford University Press, 1995).
34 Kirschner, D. A., Ganser, A. L. & Caspar, D. L. D. in *Myelin.* (ed P. Morell) 51-95 (Plenum, 1984).
35 Chiu, S. Y. & Schwarz, W. Sodium and potassium currents in acutely demyelinated internodes of rabbit sciatic nerves. *J Physiol* **391**, 631-649 (1987).
36 Battefeld, A., Tran, B. T., Gavrilis, J., Cooper, E. C. & Kole, M. H. Heteromeric Kv7.2/7.3 channels differentially regulate action potential initiation and conduction in neocortical myelinated axons. *J Neurosci* **34**, 3719-3732 (2014).
37 Stuart, G., Schiller, J. & Sakmann, B. Action potential initiation and propagation in rat neocortical pyramidal neurons. *J Physiol* **505 (Pt 3)**, 617-632 (1997).
38 Stuart, G. J. & Sakmann, B. Active propagation of somatic action potentials into neocortical pyramidal cell dendrites. *Nature* **367**, 69-72 (1994).

Supplementary Information

Corticohippocampal Dysfunction In The *OBiden* Model Of Primary Oligodendrogliopathy

Daniel Z. Radecki, Elizabeth L. Johnson, Ashley K. Brown, Nicholas T. Meshkin, Shane A. Perrine and Alexander Gow

Supplementary Fig. S1 – Alleles, tamoxifen dosing and initial characterization of *OBiden* (*OBi*) mice.

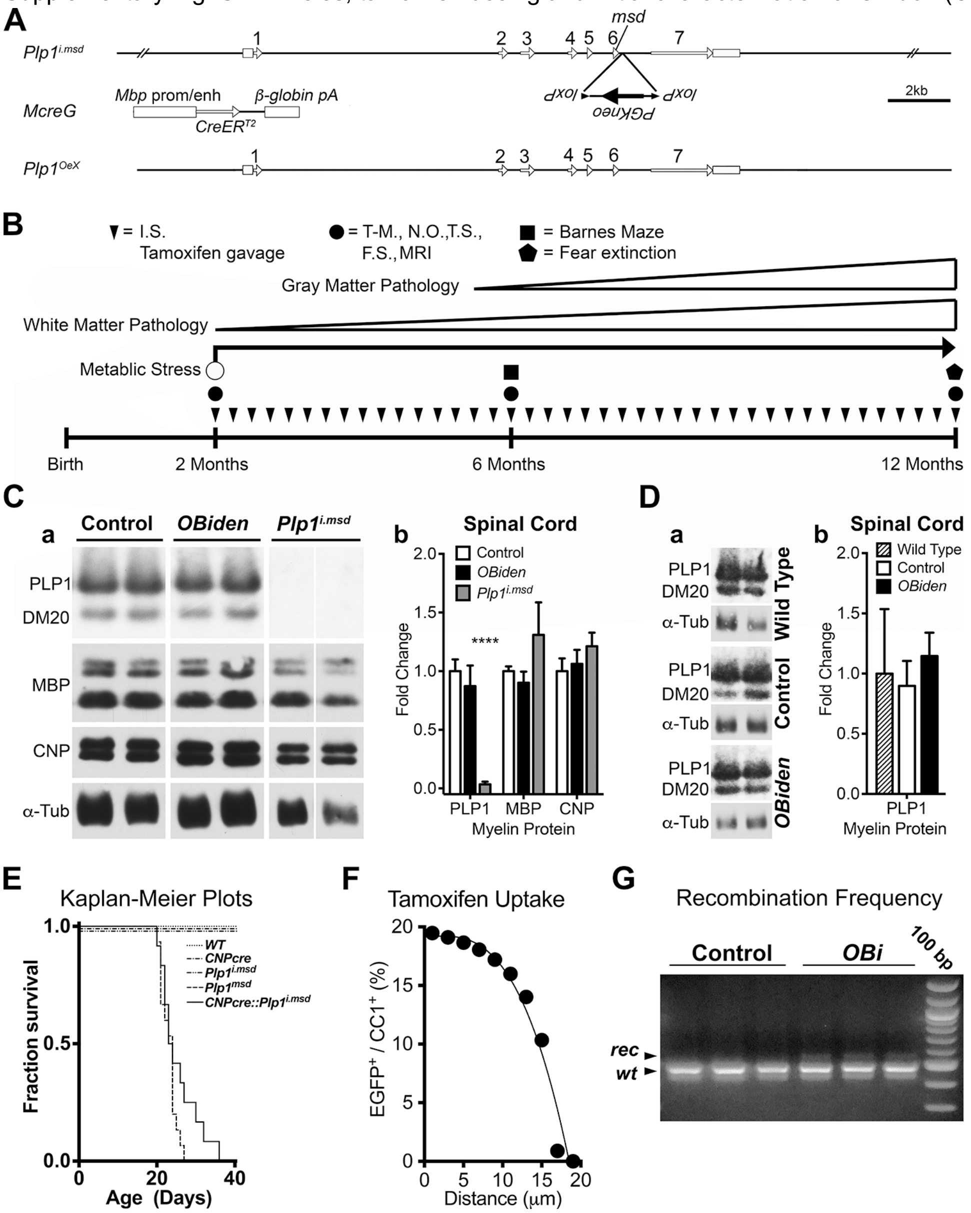

A) A combination of 3 alleles define the *OBi* mouse genotype (*Mus stimulus*). Allele 1: $Plp1^{i.msd}$, a *cre*-inducible mutant isoform of the endogenous *Plp1* gene, which harbors the *myelin-synthesis-deficient* point mutation encoding A243V [18]. Somatic recombination in oligodendrocytes by Cre induces metabolic stress and activates the UPR [10,19]. Allele 2: *McreG,* an autosomal transgene expressing $CreER^{T2}$ under control of the mouse *Mbp* promoter/enhancer in myelinating oligodendrocytes, [20]. Allele 3: $Plp1^{OeX}$, autosomal wild type *Plp1* transgene [1], to dosage-compensate for a lack of wild type *Plp1* expression. **B)** Weekly low-dose tamoxifen gavage causes somatic recombination of the floxed-*PGKneo* cassette in the $Plp1^{i.msd}$ allele [21] of some *OBi* oligodendrocytes, which activates mutant PLP1 expression and induces metabolic stress. Tamoxifen gavage and testing schedule: inverted screen (I.S.); Win-Shift T-maze foraging (T-M.); novel object (N.O.); tail suspension (T.S.); forced swim (F.S.); magnetic resonance imaging (MRI), Barnes maze, fear extinction. Estimated timelines for WM and GM pathology. **C)** Western blots of cervical and thoracic spinal cord myelin proteins from **(a)** 12mo controls, *OBi* and $Plp1^{i.msd}$ mice **(b)** quantification, shows similar PLP levels in control and *OBi* mice ($p>.7$). Also shown for MBP and CNP between control and $Plp1^{i.msd}$ mice ($p>.1$). As expected, $Plp1^{i.msd}$ mice do not express PLP gene products. **D)** Western blots of cervical/thoracic spinal cord in **(a)** 12mo wild type, control and *OBi* mice **(b)** quantification, show similar PLP1 expression between genotypes ($p>.8$). **E)** To determine if developmental expression of the $Plp1^{i.msd}$ allele in (A) could confer an early death phenotype equivalent to the naturally-occurring $Plp1^{msd}$ severely hypomyelinating mutant mouse characterized previously [10,22,23], we bred $Plp1^{i.msd}$ mice with constitutively-expressing *CNPcre* mice [24] (i.e. $Plp1^{i.msd}$*:: CNPcre*). The *Cnp* gene is expressed in oligodendrocyte progenitors from mid-gestation. Wild type (*WT*) and single mutant allelic *CNPcre* or $Plp1^{i.msd}$ mice do not exhibit overt phenotypes and live beyond 12mo of age (Kaplan-Meier curves are offset on the ordinate axis for visibility). Overall, the Kaplan-Meier curves are statistically different ($X^2_{(4)} = 73.5$; $p < .0001$), but $Plp1^{msd}$ and *CNPcre::*$Plp1^{i.msd}$ curves are indistinguishable ($X^2_{(1)} = 2.29$; $p = .13$), with median survival times of 24 ($n = 15$) and 23.4 ($n = 12$) days, respectively. *CNPcre::*$Plp1^{i.msd}$ mice phenocopy the $Plp1^{msd}$ mutants with respect to ataxia, spasticity, likely severe myoclonus [25] immediately prior to death and pervasive translucent pallor of white matter tracts in brain and spinal cord. **F)** We determined the efficiency of tamoxifen uptake by oligodendrocytes in vivo after breeding *CAG-tdTomato* mice (cat 007905; Jackson Laboratories, Bar Harbor, ME) with $Plp1^{i.msd}$*::McreG* mice (i.e. *CAG-tdTomato::*$Plp1^{i.msd}$*::McreG*). Cre recombination enables EGFP expression in myelinating oligodendrocytes, which we used for quantification as a proportion of total $CC1^+$ oligodendrocytes. Tamoxifen uptake in pons and brainstem 7days after 2 x daily oral gavages shows that approximately 20% of the perivascular oligodendrocytes absorb sufficient drug for recombination of the *tdTomato* allele (n=1 mouse, 4 vibratome sections). This proportion decreases with distance from vessels and overall approximates 10% within 3 cell diameters and 5% overall. **G)** Agarose gel showing PCR amplification of genomic DNA purified from the optic nerves of 3 *OBi* and 3 control mice at 18mo. All mice have the strong PCR product from the wild type allele (*wt*), but only *OBi* mice have the larger product from the Cre-recombined allele (*rec*) which encodes $PLP1^{i.msd}$. Image J analysis of the relative intensities of the *wt* and *rec* alleles, after local background subtraction, indicates a recombination frequency of 25.4±2.5%. This is likely a steady state level reflecting recombination in cells from successive rounds of tamoxifen gavage. We cannot detect the recombinant allele in tissue from a single tamoxifen gavage using this approach. Abbreviations: PLP1/DM20, proteolipid protein 1; MBP, myelin basic protein; CNP, 2',3'-cyclic-nucleotide 3'-phosphodiesterase; α-Tub, α-Tubulin (loading control). See unprocessed western blots below.

Supplementary Information

Corticohippocampal Dysfunction In The *OBiden* Model Of Primary Oligodendrogliopathy

Daniel Z. Radecki, Elizabeth L. Johnson, Ashley K. Brown, Nicholas T. Meshkin, Shane A. Perrine and Alexander Gow

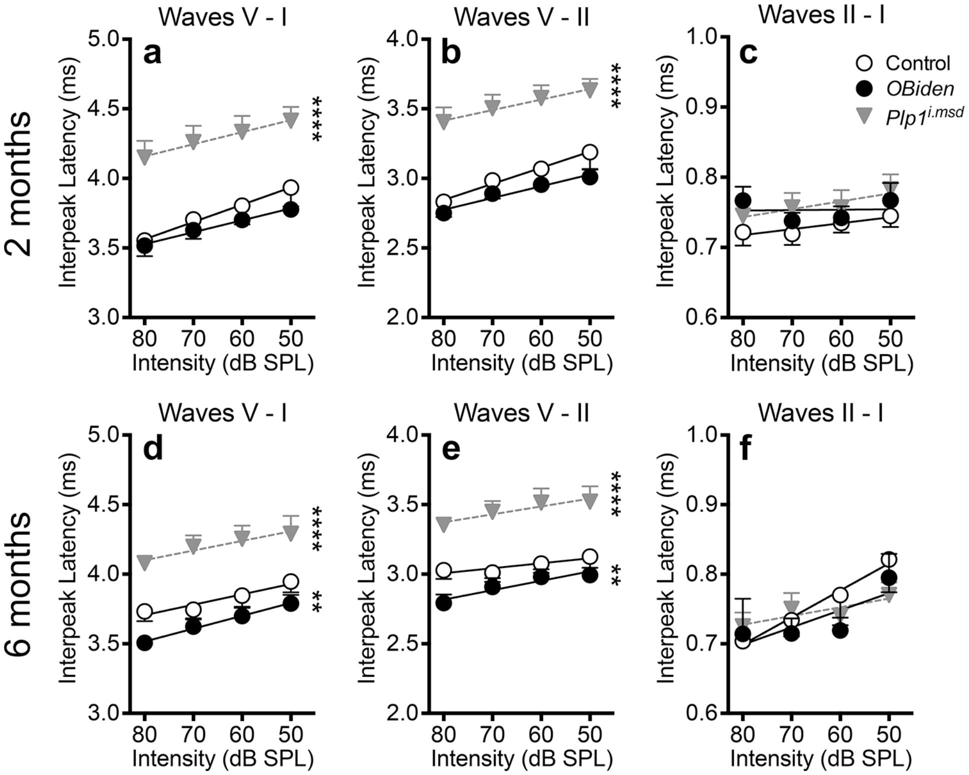

Supplementary Fig. S2 – Normal auditory brainstem transmission times in *OBi* mice. Auditory brainstem responses for control, *OBi* and *Plp1*$^{i.msd}$ mice at 2 and 6mo were acquired as previously described [26], using two channel recordings from reference (inverting) mastoid electrodes and a non-inverting vertex electrode under avertin anesthesia. Interpeak latencies between waves I and V (V – I, overall PNS + CNS components), waves II and V (V – II, CNS component) and waves I and II (II – I, PNS component) as functions of sound pressure level (SPL) for 32kHz pure tone pips. Interpeak latencies are inversely proportional to SPL, and used as a non-invasive proxy of conduction velocity in myelinated auditory brainstem fibers [27,28]. **(a,b)** Overall (a) and CNS (b) latencies in 2mo control and *OBi* mice are indistinguishable (linear regression unshared Y-intercept/slope, p>.14), but *Plp1*$^{i.msd}$ overall and CNS latencies are increased, indicating slowed conduction velocity in myelinated fibers (Y-intercept/slope, p<.0001). **(c)** PNS latencies are similar for all genotypes (Y-intercept/slope, p>.07). **(d,e)** Overall (d) and CNS (e) latencies at 6mo are increased in *Plp1*$^{i.msd}$ (Y-intercept/slope, p<.0001). *OBi* latencies are decreased (Y-intercept/slope, p<.007). **(f)** PNS latencies are similar between genotypes (Y-intercept/slope, p>.3).

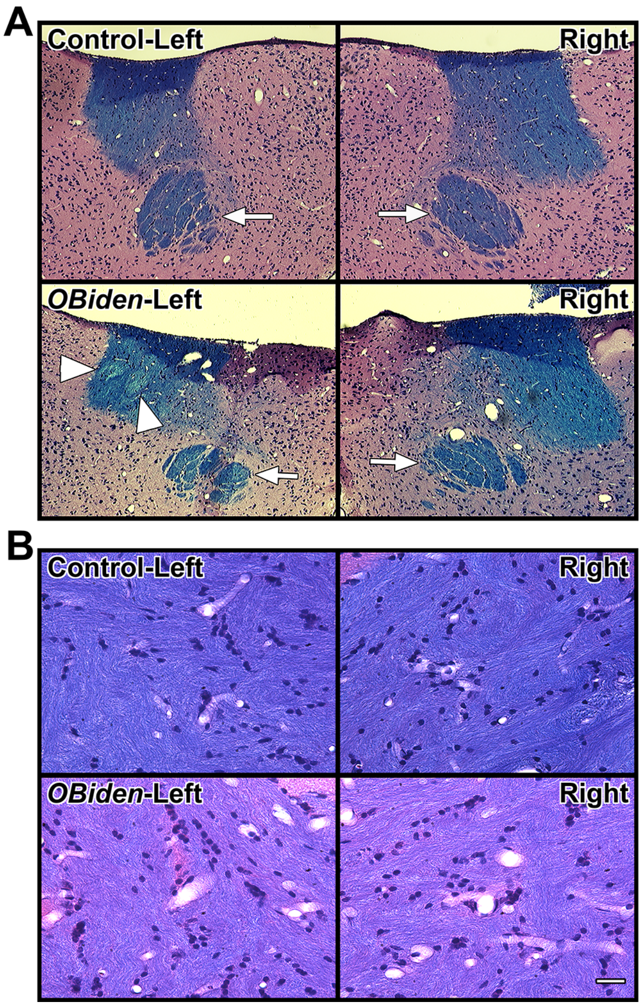

Supplementary Fig. S3 – Histopathology of *OBi* mice

Histopathology of WM lesions in *OBi* mice revealed by luxol fast blue–hematoxylin & eosin (LFB–H&E) staining. **A)** The LFB staining of myelin for several fiber tracts in periventricular coronal sections shows uniformly dark blue LFB staining of stria medullaris in 12mo control mice (upper panels). The staining is generally uniform in littermate *OBi* mice; however, at least 2 focal demyelinated/remyelinating shadow-like plaques are visible in left stria medullaris (lower panels, arrowheads). In the event that these lesions are remyelinating, the repair is likely a function of physiologically-normal myelin synthesized by wild type oligodendrocytes because the myelinating capacity of oligodendrocytes expressing mutant proteins from the $Plp1^{i.msd}$ allele is only a few percent of normal [29]. The contralateral tract in the *OBi* mouse and other WM tracts (arrows) are evenly stained suggesting minimal pathology. **B)** Internal capsule from coronal sections is weakly stained with LFB in *OBi* mice, reflecting bilateral generalized hypomyelination. However, we do not observe perivascular cuffing in the mutants, suggesting that infiltrating immune cells do not play a major role in the pathology out to at least 12 months.

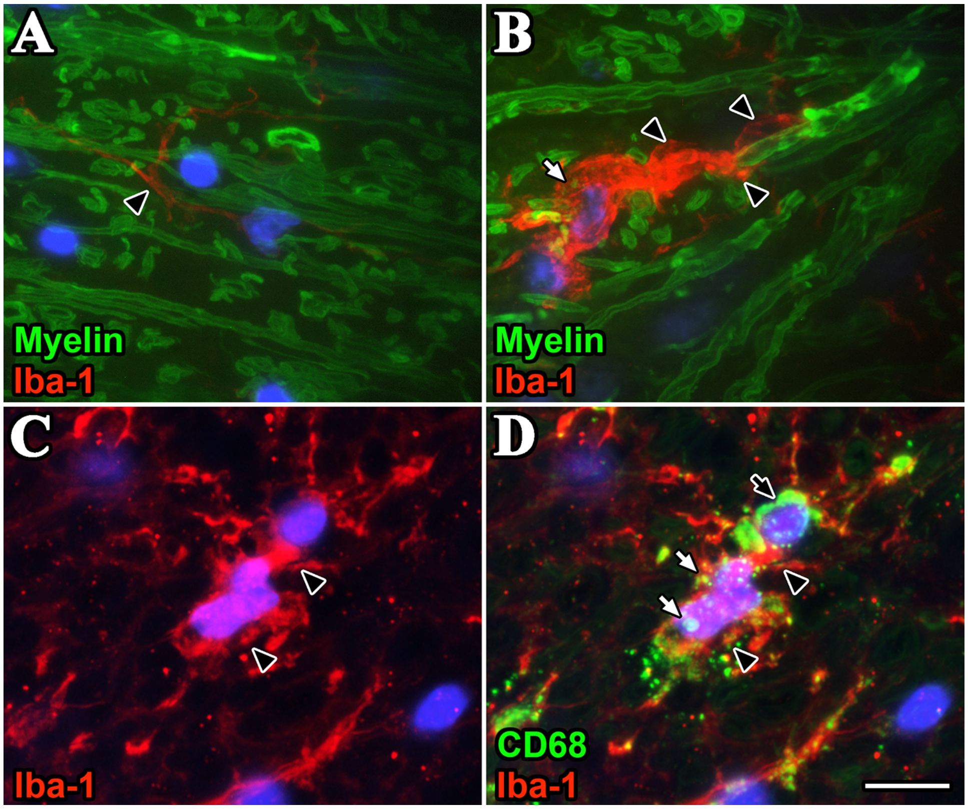

Supplementary Fig. S4 – Macrophages in the brainstem corticospinal tract ingesting myelin debris in *OBi* mice
A) Confocal image of well myelinated (EGFP from *dtTomato*) region (longitudinal section) from a 6mo control mouse showing a resting microglial cell (black arrowhead) labeled with antibodies against Iba-1 (red). Nuclei are labeled with DAPI (blue). **B)** Corticospinal tract of a 6mo *OBi* mouse showing a large activated microglial cell contacting a myelinated axon. The microglial cell body (left) has extended a leading process around the sheath (black arrowheads), the right hand end of which is associated with apparent myelin debris (bright green blob). In addition, the left hand end of the cell body appears to be associated with myelin debris. Further, the cell body may contain ingested myelin fragments in the cytoplasm (white arrow). **C)** A large activated Iba-1+ microglial cell (black arrowheads) in close proximity to or contacting another cell. **D)** Same field as in (C) showing that the microglial cell cytoplasm contains many CD68+ lysosomes (white arrows) and that this cell may be in the process of engulfing or attacking the adjacent cell (black arrow). Scale bar, 10μm.

Supplementary Information
Corticohippocampal Dysfunction In The *OBiden* Model Of Primary Oligodendrogliopathy
Daniel Z. Radecki, Elizabeth L. Johnson, Ashley K. Brown, Nicholas T. Meshkin, Shane A. Perrine and Alexander Gow

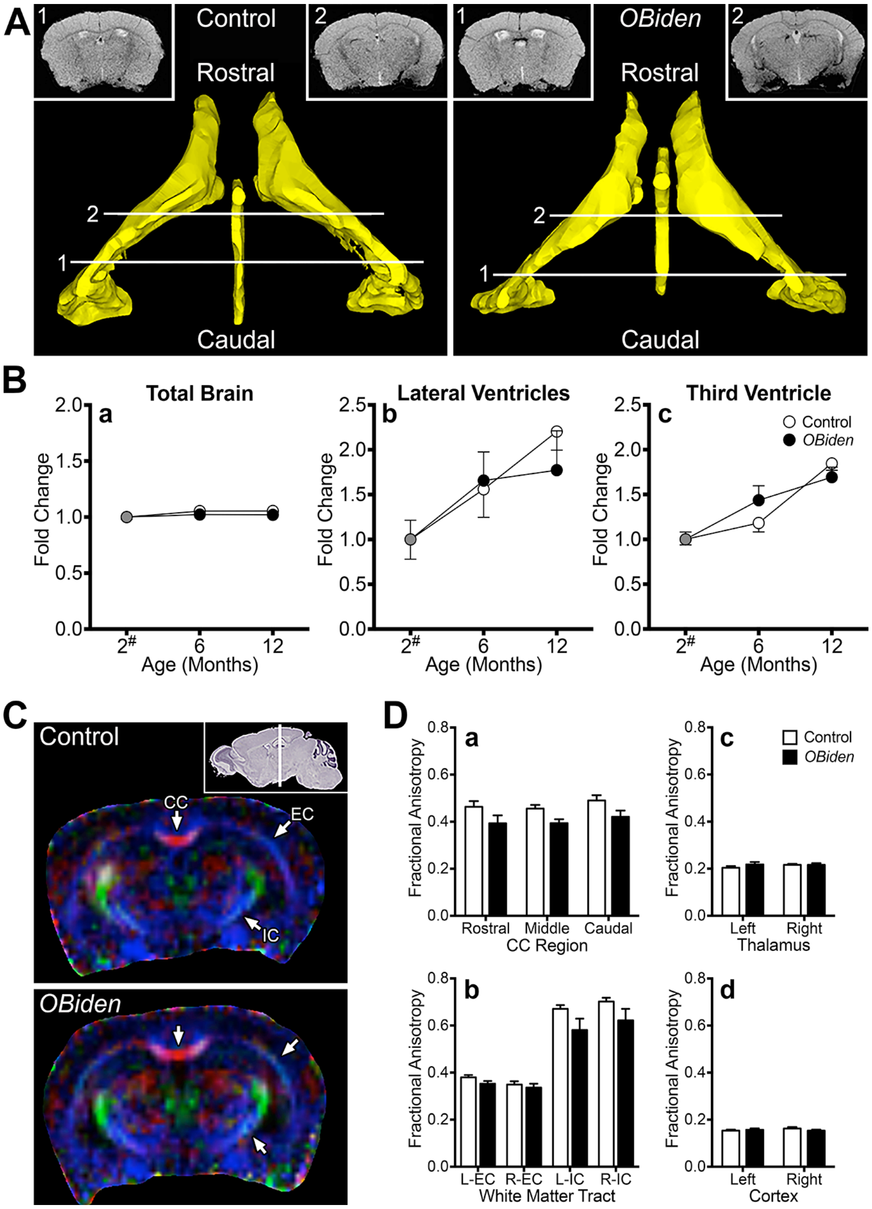

Supplementary Fig. S5 – MR imaging of *OBi* mice
Magnetic resonance imaging (MRI) and diffusion tensor imaging (DTI) analyses. **A)** Reconstructed lateral and third ventricles of 12mo control and *OBi* mice (yellow) from high resolution T2* slices (insets 1,2). **B)** Volumetric analyses from baseline MRI scans at 2mo (2#) to 12 mo, show no evidence of periventricular atrophy in *OBi* mice when normalized to average volume at 2mo. **(a)** Total brain volumes are indistinguishable by genotype and age (p>.6), **(b)** lateral ventricles increase with age but are indistinguishable by genotype (p>.7) and **(c)** third ventricle increases with age but is indistinguishable by genotype (p>.2). **C)** Representative coronal DTI images of major WM structures used for analyses in the current study: CC, EC and IC. Inset, DTI scan location in the sagittal plane. **D)** Fractional anisotropy measurements in *OBi* and control mice at 12mo from **(a)** 3 regions of CC, **(b)** left / right EC and IC, **(c)** thalamus immediately adjacent to EC and IC in (b) and, **(d)** entorhinal / perirhinal cortex immediately adjacent to EC in (b). There are no differences observed by genotype in CC (p>.14). Although there are overall regional differences between EC and IC in *OBi* mice and controls (p<.0001), there are no differences between genotypes (p>.09). Fractional anisotropies in GM immediately lateral and medial to EC are much lower than for WM tracts and are not different between genotypes (p>.4).

Supplementary Information

Corticohippocampal Dysfunction In The *OBiden* Model Of Primary Oligodendrogliopathy

Daniel Z. Radecki, Elizabeth L. Johnson, Ashley K. Brown, Nicholas T. Meshkin, Shane A. Perrine and Alexander Gow

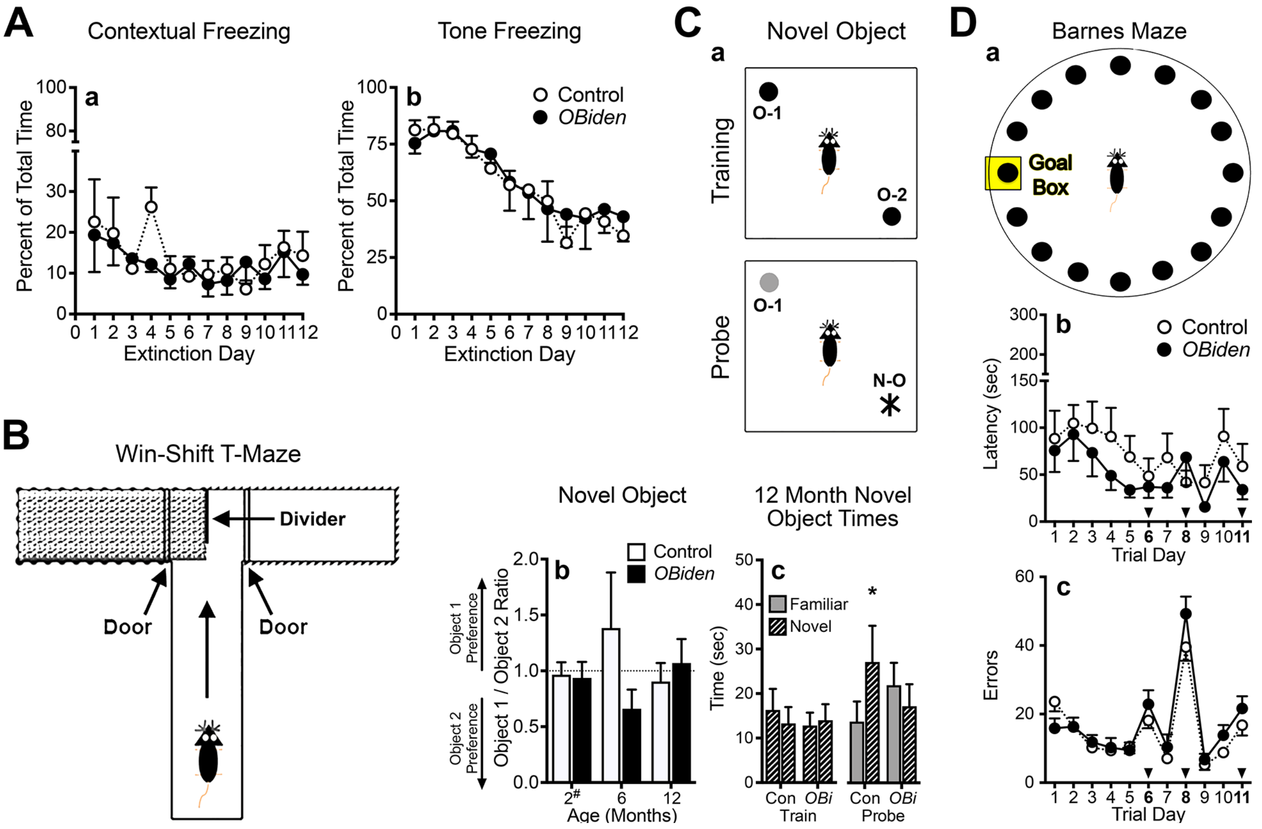

Supplementary Fig. S6 – Supplemental details of behavioral testing for *OBi* mice

Supplementary data, schematics and behavioral test results from *OBi* mice and littermate controls. **A)** In the fear extinction test, we observe no differences between controls and *OBi* mice for **(a)** contextual freezing during training trials (*p*>.4) or **(b)** tone-induced freezing behavior during fear extinction (*p*>.9). **B)** Diagram of Win-Shift T-Maze and mouse placement. The divider separates two goal arms, each of which has a guillotine door at the entrance. The walls are uniquely patterned to provide visual cues. For tactile cues, the left goal arm has a rough textured floor and the right arm has a smooth floor. **C) (a)** Diagram of the novel object arena on Training day (upper) with the locations of the mouse and objects, O-1 and O-2 (both of which are novel objects during training). The Probe day arena (lower) shows locations of the mouse, familiar object O-1 (gray) and the novel object (N-O). **(b)** Object preference (ratio of dwell times for O-1 and O-2) on the Training day shows no differences from 2mo baseline (2#)–12mo between control and *OBi* mice (multiple *t*-tests, *p*>.3). **(c)** Dwell times around objects O-1 and O-2 on Training day for control and *OBi* mice at 12mo shows no preference for either object (*p*>.5). On Probe day, control mice spend significantly less time around the familiar object, O-1, than around the novel object, N-O (*p*<.03), but *OBi* mice show no preference for N-O (*p*>.8). **D) (a)** Diagram of Barnes maze indicating starting position of the mice and goal box location for training days. **(b)** Average latencies to enter the goal box are similar between control and *OBi* mice overall (*p*>.8) and on Probe days (arrows) 6, 8 and 11 (*p*>.7). **(c)** Errors made while locating the correct goal box hole are similar between genotypes overall (*p*>.1) and on Probe days (*p*>.2).

Supplementary Information
Corticohippocampal Dysfunction In The *OBiden* Model Of Primary Oligodendrogliopathy
Daniel Z. Radecki, Elizabeth L. Johnson, Ashley K. Brown, Nicholas T. Meshkin, Shane A. Perrine and Alexander Gow

A

Patient #	Gender	Status	MS Type	Age(yr)	PMI(hr)	COD	Pair#	Histology	Westerns
HC-3529	M	Normal	-	58	9	CC	1		X
HC-3540	M	Normal	-	68	11	LC	2	X	
HC-3504	M	Normal	-	80	11	RC	3	X	X
HC-3543	F	Normal	-	73	12	COPD	4	X	X
HC-3545	M	Normal	-	80	14	BC	5		X
**Avg±SD**				72±9.2	11.3±1.9				
MS-4961	F	Chr/Active	PPMS	54	9	MS	1	X	X
MS-4663	M	Chronic	SPMS	62	16	MS	2	X	
MS-4725	F	Chr/Active	PPMS	44	29	MS, D	3	X	X
MS-4659	F	Chr/Active	SPMS	63	21	MS	4		X
MS-4951	F	Chr/Active	PPMS	50	25	MS	5		X
**Avg±SD**				55±8	20±7.6				

B

Cause of Death
MS - Multiple Sclerosis
PPMS - Primary Progressive MS
SPMS - Secondary Progressive MS
D - Dementia
CC - Colon Cancer
LC - Lung Cancer
RC - Renal Carcinoma
COPD - Chronic Obstructive Pulmonary Disease
BC - Bladder Cancer

C

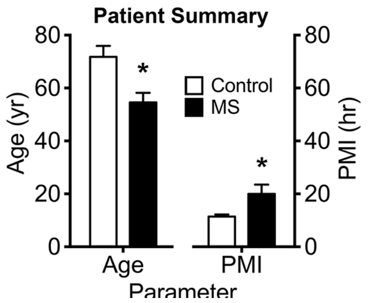

D

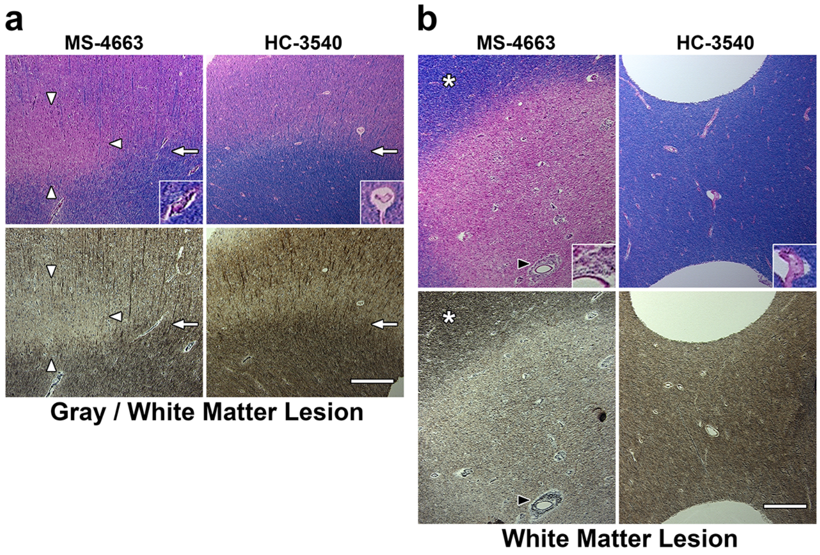

Supplementary Fig. S7 – Details of autopsy samples from MS patients and histopathology
Summary of autopsy brain samples provided by the University of California, Los Angeles, Human Brain and Spinal Fluid Research Center **A)** Deidentified brain autopsy samples (expedited IRB # 042414M1X, protocol # 1404012948) from frontal cortex of MS and non-neurological patients. Samples were used for histological analyses and western blotting. **B)** Summary Cause of death legend for patients in this study. **C)** The average age of MS patients is significantly lower than controls (multiple *t*-tests, 55±8 vs. 72±9 years, $p<.03$); thus, degenerative changes observed in MS samples most likely reflect disease progression rather than age-associated changes. There is also a modest increase in postmortem interval between MS patients and controls (multiple *t*-tests, 20±8 vs. 11±2h, $p<.05$). **D)** Luxol Fast Blue (upper) and Bielschowsky (lower) staining of cryostat sections from frontal cortex of MS patient #4663 (MS-4663) and a matched healthy control (HC-3540) shows demyelinating lesions (bounded by white arrowheads) **(a)** at the deep gray/white matter border (arrow) without immune infiltration (insets), corresponding to a loss of myelin and **(b)** within normal appearing white matter (asterisk) surrounding a blood vessel with immune infiltration apparent from perivascular cuffing (black arrowhead). Scale bars, 500µm or 150µm (insets).

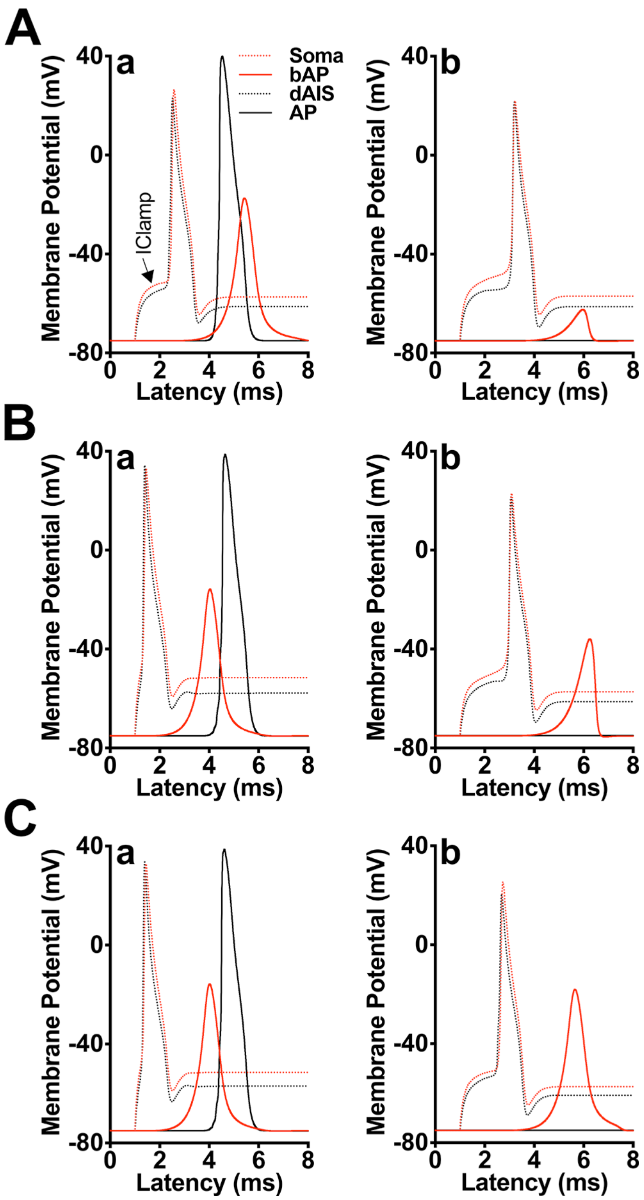

Supplementary Fig. S8 – Computational modeling data for cortical neurons in *OBi* mice

Current clamp simulations of rostral entorhinal cortical neurons under several conditions to determine the amplitudes of bAPs. **A)** wild type condition (AIS=20.6μm, Fig. 5C) **(a)** before demyelination with current clamp at the AP threshold **(b)** after demyelination with current clamp at the bAP threshold. Depolarization threshold >20 V/s, [30] of the distal AIS always precedes that of the soma and occurs at a lower membrane potential, as expected from the asymmetric distributions of Nav1.6 and Nav1.2 channels in the AIS and soma. The wild type condition triggers a bAP and an AP of full amplitude. Under demyelinating conditions at the bAP threshold current, AIS and soma depolarizations are relatively normal but the bAP is of low amplitude and is not associated with an AP. Indeed, this simulation reflects current block, whereby a 10-fold increase in depolarizing current fails to induce an AP. Modulating AIS length does not restore conduction (not shown). **B,C)** Remyelination with a single myelin lamella rescues the conduction block for both **(B)** wild type and **(C)** *OBi* (AIS=17.8μm, Fig. 5D) AIS lengths. Full amplitude bAPs and APs are elicited for wild type and *OBi* simulations at the AP threshold current **(Ba,Ca)**. However, these currents are approximately 2-fold higher than the corresponding bAPs in each simulation **(Bb,Cb)**. Moreover, the bAP amplitude elicited from the wild type AIS is small, while a shorter AIS in the *OBi* simulation generates a full amplitude bAP. These simulations reveal a potential mechanism by which neurons may compensate for pathological changes in the vicinity of the cell body, such as proximal demyelination, and maintain robust bAP signaling. bAP, back propagating action potential; AP, action potential; dAIS, distal region of the AIS.

Supplementary Information
Corticohippocampal Dysfunction In The *OBiden* Model Of Primary Oligodendrogliopathy
Daniel Z. Radecki, Elizabeth L. Johnson, Ashley K. Brown, Nicholas T. Meshkin, Shane A. Perrine and Alexander Gow

Supplementary Table S1 – Antibodies used for immunocytochemistry and western blotting

Target	Symbol	Isotype	Dilution	Permeabilization*	Company	Product#
Oligodendrocytes						
APC	CC-1	Ms IgG_{2b}	1:100	Meth	Calbiochem	OP80
PLP1	AA3	Rt IgG	1:300	Meth	hybridoma	
MBP	SMI99	Ms IgG_{2b}	1:500	Meth	Covance/Biolegend	808401
MBP	SMI94	Ms IgG_1	1:1,000	Meth	Covance	SMI-94R
CNP	SMI91	Ms IgG_1	1:1,000	Meth	Covance	SMI91-R
Microglia						
CD68		Rt IgG_{2a}	1:500	Saponin	AbD	MCA1957
IBA-1		Rb IgG	1:1,000	Tx/Meth	Wako	019-19741
Astrocytes						
GFAP		Ms IgG_1	1:1,000	Tx	Neuromics	MO22136
Neurons						
APP	22C11	Ms IgG_1	1:100	Meth	Millipore	MAB348
NFL		Rb IgG	1:100	Meth	Cell Signaling	2837
p-NFH	SMI31	Ms IgG_1	1:1,000	Tx/Meth	Covance	SMI-31P
n-NFH	SMI32	Ms IgG_1	1:1,000	Meth	Covance	SMI-32P
Ankyrin-G		Ms IgG_{2a}	1:300	Tx	Antibodies Inc	75-146
β4-spectrin		Ms IgG_{2b}	1:10	Tx	Antibodies Inc	73-377
Nav1.2		Ms IgG_{2a}	1:100	Tx/Meth	Antibodies Inc	73-024
Kv7.2		Ms IgG_1	1:10	Tx	Antibodies Inc	73-079
Nuclei						
Ctip2		Rt IgG_{2a}	1:500	Tx	Abcam	ab18465
NeuN		Ms IgG_1	1:1,000	Any	Millipore	MAB377
DAPI		-	1:1,000	Any	Sigma	D9542

* Meth, methanol; Tx, Triton X-100

Supplementary Table S2 – Myelin compartment passive properties for simulations

Parameter		Value	Units
Axon diameter, all compartments [15]		1.44	μm
Myelin compartment length:	node [31]	1.25	μm
	4 paranode segments [15]	1.42	μm/seg
	juxtaparanode [15]	21.7–23	μm
	5 internode segments [15]	21.7–23	μm/seg
Periaxonal space width:	node [31]	2E-3	μm
	Paranode [32]	2E-3	μm
	Internode [33]	2E-2	μm
Myelin period [34]		1.6E-2	μm
g-ratio [15]		0.8	
Axoplasm resistivity [31]		70	Ohm.cm
Axon membrane capacitance [31]		1	$\mu F.cm^{-2}$
Leak conductance*:	node [31]	0.156	$S.cm^{-2}$
	paranode [35]	5E-4	$S.cm^{-2}$
	juxtaparanode [35]	5E-3	$S.cm^{-2}$
	internode [35]	5E-3	$S.cm^{-2}$
Myelin periaxonal resistivity, all compartments [31]		70	Ohm.cm
Tight junction resistivity [15]		600	$Ohm.cm^2$
Myelin capacitance [31]		0.5	$\mu F.cm^{-2}$
Myelin membrane resistivity [15]		300	$Ohm.cm^2$

* equilibrium potential= -75mV

Supplementary Table S3 – Non-myelin compartment passive properties for simulations

Parameter		Value	Units
Dendrites (diameter, length):	proximal	2, 30	μm
	medial	0.8, 30	μm
	distal	0.4, 540	μm
Soma (diameter, length)		10, 10	μm
Hillock (diameter, length)		2, 2	μm
AIS and unmyelinated axon, diameter		1.44	μm
AIS and unmyelinated axon, combined length		120.6	μm
AIS, length:	3 proximal segments	0.5–10	μm/seg
	3 distal segments	0.5–10	μm/seg
Axon terminus (diameter, length)		2, 1	μm
Cytoplasm resistivity [15]		70	Ohm.cm
Membrane capacitance [15]		1	$\mu F.cm^{-2}$
Leak conductance*, all compartments [15]		5E-3	$S.cm^{-2}$

* equilibrium potential = -75mV

Supplementary Table S4 – Non-myelin compartment active properties for simulations

Compartment		Nav1.6 (pS.μm^{-2})	Nav1.2 (pS.μm^{-2})	Nav (cm.s^{-1})	Kf (cm.s^{-1})	Ks (cm.s^{-1})
Dendrite [A,B,C]:	proximal	0	340	0	1.4E-4	8.9E-6
	medial	0	340	0	6E-4	1.07E-5
	distal	0	340	0	8E-4**	3E-5**
Soma [A,B]		0	340	0	2.79E-4	8.33E-6
Hillock [A]		0	1.2E4	0	1.4E-3	8.33E-6
AIS, proximal segment [A]:	1	1.19E3	1.36E4	0	3.49E-3	8.33E-6
	2	4.25E3	1.58E4	0	5.58E-3	8.33E-6
	3	7.65E3	1.28E4	0	7.67E-3	8.33E-6
AIS, distal segment [A]:	1	10.0E3	6.8E3	0	1.05E-2	1.28E-5
	2	8.5E3	1.7E3	0	1.26E-2	3.78E-5
	3	6.63E3	85	0	1.4E-3	7.5E-5
Unmyelinated axon [A]		0	0	1.6E-3	2.79E-5	0
Node [A,D]		3E4*	0	0	2.79E-4	8.33E-5
Paranode [D]		0	0	3.39E-5	0	0
Juxtaparanode [D]		0	0	3.39E-4	2.79E-3	0
Internode [D]		0	0	3.39E-4	2.79E-3	0
Axon terminus [D]		0	0	3.39E-4	2.79E-5	0

A [30]; *B* [36]; *C* [37,38]; *D* [15]
* adjusted to generate 115mV action potential
** adjusted in the wild type neuron to generate a 50% decrease in bAP amplitude in the dendritic arbor 500μm from the soma [17]

Supplementary Information
Corticohippocampal Dysfunction In The *OBiden* Model Of Primary Oligodendrogliopathy
Daniel Z. Radecki, Elizabeth L. Johnson, Ashley K. Brown, Nicholas T. Meshkin, Shane A. Perrine and Alexander Gow

Blots for Figure 4Ba
Control: lanes 5&6; *OBiden*: lanes 3&4

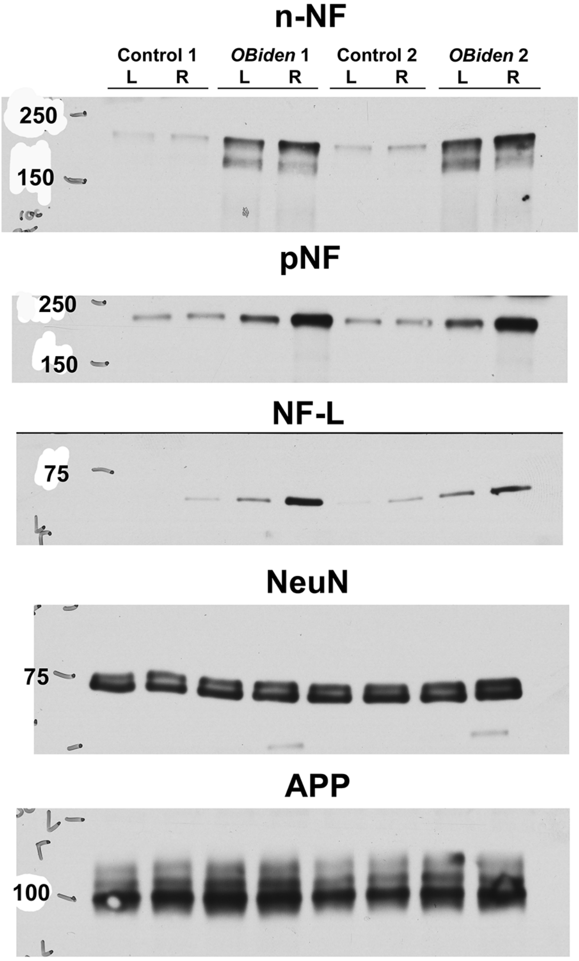

Supplementary Information
Corticohippocampal Dysfunction In The *OBiden* Model Of Primary Oligodendrogliopathy
Daniel Z. Radecki, Elizabeth L. Johnson, Ashley K. Brown, Nicholas T. Meshkin, Shane A. Perrine and Alexander Gow

Blots for Figure 4Ca
Control: lanes 1&2; *OBiden*: lanes 3&4

n-NF

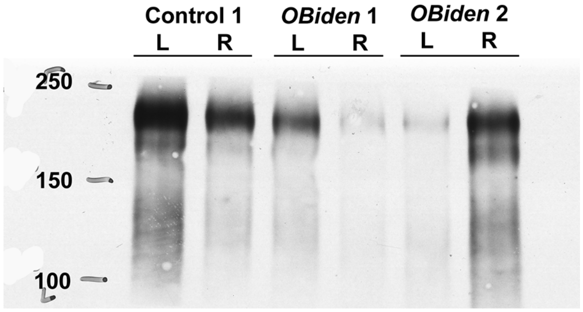

pNF

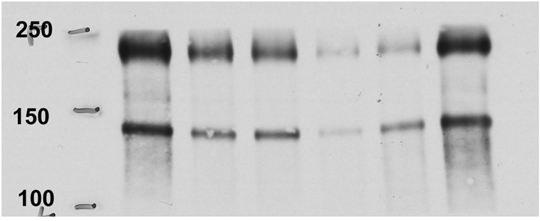

NF-L

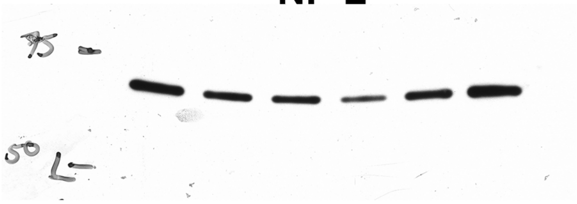

NeuN

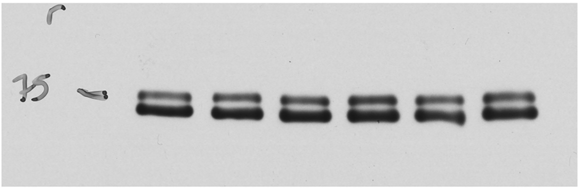

APP

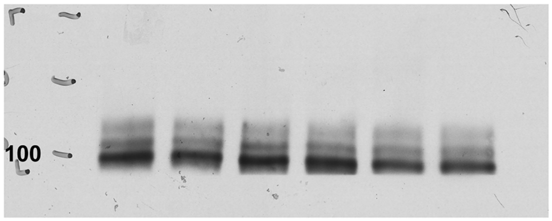

Supplementary Information
Corticohippocampal Dysfunction In The *OBiden* Model Of Primary Oligodendrogliopathy
Daniel Z. Radecki, Elizabeth L. Johnson, Ashley K. Brown, Nicholas T. Meshkin, Shane A. Perrine and Alexander Gow

Blots for Figure 4Da
Control: lanes 1&2; *OBiden*: lanes 3&4

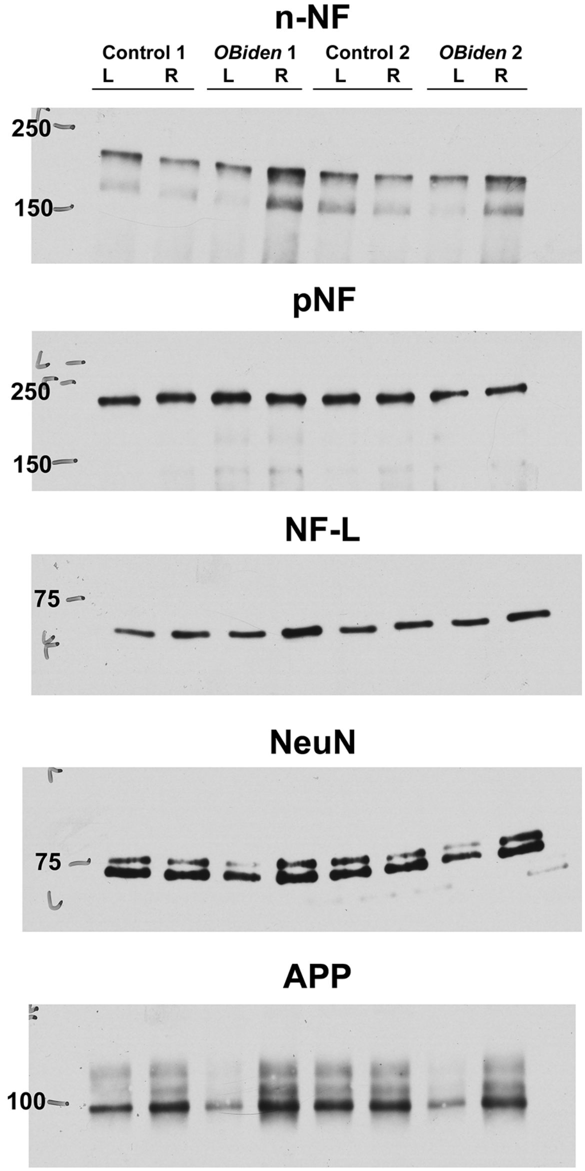

Supplementary Information
Corticohippocampal Dysfunction In The *OBiden* Model Of Primary Oligodendrogliopathy
Daniel Z. Radecki, Elizabeth L. Johnson, Ashley K. Brown, Nicholas T. Meshkin, Shane A. Perrine and Alexander Gow

Blots for Figure 5Cb
Control: lanes 1&2; *OBiden*: lanes 5&6

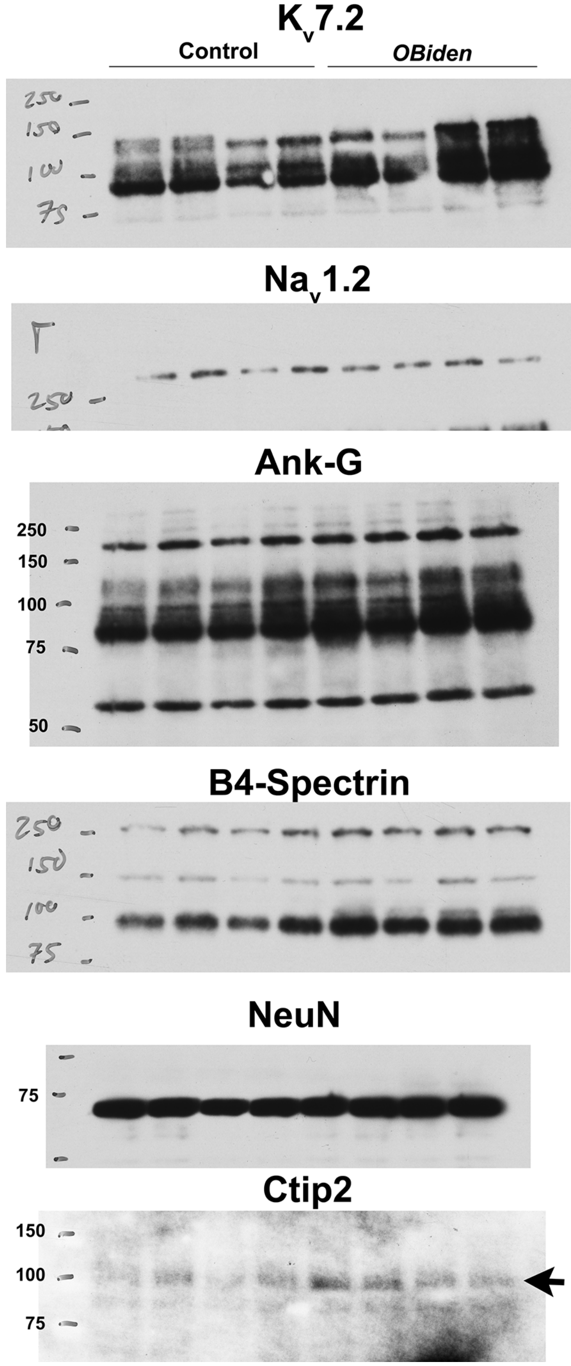

Supplementary Information
Corticohippocampal Dysfunction In The *OBiden* Model Of Primary Oligodendrogliopathy
Daniel Z. Radecki, Elizabeth L. Johnson, Ashley K. Brown, Nicholas T. Meshkin, Shane A. Perrine and Alexander Gow

Blots for Figure 5Db
Control: lanes 3&4; *OBiden*: lanes 5&6

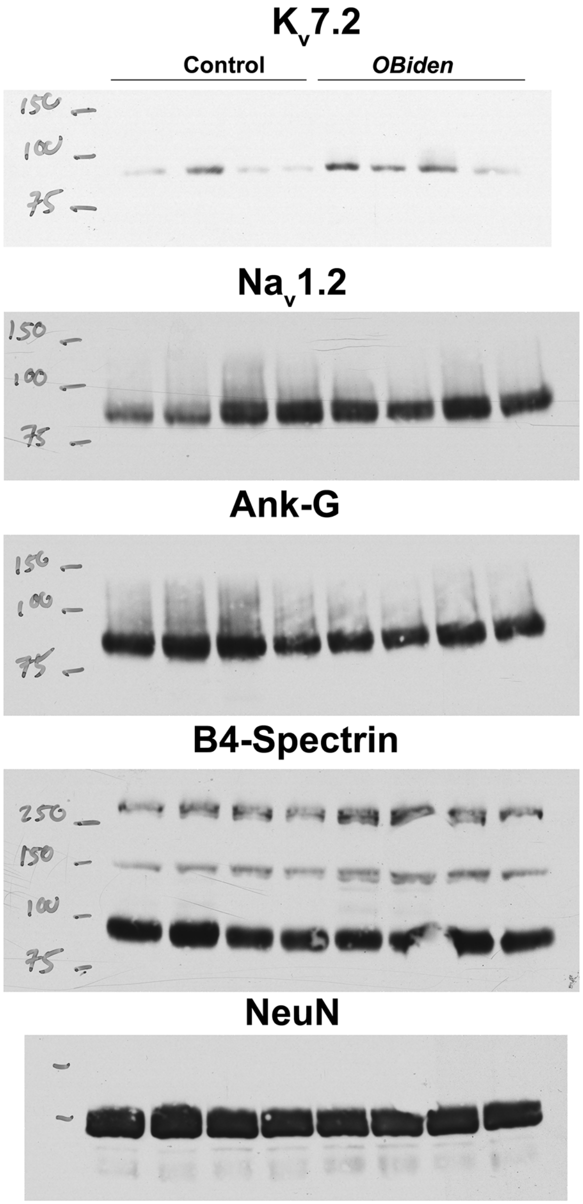

Supplementary Information
Corticohippocampal Dysfunction In The *OBiden* Model Of Primary Oligodendrogliopathy
Daniel Z. Radecki, Elizabeth L. Johnson, Ashley K. Brown, Nicholas T. Meshkin, Shane A. Perrine and Alexander Gow

Blots for Figure 6Ea
Control: lanes 1&2; *OBiden*: lanes 5&6

$K_v7.2$

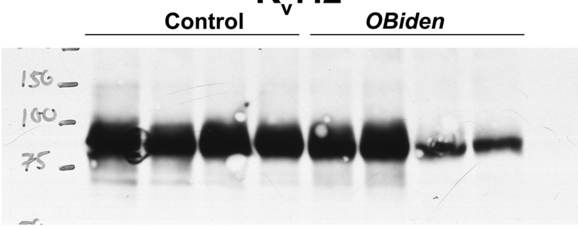

$Na_v1.2$

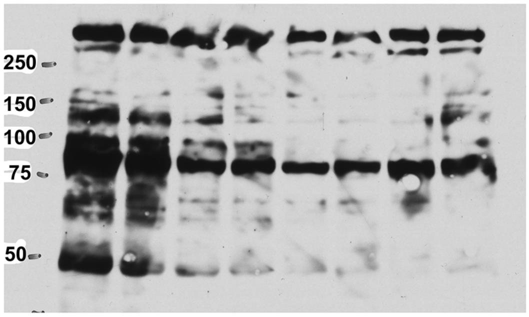

Ank-G

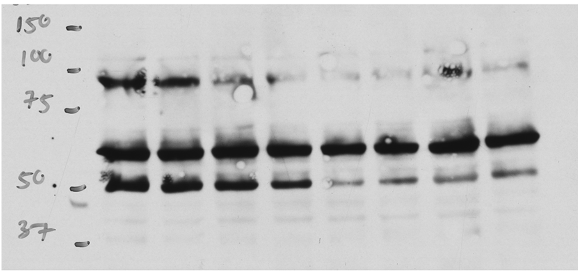

B4-Spectrin

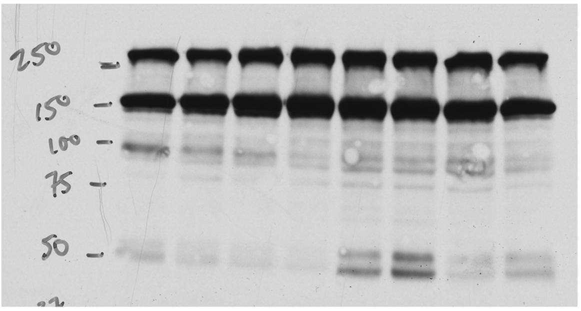

NeuN

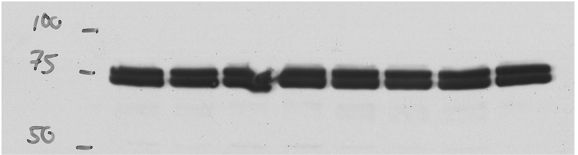

Supplementary Information

Corticohippocampal Dysfunction In The *OBiden* Model Of Primary Oligodendrogliopathy

Daniel Z. Radecki, Elizabeth L. Johnson, Ashley K. Brown, Nicholas T. Meshkin, Shane A. Perrine and Alexander Gow

Blots for Figure 6Fa

Control: lanes 1&2; *OBiden*: lanes 5&6

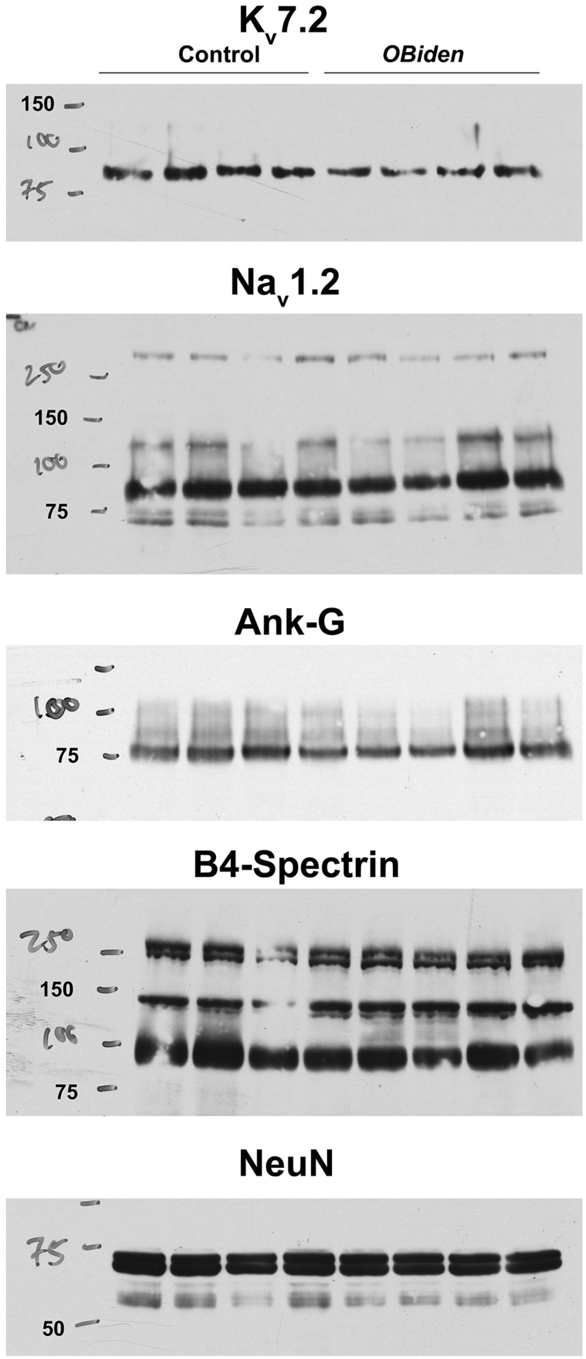

Supplementary Information

Corticohippocampal Dysfunction In The *OBiden* Model Of Primary Oligodendrogliopathy

Daniel Z. Radecki, Elizabeth L. Johnson, Ashley K. Brown, Nicholas T. Meshkin, Shane A. Perrine and Alexander Gow

Blots for Figure 7Aa

Left: Control: lane 2; MS: lane 4

Right: Control: lane 2; MS: lane 6

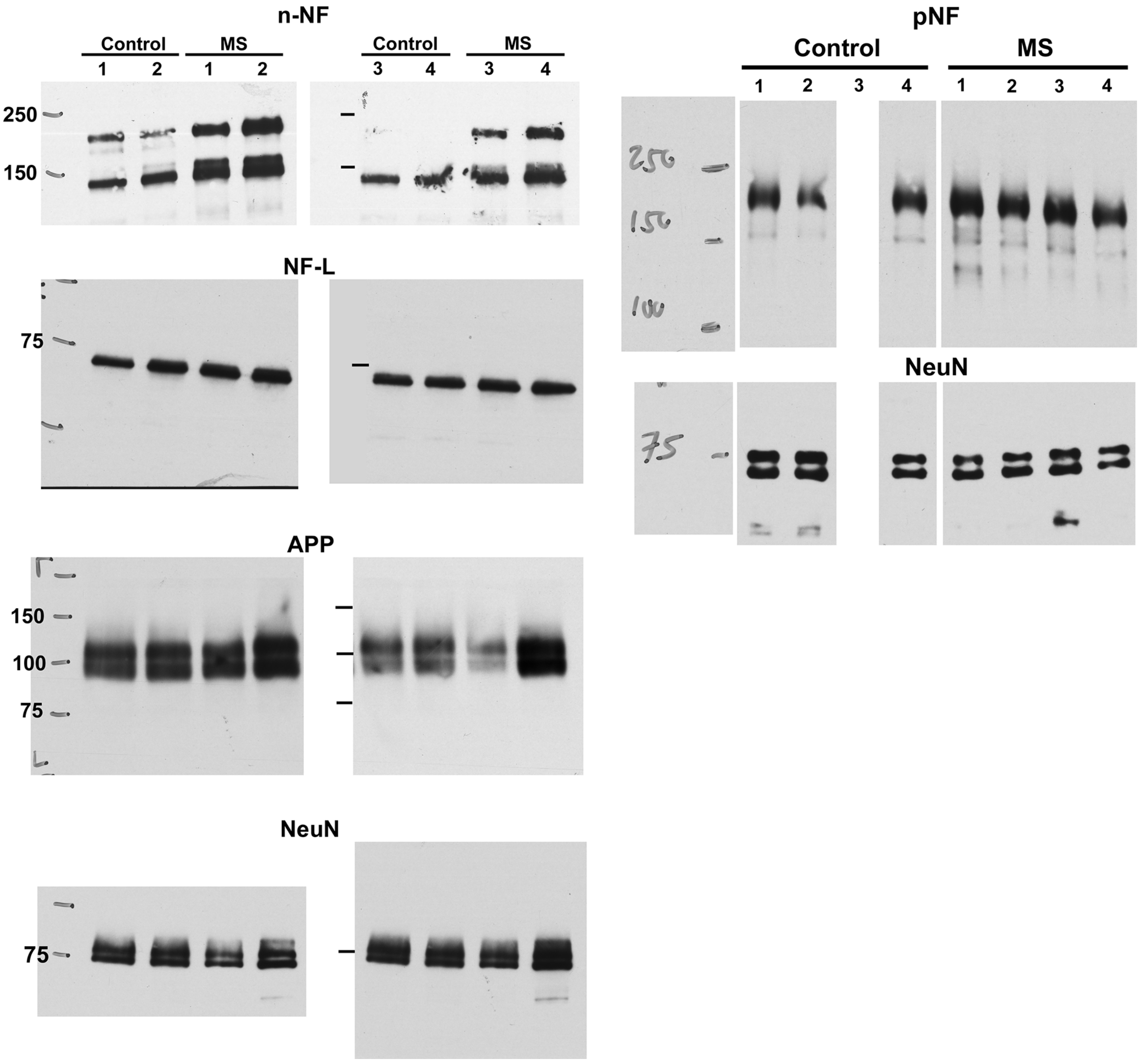

Supplementary Information
Corticohippocampal Dysfunction In The *OBiden* Model Of Primary Oligodendrogliopathy
Daniel Z. Radecki, Elizabeth L. Johnson, Ashley K. Brown, Nicholas T. Meshkin, Shane A. Perrine and Alexander Gow

Blots for Figure 7Ba
Control: lane 1;MS: lane 5

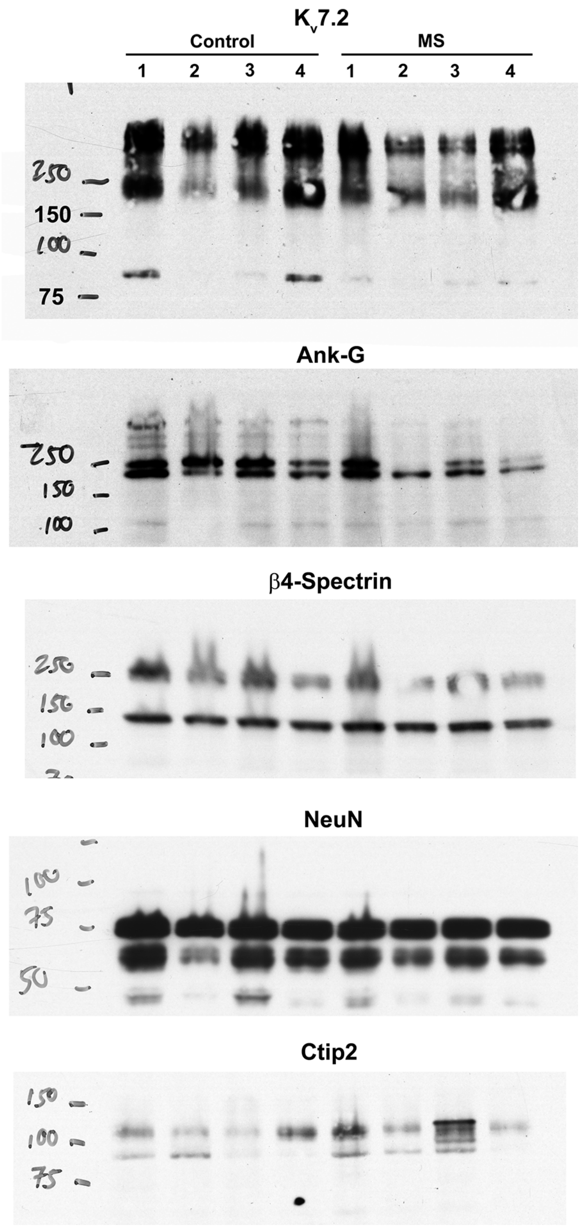

Supplementary Information
Corticohippocampal Dysfunction In The *OBiden* Model Of Primary Oligodendrogliopathy
Daniel Z. Radecki, Elizabeth L. Johnson, Ashley K. Brown, Nicholas T. Meshkin, Shane A. Perrine and Alexander Gow

Blots for Supplementary Figure 1Ca
Control: lanes 2&3; *OBiden*: lanes 7&8; $Plp1^{i.msd}$: lanes 9&11

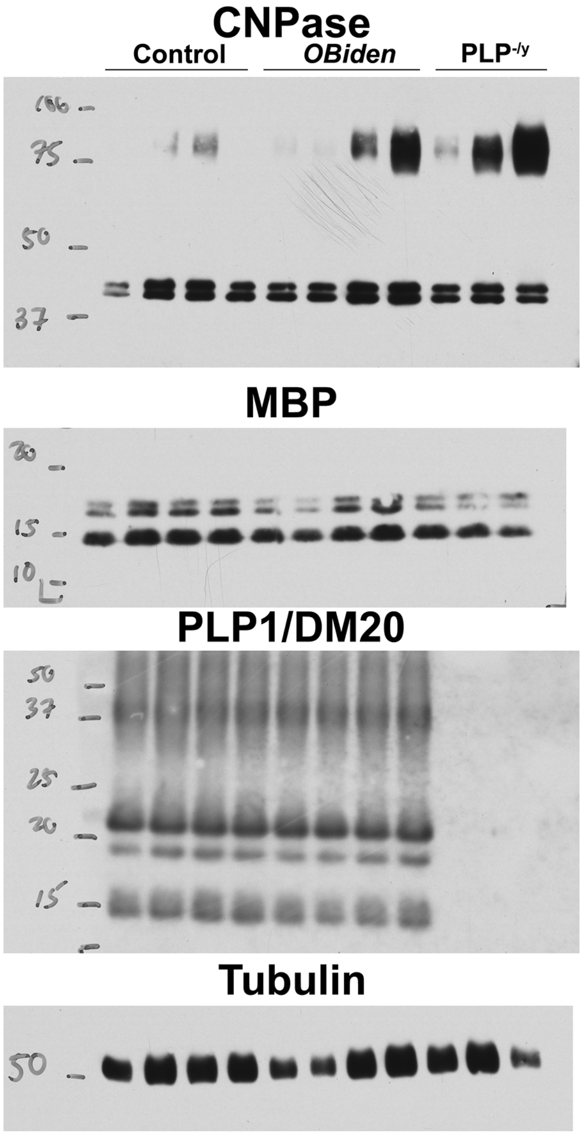

Supplementary Information
Corticohippocampal Dysfunction In The *OBiden* Model Of Primary Oligodendrogliopathy
Daniel Z. Radecki, Elizabeth L. Johnson, Ashley K. Brown, Nicholas T. Meshkin, Shane A. Perrine and Alexander Gow

Blots for Supplementary Figure 1Da
Wild type: lanes 2&3; Control: lanes 6&7; *OBiden*: lanes 9&10

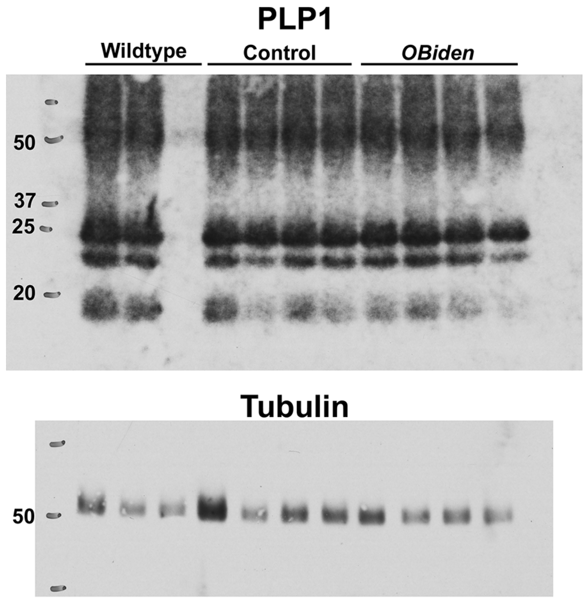

Supplementary Information

Corticohippocampal Dysfunction In The *OBiden* Model Of Primary Oligodendrogliopathy

Daniel Z. Radecki, Elizabeth L. Johnson, Ashley K. Brown, Nicholas T. Meshkin, Shane A. Perrine and Alexander Gow

Agarose gel of PCR products for Supplementary Figure 1G

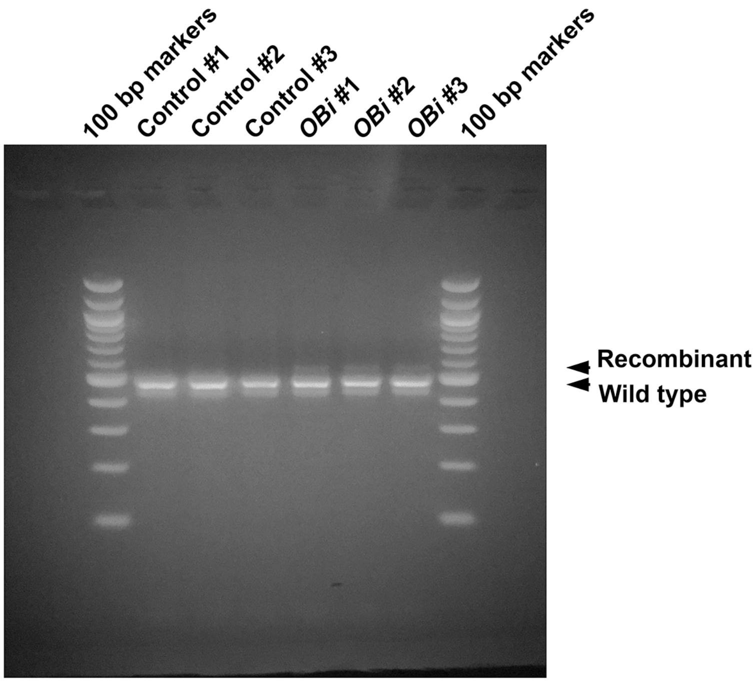

Supplement: Supplementary file 1 — Supplementary Information [file 41598_2018_34414_MOESM1_ESM.pdf]
